# Integrative multi-omics analysis reveals a novel subtype of hepatocellular carcinoma with biological and clinical relevance

**DOI:** 10.3389/fimmu.2024.1517312

**Published:** 2024-12-06

**Authors:** Shizhou Li, Yan Lin, Xing Gao, Dandan Zeng, Weijie Cen, Yuejiao Su, Jingting Su, Can Zeng, Zhenbo Huang, Haoyu Zeng, Shilin Huang, Minchao Tang, Xiaoqing Li, Min Luo, Zhihu Huang, Rong Liang, Jiazhou Ye

**Affiliations:** ^1^ Department of Hepatobiliary Surgery, Guangxi Medical University Cancer Hospital, Nanning, Guangxi, ;China; ^2^ Department of Medical Oncology, Guangxi Medical University Cancer Hospital, Nanning, Guangxi, ;China; ^3^ Department of Clinical Laboratory, Minzu Hospital Guangxi Zhuang Autonomous Region, Affiliated Minzu Hospital of Guangxi Medical University, Nanning, Guangxi, ;China

**Keywords:** hepatocellular carcinoma, tumor purity, tumor microenvironment, single-cell RNA sequencing, spatial transcriptomics, immunotherapy, precision medicine

## Abstract

**Background:**

Hepatocellular carcinoma (HCC) is a highly heterogeneous tumor, and the development of accurate predictive models for prognosis and drug sensitivity remains challenging.

**Methods:**

We integrated laboratory data and public cohorts to conduct a multi-omics analysis of HCC, which included bulk RNA sequencing, proteomic analysis, single-cell RNA sequencing (scRNA-seq), spatial transcriptomics sequencing (ST-seq), and genome sequencing. We constructed a tumor purity (TP) and tumor microenvironment (TME) prognostic risk model. Proteomic analysis validated the TP-TME-related signatures. Joint analysis of scRNA-seq and ST-seq revealed characteristic clusters associated with TP high-risk subtypes, and immunohistochemistry confirmed the expression of key genes. We conducted functional enrichment analysis, transcription factor activity inference, cell-cell interaction, drug efficacy analysis, and mutation information analysis to identify a novel subtype of HCC.

**Results:**

Our analyses constructed a robust HCC prognostic risk prediction model. The patients with TP-TME high-risk subtypes predominantly exhibit hypoxia and activation of the Wnt/beta-catenin, Notch, and TGF-beta signaling pathways. Furthermore, we identified a novel subtype, XPO1+Epithelial. This subtype expresses signatures of the TP risk subtype and aligns with the biological behavior of high-risk patients. Additional analyses revealed that XPO1+Epithelial is influenced primarily by fibroblasts via ligand-receptor interactions, such as FN1-(ITGAV+ITGB1), and constitute a significant component of the TP-TME subtype. Moreover, XPO1+Epithelial interact with monocytes/macrophages, T/NK cells, and endothelial cells through ligand-receptor pairs, including MIF-(CD74+CXCR4), MIF-(CD74+CD44), and VEGFA-VEGFR1R2, respectively, thereby promoting the recruitment of immune-suppressive cells and angiogenesis. The ST-seq cohort treated with Tyrosine Kinase Inhibitors (TKIs) and Programmed Cell Death Protein 1 (PD-1) presented elevated levels of TP and TME risk subtype signature genes, as well as XPO1+Epithelial, T-cell, and endothelial cell infiltration in the treatment response group. Drug sensitivity analyses indicated that TP-TME high-risk subtypes, including sorafenib and pembrolizumab, were associated with sensitivity to multiple drugs. Further exploratory analyses revealed that CTLA4, PDCD1, and the cancer antigens MSLN, MUC1, EPCAM, and PROM1 presented significantly increase expression levels in the high-risk subtype group.

**Conclusions:**

This study constructed a robust prognostic model for HCC and identified novel subgroups at the single-cell level, potentially assisting in the assessment of prognostic risk for HCC patients and facilitating personalized drug therapy.

## 1 Introduction

Hepatocellular carcinoma (HCC) is the most prevalent primary liver cancer, ranking as the sixth most common tumor and the third leading cause of cancer-related mortality (1, 2). The progression of HCC is a complex, multifactorial, and multistep process that involves the accumulation of genomic alterations in somatic driver genes, in addition to epigenetic changes, resulting in significant molecular heterogeneity. Therefore, understanding the molecular mechanisms driving this heterogeneity is crucial for the development of targeted therapies (3–5).

Current staging and subtyping systems for HCC primarily rely on radiological, serological, and pathological assessments of the tumor load (6). However, HCC at the same stage can exhibit distinct molecular characteristics (7), highlighting the need for more precise subtyping systems that can better predict prognosis and treatment response. Tumor tissues consist not only tumor cells but also non-tumor cells, including immune cells, and stromal cells, all of which collectively influence tumor development (8). Tumor purity (TP) is defined as the proportion of tumor cells relative to the total cell population in a sample (9). Research has shown that TP is significantly correlated with various clinical characteristics, genomic expression, and the biological properties of patients with tumors (10, 11). Furthermore, heterogeneity of the tumor microenvironment (TME) is a key contributor to tumor diversity in HCC (12, 75). Persistent tumor stimulation affects the remodeling of the TME, which subsequently influences the response of tumors to various treatments (13, 76). Targeting the TME is considered a promising strategy to overcome barriers to anticancer immune responses and enhance the efficacy of immunotherapy. With rapid advancements in high-throughput sequencing and single-cell sequencing (scRNA-seq), numerous approaches have been developed to identify disease biomarkers, leading to significant progress in disease prognosis prediction (14–16). However, only a few molecular classifications of HCC have integrated both malignant cells and TME-associated molecules. In recent years, single-cell histological studies, particularly those employing scRNA-seq technology, have substantially enhanced our understanding of tumor cell heterogeneity, tumor-infiltrating immune cell clusters, and tumor-associated stromal cell characteristics at the single-cell level (17). Nevertheless, the ability of scRNA-seq to investigate tumor spatial structure is limited because of the loss of spatial and morphological information when tissues are dissociated into single-cell suspensions. The advent of spatial transcriptomics sequencing (ST-seq) has addressed the limitations of scRNA-seq, enabling the exploration of the spatial architecture of tumors (18).

In this study, we first established a novel prognostic model for HCC via bulk RNA sequencing, which was based on the expression patterns of TP-related and TME-related genes. The expression levels of these TP and TME-related genes were subsequently validated through proteomic analysis. We then conducted an in-depth exploration of the expression patterns and biological functions of the characteristic genes associated with TP risk subtypes via scRNA-seq and ST-seq. Notably, we identified XPO1+Epithelial within the tumor that may promote tumor progression and contribute to the regulation of the TME through cellular communication networks. Finally, we conducted a preliminary assessment of the relevance and potential mechanisms of the TP-TME risk subtypes in relation to HCC targeting and immunotherapy.

## 2 Materials and methods

### 2.1 Data processing

Six HCC samples were obtained from six patients who underwent hepatectomy as the initial treatment, along with one normal liver sample provided by a hepatic hemangioma patient through surgical resection, at the Cancer Hospital of Guangxi Medical University. These samples were utilized for proteomic analysis, scRNA-seq, and ST-seq. The patients were enrolled at the Cancer Hospital of Guangxi Medical University from June to September 2021. Detailed information on the diagnostic criteria for HCC, along with patient inclusion and exclusion criteria, has been reported previously (19). In summary, all enrolled patients with HCC were newly diagnosed, pathologically confirmed, and free from other cancer types. Additionally, tumor and adjacent tissues were collected from 40 HCC patients who were diagnosed and treated with radical surgery between January 2021 and January 2024 at the Cancer Hospital of Guangxi Medical University for immunohistochemical (IHC) experiments. The detailed clinical information is present in **Supplementary Table S1**.

We screened the HCCDB database (http://lifeome.net/database/hccdb/home.html) (20) to identify the candidate datasets. The inclusion criteria were as follows: 1) the dataset included both gene expression profiles and the prognosis of patients with HCC, 2) the number of patients with a survival of more than 30 days should be more than 100, and 3) the gene expression profile of the dataset should contain more than 10,000 genes. In the HCCDB database, four datasets met the above criteria. We selected and downloaded the three largest datasets by sample size (GSE14520_GPL3921, TCGA-LIHC, and LIRI-JP) for analysis. The dataset GSE14520_GPL3921 (21) containing 225 HCC and 220 tumor-adjacent liver tissue samples was utilized to develop our subtyping systems. The TCGA-LIHC dataset, which contains RNA-seq data and clinical information for 356 HCC patients from The Cancer Genome Atlas (TCGA) (https://www.cancer.gov/tcga), and the LIRI-JP data set containing RNA-seq data and clinical information for 212 HCC patients from the JP Project from the International Cancer Genome Consortium (https://dcc.icgc.org/), were used to validate the subtyping systems.

**Figure 1:**
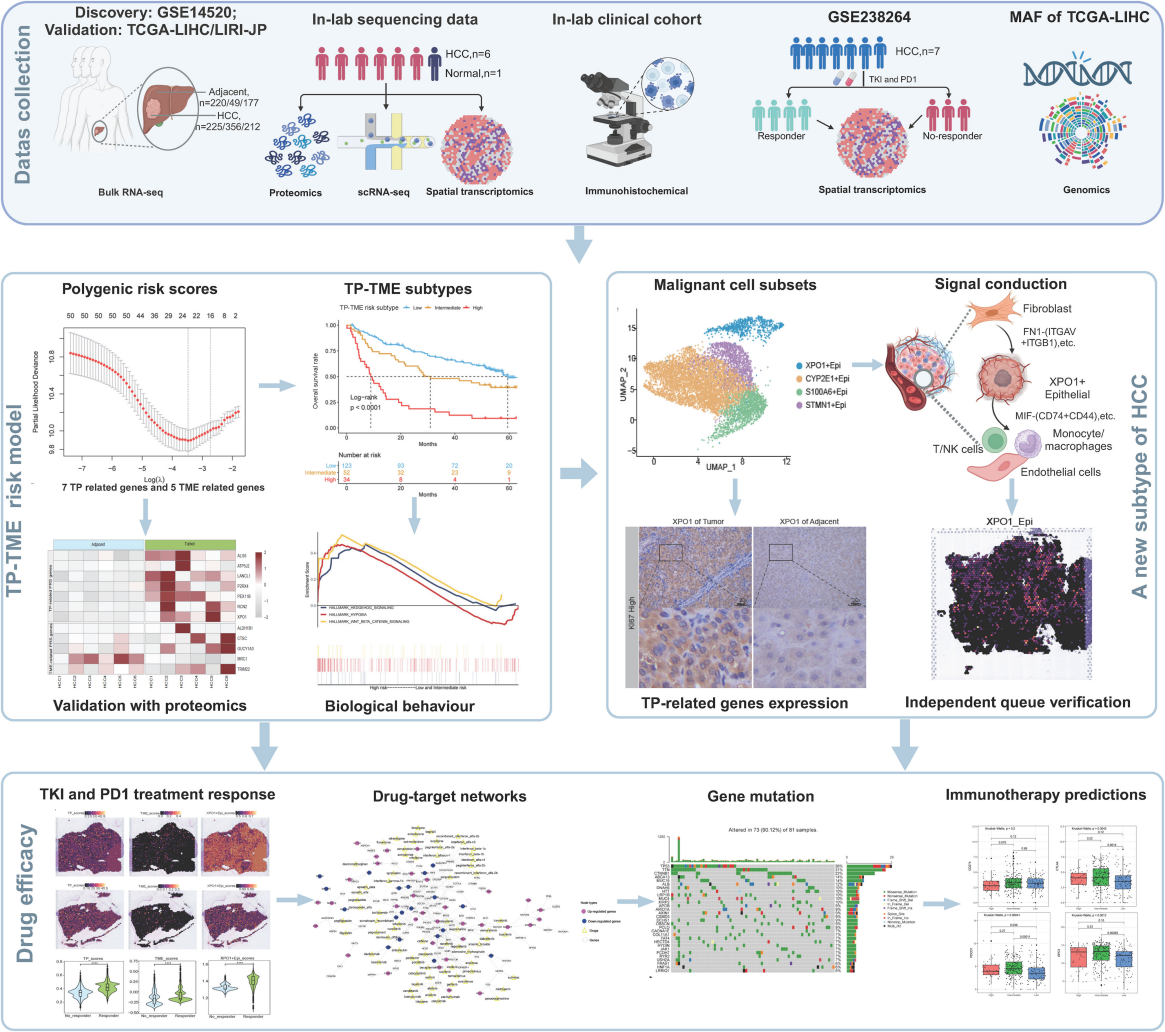
A flowchart showing the overall idea of this study. The figure was created with biorender.com.

Finally, we gathered proteomic analysis data of tumor and tumor adjacent tissues from 159 cases of HBV-associated HCC reported in Gao’s study (22). This served as a validation of the proteomic analysis to confirm our findings. We also collected the ST-seq cohort GSE238264 (23), which was diagnosed with HCC and treated with a combination of tyrosine kinase inhibitors (TKIs) and programmed cell death protein 1 (PD-1) inhibitors, serving as a validation cohort sourced from the GEO database. The workflow of the present study is illustrated in **Figure 1**.

### 2.2 Calculation of the TP and TME scores and identification of differentially expressed genes

The gene expression profiles of GSE14520_GPL3921 were first utilized to calculate TP via the ESTIMATE package (24). GSE14520_GPL3921 was also used to calculate the TME score via the xCell tool (https://xcell.ucsf.edu/) (25) with the xCell gene signature. The DEGs in HCC compared to tumor-adjacent liver tissue were identified via the R package limma (v3.54.2) (26). Genes with fold changes > 1.5 and P (adjusted by false discovery rate) values < 0.05 were considered significant.

### 2.3 Normality test and correlation analysis

The TP and TME scores were separately analyzed via the Shapiro-Wilk test. Spearman or Pearson correlation analyses were performed to calculate the correlation between DEGs and the TP and the TME scores. A DEG that was positively correlated with TP and negatively correlated with the TME score was considered a TP-related gene, whereas a DEG that was negatively correlated with TP and positively correlated the TME score was considered a TME-related gene. In addition, the TME-related genes do not include marker genes of TME cells in the xCell signature.

### 2.4 Functional and pathway enrichment analysis

The online Metascape tool (https://metascape.org/) was used for functional enrichment analysis of DEGs in HCC and tumor-adjacent liver tissues. We performed GSEA (27, 28) on the GSE14520_GPL3921 dataset via the GSEA Java software (http://www.gsea-msigdb.org/gsea/index.jsp). Hallmark and canonical pathway gene sets derived from the Kyoto Encyclopedia of Genes and Genomes (KEGG) pathway database were downloaded from the Molecular Signatures Database (MSigDB) (28, 29) and used as reference gene sets. The threshold was set to a nominal P (NOM P) value < 0.05 and FDR q value < 0.25. Functional enrichment analyses of DEGs between single-cell clusters were performed via the R package clusterProfiler (v.4.6.2) (29), which is based on the Gene Ontology (GO) or MsigDB. The corrected enrichment terms with P<0.05 were considered statistically significant. Functional scoring was performed via AddModuleScore in the R package Seurat for gene sets in the MsigDB. Functional enrichment analyses of DEGs among single-cell clusters were conducted via the R package clusterProfiler (version 4.6.2) (30), which utilizes the GO framework or data from MsigDB. Enrichment terms with a corrected p-value of less than 0.05 were deemed statistically significant. Additionally, functional scoring was performed via the AddModuleScore function in the R package Seurat for gene sets derived from MsigDB.

### 2.5 Protein-protein interaction networks

The PPI networks of TP-related and TME-related genes were obtained from the STRING database (v11.5) (31) to preliminarily reveal the crosstalk between tumor cells and TME. The interactions with high confidence (>0.7) were included in the present study and visualized via Cytoscape software (v 3.8.0) (32).

### 2.6 Development of the TP- and TME-related gene-based polygenic risk scores

First, to develop the TP-related polygenic risk score (PRS), overall survival (OS)-associated TP-related genes were identified via univariate Cox regression analysis. Second, the expression profiles of the OS-associated TP-related genes were used to carry out least absolute shrinkage and selection operator (LASSO) Cox regression model analysis with leave-one-out cross validation via the glmnet package (33). The genes with nonzero coefficients were considered the optimal features and subjected to multivariate Cox regression and stepwise regression analysis. The TP-related PRS was subsequently developed via the following formula: TP-related PRS = Σ (Expression_i_ ∗ Coeffient_i_) where “Coeffient” and “Expression” represent the risk coefficient and expression of each gene in the multivariate Cox regression and stepwise regression analysis, respectively. The TME-related PRS was also developed according to the same method as above.

### 2.7 The TP-TME subtypes of HCC

The optimal cutoffs of the TP-related and TME-related PRS were identified vis the surv_cutpoint function from the Survminer package (https://CRAN.R-project.org/package=survminer) to separately divide patients into high and low TP- and TME-related PRS groups. Each individual received a TP- and a TME-related PRS levels, and we developed the TP-TME subtype according to the TP- and TME-related PRS levels. Patients with high TP- and TME-related PRS were considered the high-risk subtype, those possessing low TP- and TME-related PRS were considered the low-risk subtype, and the remaining patients with high TP-related and low TME-related PRS or a low TP-related and high TME-related PRS were considered the intermediate-risk subtype.

### 2.8 Proteomic analysis

The protein samples were extracted, digested, and labeled with Tandem Mass Tag (TMT) according to the experimental specifications. A 10 μL aliquot of the supernatant was injected into a nanoflow HPLC system (Thermo Scientific) linked to an Orbitrap Fusion Lumos mass spectrometer (Thermo Scientific). The extracts were then applied to an Acclaim PepMap100 C18 column and separated on an EASY-Spray C18 column. In the Orbitrap mode, the mass spectrometer performed a comprehensive mass spectrometry (MS) scan across the 300-1500 m/z range in positive ion mode (with a source voltage fixed at 2.1 kV) and achieved a resolution of 120,000. After the complete MS scan, the 20 most abundant ions with different charge states were selected for high-energy collisional dissociation fragmentation analysis. For this experiment, the UniProt HUMAN database, which was downloaded on April 20, 2019, served as the database. MS/MS data were analyzed via Proteome Discoverer 1.4.

### 2.9 Preprocessing and quality control of single-cell transcriptome data

Raw FASTQ data were processed via Cell Ranger (version 8.0.0; 10× Genomics, USA), generating gene count matrices on the basis of the human genome reference set GRCh38 with all default parameter settings. The output filtered gene expression matrix for each sample was analyzed via the R package Seurat (v.4.3.0) (34). We calculated the doublet fraction for each cell via the R package DoubletFinder (v.2.0.4) (35) with aim of removing potential doublets with a target value of 7.6% per 1000 droplets. Additionally, for each sample, cells with fewer than 300 unique molecular identifiers (UMIs), or expressing more than 7000 or fewer than 300 genes were excluded. To eliminate dead or dying cells, we further removed cells with more than 10% UMIs originating from the mitochondrial genome. Next, the “FindVariableFeatures” function in Seurat and the vst method were employed to screen for the 2000 variable genes exhibiting the largest normalized variance, which were subsequently processed for principal component analysis (PCA). The “RunHarmony” function from the Harmony package (v.1.2.0) (36) was then utilized for sample batch correction. The “RunUMAP” function was applied to perform UMAP downscaling of the first 20 principal components according to the aforementioned steps. Finally, the “FindNeighbors” and “FindClusters” functions were employed to identify cell clusters.

### 2.10 Cell type identification

We collected typical marker genes for the identification of the major cell types. Epithelial cells were identified by the expression of ALB, APOA2, and EPCAM; fibroblasts were characterized by COL1A1, COL1A2, and DCN; endothelial cells were identified via PECAM1, VWF, and PLVAP; T/NK cells were marked via CD3D, CD3E, and CD2; B cells were identified via CD79A and MZB1; monocytes/macrophages were characterized via LYZ, CD86, and C1QC; mast cells were identified via TPSB2, CPA3, and KIT; neutrophils were marked via G0S2, CXCL8, and CSF3R; and dividing cells were identified via MKI67 and STMN1.

### 2.11 Immunohistochemistry experiments

Following tissue collection, samples from patients were fixed in 10% formaldehyde for 12 hours. The tissues were subsequently dehydrated, clarified, paraffin embedded, and sectioned at a thickness of 4 µm for both H&E and IHC staining. The staining procedures were performed according to the manufacturer’s guidelines. Sections stained with H&E were examined under a light microscope (OLYMPUS BX43) via a ×10 eyepiece and a ×40 objective lens, with images captured using ImageView 4.15 (Pooher) software. For immunohistochemical analysis, the sections were scanned with a Pannoramic digital section scanner (3DHISTECH). Two pathologists, blinded to the clinical characteristics and findings of the patients, independently evaluated all the sections. Scoring was conducted on a 4-point scale on the basis of the intensity of cellular staining: no positive staining (negative) received a score of 0, yellowish (weakly positive) scored a score of 1, tan (positive) scored a score of 2, and tan (strongly positive) scored a score of 3. Additionally, a 4-point scale based on the percentage of positive cells was employed, with ≤25% scoring 1, 26%-50% scoring 2, 51%-75% scoring 3, and >75% scoring 4. The final score was derived by multiplying the two individual scores. The following primary antibodies were used to bind specific IHC proteins: XPO1 (Proteintech, 27917-1-AP) and RCN2 (Proteintech, 10193-2-AP), and the secondary antibody used was horse anti-mouse/rabbit IgG (Vector, ZF1028). Raw data for 40 HCC patients were uploaded (**Supplementary File 1**).

### 2.12 Chromosome copy number variation analysis

The R package InferCNV (v1.21.0) (https://github.com/broadinstitute/inferCNV) was used to infer CNV changes in the scRNA-seq data. The raw gene expression counts extracted from the Seurat object were imported into the Infercnv object via the “InfercnvObject()” function. T/NK cells and B cells were selected as control datasets for reference. The CNV value for each cell was estimated via the “run()” function in InferCNV with a cutoff value of 0.1.

### 2.13 Transcription factors activity analyses

Activity analyses of TFs were performed to pinpoint key regulatory TFs in the selected cell clusters, and SCENIC analysis was conducted via the pySCENIC (37) package. The necessary databases for executing SCENIC, which include the TF database (cisTarget.hg38.mc9nr.feather) and the subject annotation database (hgnc.v9.1.0), were acquired from the pySCENIC website (https://github.com/aertslab/pySCENIC). The normalized expression matrix generated by Seurat served as the input matrix for the pySCENIC. TF activity was determined by the area under the recovery curve (AUC), and detailed findings from the transcription factor activity analyses are shown in **Supplementary Table S2**.

### 2.14 Cell-cell communication analyses

Cell-cell communication was inferred via the R software version of CellChat (v1.6.1) (38) and an existing database of ligand-receptor interactions. The apparent overexpression of ligands and receptors in specific cell clusters was initially identified via CellChat. The probability of communication occurring between two interacting clusters was quantified on the basis of the average expression level of the ligand in one cluster and the average expression level of the receptor in the other. The significance of communication was assessed via a permutation test. Interaction pairs with a P value < 0.05 were selected to assess intercellular communication. The detailed results of the cell-cell communication analysis are listed in **Supplementary Table S3**.

### 2.15 Spatial transcriptome data analyses

The expression matrices from the ST-seq data were processed via Seurat. “SCTransform()” normalised the values at each point, and “RunPCA()” retained the first 20 principal components (PCs) to reduce dimensionality. The top 30 DEGs for each cell cluster in the scRNA-seq cohort were used as input. Scores were assigned to individual points in the ST-seq dataset via the “gsva()” function of the R package GSVA (v1.46) (39) with default parameters. The spatial feature expression plots were generated via the “SpatialFeaturePlot()” function in Seurat.

### 2.16 Analyses of gene mutations and stemness scores

Gene mutation data from the TCGA-LIHC dataset were extracted from mutation annotation format (MAF) files via the *GDCquery_Maf* function in the “TCGAbiolinks” package (40). The gene mutation frequencies of each risk subtype were visualized as a waterfall plot via the *oncoplot* function in the “TCGAbiolinks” package. The tumor mutational burden (TMB) of each sample was obtained from a previous study (41). The stemness score (42) was calculated for each individual in the TCGA-LIHC dataset via the TCGA analyze_Stemness function in the “TCGAbiolinks” package.

### 2.17 Prediction of the efficacy of therapy

For the TP-TME risk subtypes, we compared the expression levels of two immune checkpoints (PDL1 and CTLA4) and five antigens (CD133, EPCAM, GCP3, MSLN, and MUC1) (43) to predict the potential response to these treatments. In addition, we performed drug repositioning analysis for the high-risk subtype via the PATHOME-Drug (http://statgen.snu.ac.kr/software/pathome/) web tool.

### 2.18 Statistical analysis

Unless otherwise stated, all analyses were performed in R (version 4.2.3). We identified DEGs via unpaired t-tests provided via the limma package. The Shapiro-Wilk test was used for the normality test. Time-dependent receiver operating characteristic (tROC) curve analysis was carried out using the tROC package (44). Kaplan–Meier survival curves for OS and progression free survival (PFS) were compared in different subtypes using the log-rank method in the “survival” package (https://CRAN.R-project.org/package=survival) and the “survminer” package (https://CRAN.R-project.org/package=survminer). Intergroup differences in continuous variables were assessed for significance via Wilcoxon, Kruskal–Wallis, or unpaired t tests. All tests were two-sided, and unless otherwise stated, we set P value < 0.05 to indicate statistical significance. Visualization was done vis the “ggplot2” R package.

## 3 Results

### 3.1 Biological functions and interactions of TP-related and TME-related genes

**Figure 2:**
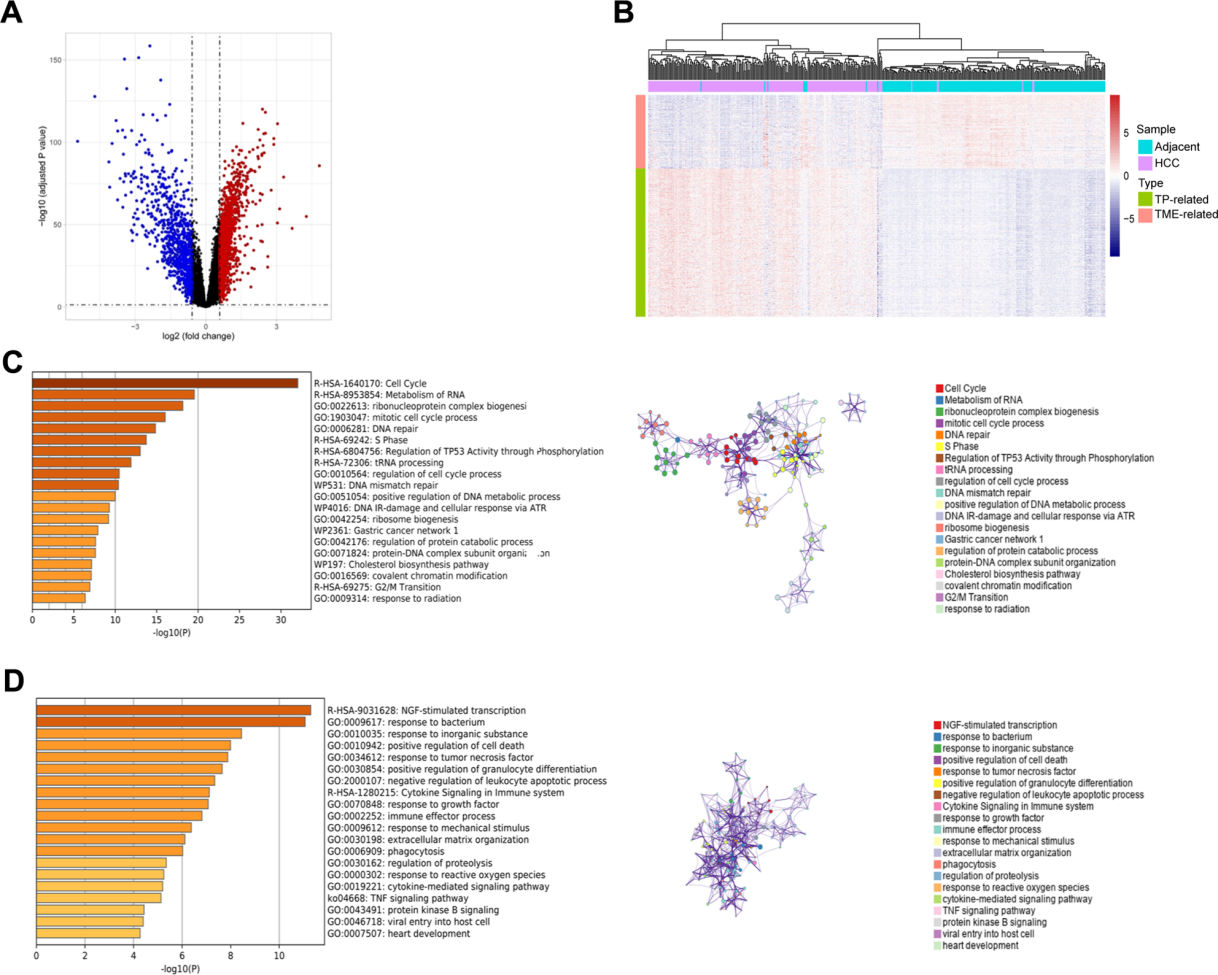
Identification, enrichment analysis, and protein-protein interaction networks of the tumor purity-related genes and tumor microenvironment-related genes in GSE14520_GPL3921. **(A)** Volcano plot of the differentially expressed genes. **(B)** Hierarchical clustering showing that the expression patterns of TP-related and TME-related genes basically distinguish HCC and tumor-adjacent liver tissues. **(C)** Functional enrichment analysis of the TP-related genes. *Left panel:* GO terms and pathways involving TP-related genes, and *right panel*: interactions among the GO terms and pathways. **(D)** Functional enrichment analysis of TME-related genes. *Left panel:* GO terms and pathways involving TME-related genes, and *right panel*: interactions among the GO terms and pathways.

Using the GSE14520_GPL3921 dataset, we identified a total of 2,263 differentially expressed genes (DEGs) in HCC compared with tumor-adjacent liver tissue (**Figure 2A**). A total of 451 TP-related genes and 121 TME-related genes were identified through Spearman correlation analysis. Bidirectional hierarchical clustering revealed that the expression patterns of these genes could largely distinguish between HCC and tumor-adjacent liver tissue (**Figure 2B**). Unsurprisingly, the TP-related genes were enriched in mainly cancer-related Gene Ontology (GO) terms and pathways, such as the cell cycle and mismatch repair (**Figure 2C**). In contrast, TME-related genes were associated primarily with immune system processes (**Figure 2D**). The PPI networks of the TP-related and TME-related genes contain 342 nodes and 1177 edges (**Supplementary Figure S1**). In the PPI networks, red nodes indicate genes whose expression is upregulated, and blue nodes indicate genes whose expression is downregulated in tumors. Circular nodes represent TP-related genes, whereas rhombic nodes represent TME-related genes.

### 3.2 The TP-TME risk subtype is a robust prognostic prediction system

**Figure 3:**
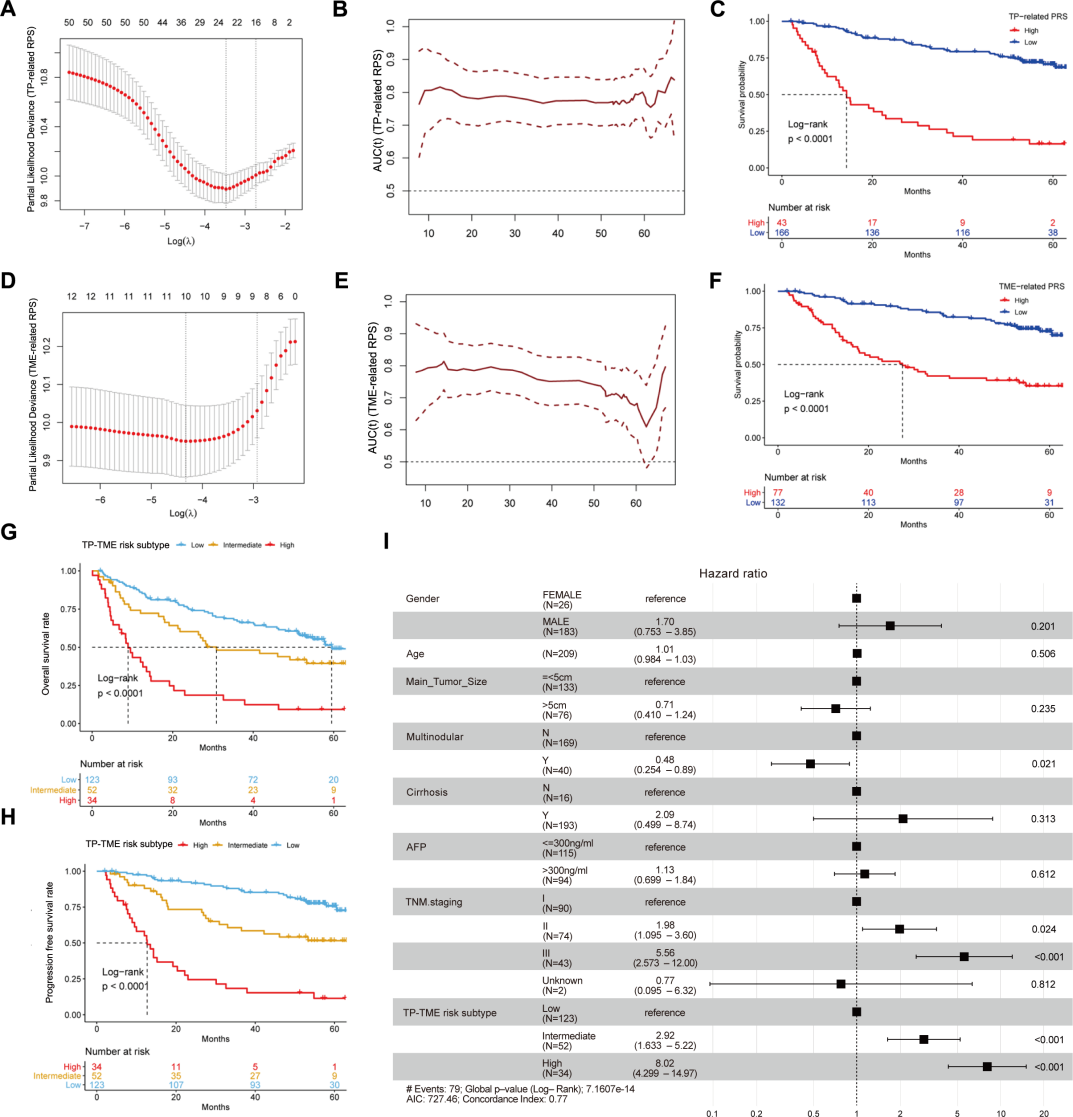
Development and validation of TP-TME risk subtypes in GSE14520_GPL3921. **(A)** Twenty-two TP-related genes had nonzero coefficients in the LASSO Cox regression model analysis; **(B)** Time-dependent ROC curve analysis for the TP-related PRS; **(C)** HCC with high TP-related PRS had shorter overall survival than those with low TP-related PRS. **(D)** Ten TME-related genes had nonzero coefficients in the LASSO Cox regression model analysis; **(E)** Time-dependent ROC curve analysis for the TME-related polygenic risk score; **(F)** HCC with high TME-related PRS had shorter overall survival than those with low TME-related PRS. **(G)** There were significant differences in overall survival among the three subtypes of the TP-TME risk subtypes. **(H)** There were significantly differences in progression-free survival among the three subtypes of the TP-TME risk subtypes. **(I)** The TP-TME risk subtype system was proven to be an independent prognostic factor, after adjusting for other clinicopathological characteristics.

Fifty TP-related genes were identified as OS-associated genes, twenty-two of which presented nonzero coefficients (**Figure 3A**), and 11 genes (ALG6, ATP5MF, CNIH4, ESM1, HEY1, LANCL1, P2RX4, PEX11B, POP7, RCN2, and XPO1) were used to generate the TP-related PRS (**Supplementary Table S4**). The TP-related PRS was significantly associated with OS [P < 0.0001, hazard ratio (HR) = 2.718 (95% CI for HR = 2.147-3.442)], and the area under the curve (AUC) of the tROC analysis was stable at approximately 0.8 (**Figure 3B**). The HCC patients with high TP-related PRS had shorter OS than did those with low TP-related PRS (P < 0.0001) (**Figure 3C**). Among the TME-related genes, twelve genes were identified as OS-associated genes, ten of which had nonzero coefficients (**Figure 3D**), and seven genes (ALDH1B1, CTSC, GUCY1A1, MRC1, SPRY2, TARP, and TRIM22) were used to generate the TME-related PRS (**Supplementary Table S5**). The TME-related PRS was also significantly associated with OS [P < 0.0001, HR = 2.718 (95%CI for HR = 1.978-3.735)], and the AUC of tROC analysis was 0.7-0.8 (**Figure 3E**). HCC patients with a high TME-related PRS had shorter OS than did those with low TP-related PRS (P < 0.0001) (**Figure 3F**). Our TP-TME risk subtype was generated on the basis of two PRSs, and 34, 52, and 123 patients with HCC were divided into high-, intermediate-, and low-risk subtypes, respectively. Patients in the high-risk subtype had the poorest survival, those in the low-risk subtype had the best survival, and those in the intermediate-risk cases had a better prognosis than did those in the high-risk subtype and worse prognosis than did those in the low-risk subtype did (**Figure 3G**). A similar trend was also observed for PFS (**Figure 3H**). Furthermore, the TP-TME risk subtyping system demonstrated superior prognostic predictive power compared to routine clinicopathological features and remained independent of these features (**Figure 3I**). As in the GSE14520_GPL3921 dataset, the TP-related PRS and the TME-related PRS in the TCGA-LIHC and LIRI-JP datasets were calculated according to the abovementioned formulas. We found similar results in the TCGA-LIHC cohort (**Supplementary Figures S2A–E**) and the LIRI-JP cohort (**Supplementary Figures S2F–I**). Overall, TP-TME risk subtype is a robust prognostic prediction system.

### 3.3 The biological functions of characteristic genes involved in TP-TME risk subtype

**Figure 4:**
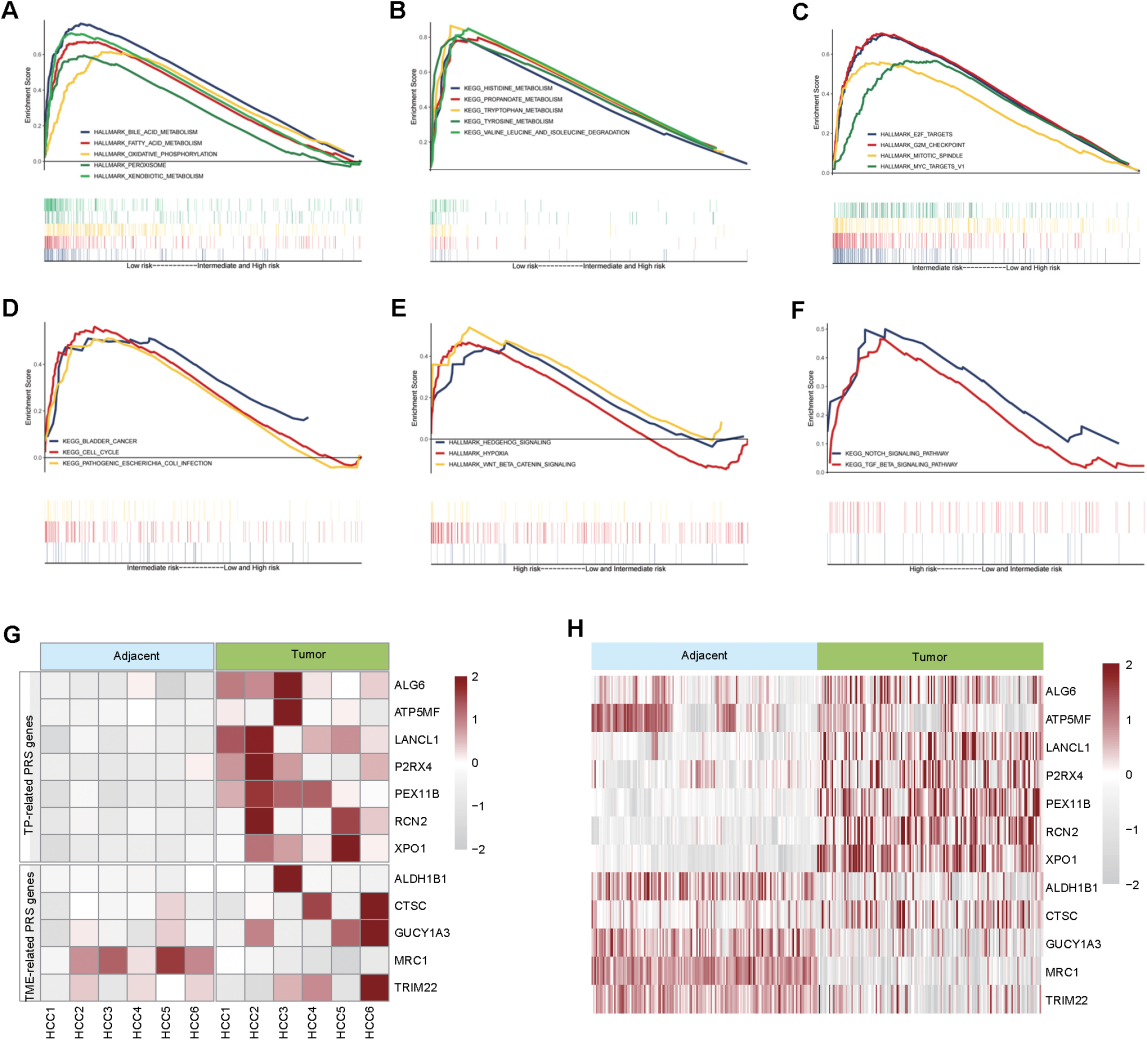
Biological behavior and characteristic gene protein expression levels of TP-TME risk subtypes. **(A)** The hallmark gene sets enriched in the TP-TME low-risk subtype. **(B)** The KEGG pathway gene sets enriched in the TP-TME low-risk subtype. **(C)** The hallmark gene sets enriched in the TP-TME intermediate-risk subtype. **(D)** The KEGG pathway gene sets enriched in the TP-TME intermediate-risk subtype. **(E)** The hallmark gene sets enriched in the TP-TME high-risk subtype. **(F)** The KEGG pathway gene sets enriched in the TP-TME high-risk subtype. **(G)** Heatmap showing the protein expression levels of genes associated with the TP-TME risk subtypes. **(H)** Heatmap showing protein expression levels of genes associated with the TP-TME risk subtypes reported by Gao et al.

Compared with those in the TP-TME intermediate- and high-risk subtypes, the liver function-related HALLMARK (**Figure 4A**) and metabolism-related KEGG (**Figure 4B**) gene sets were significantly enriched in the TP-TME low-risk subtype. These findings suggest that the TP-TME low-risk subtype of HCC is well- differentiated. The TP-TME intermediate-risk subtype was characterized by enrichment of transcription factor E2F and MYC targets (**Figure 4C**) and cell cycle pathways (**Figure 4D**), whereas the TP-TME high-risk subtype was characterized by enrichment of hypoxia, Wnt/beta-catenin signaling (**Figure 4E**), the Notch signaling pathway and the TGF-beta signaling pathway (**Figure 4F**). These results indicate that there is significant biological heterogeneity among these three subtypes.

To validate the expression of genes characterizing the TP-TME risk subtype at the protein level, we collected tumor and adjacent-tumor tissues from 6 HCC patients for proteomic analysis. Analysis of the protein expression levels of genes associated with the HCC-TP-TME risk subtype showed, that XPO1, RCN2, PEX11B, P2RX4, LANCL1, ATP5MF, ALG6, TRIM22, GUCY1A3, CTSC, and ALDH1B1 were significantly elevated in tumor tissues compared with adjacent tumor tissues (**Figure 4G**). Furthermore, we validated these findings via published data from Gao et al. (22), which corroborated our results in an independent cohort (**Figure 4H**). Collectively, these results indicate that the characteristic genes of the TP-TME risk subtypes identified in our study have important clinical implications for the prognostic assessment of HCC and warrant further investigation into the biological functions of these genes.

### 3.4 Exploring the expression patterns and biological functions of genes characterizing TP-TME risk subtypes in HCC via scRNA-seq

**Figure 5:**
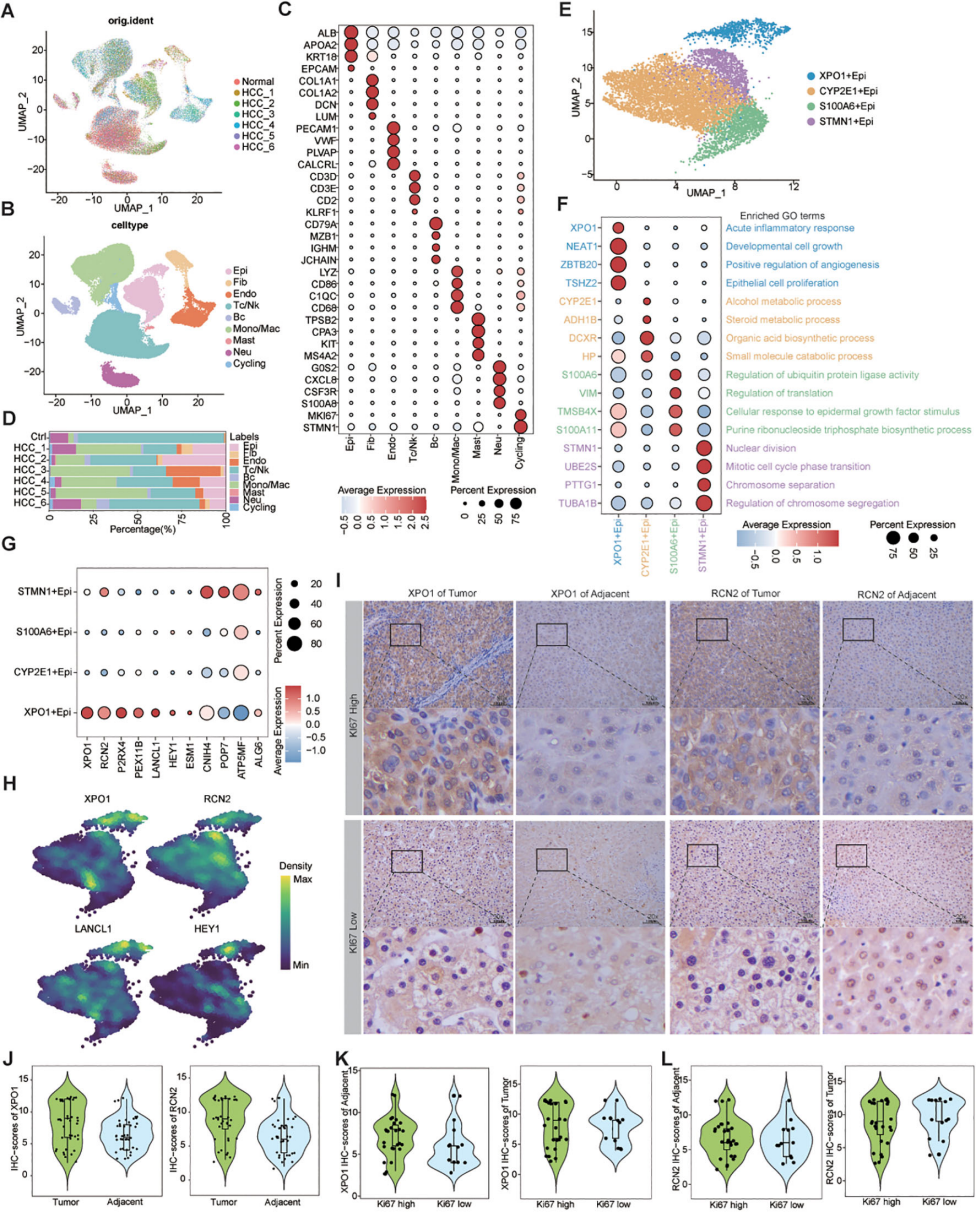
Single-cell resolution exploration of the expression profiles of genes characterizing TP-TME risk subtypes in HCC. **(A)** UMAP plot illustrating the distribution of cells across different samples, with distinct colors representing each sample; **(B)** UMAP plot depicting the transcriptomic landscape of 62,163 high-quality cells across nine cell types, with different colors indicating the various cell types: Epi (epithelial cell), Fib (fibroblast cell), Endo (endothelial cell), Tc/NK (T cell/natural killer cell), Bc (B cell), Mono/Mac (monocyte/macrophage cell), Mast (mast cell), Neu (neutrophil cell), and Cycling (cycling cell). **(C)** Bubble plots displaying the percentage expression of classical marker genes across the nine cell types, alongside average expression levels. **(D)** Bar graph illustrating the distribution of the nine cell types across different tissues, color-coded by cell type. **(E)** UMAP plot showing the cluster of epithelial cells divided into four distinct cell clusters. **(F)** Bubble plots highlighting the percentage and average expression levels of genes with high expression specific to different epithelial cell clusters, as well as GO-BP functional enrichment. **(G)** Bubble plots presenting the expression percentages and average expression levels of genes characterizing the TP risk subtype across different epithelial cell clusters. **(H)** Density map illustrating the distribution of TP-related genes within epithelial cell clusters. **(I)** IHC staining of XPO1 and RCN2 in clinical samples from HCC adjacent and tumor tissues. **(J)** Violin plot demonstrating the statistical analysis of IHC scores for XPO1 and RCN2 genes. **(K, L)** Violin plots displaying IHC scoring statistics for XPO1 **(K)** and RCN2 **(L)** under varying levels of Ki67 expression.

By analyzing the scRNA-seq data for HCC, we obtained 62,163 high-quality cells. Nine distinct cell types were identified on the basis of known markers: epithelial cells, fibroblasts, endothelial cells, T/NK cells, B cells, monocytes/macrophages, mast cells, neutrophils, and cycling cells (**Figures 5A–C**). Furthermore, we analyzed the proportions of various cell types across different patients and found that, although all cell types were present in each patient, the predominant infiltrating cell types varied, which may reflect heterogeneity among HCC patients (**Figure 5D**).

Next, we re-clustered the epithelial cells on the basis of their differentially expressed genes, identifying 4 clusters: XPO1+Epithelial, CYP2E1+Epithelial, S100A6+Epithelial, and STMN1+Epithelial (**Figure 5E**). The bubble diagram illustrates the highly expressed genes in each cluster (**Figure 5F**). Functional enrichment analysis revealed that XPO1+Epithelial was predominantly associated with the acute inflammatory response, cell growth, positive regulation of angiogenesis, and epithelial cell proliferation, indicating its potential role in tumor progression. In contrast, CYP2E1+Epithelial were primarily linked to material and energy metabolism, whereas S100A6+Epithelial were associated with the regulation of ubiquitin-protein ligase activity, among other functions. STMN1+Epithelial exhibited characteristics related to cytokinesis (**Figure 5F**). We subsequently analyzed the chromosome copy number variation in these epithelial cells via inferCNV. The results indicated that all four clusters presented significant chromosome amplifications and deletions (**Supplementary Figure S3A**). Additionally, we observed minimal capture of epithelial cells in normal liver tissue samples (**Figure 5D**), leading us to conclude that all four clusters consisted of malignant epithelial cells.

We found that 7 genes characterizing TME risk subtypes were expressed at varying levels across multiple cell types within the TME (**Supplementary Figure S3B**). However, 11 genes associated with the TP risk subtype signatures exhibited significantly increased expression in the XPO1+Epithelial. Specifically, the expression levels of XPO1, RCN2, P2RX4, PEX11B, LANCL1, HEY1, and ESM1 were significantly increase in this cluster than in the other clusters (**Figures 5G, H**). Consequently, we focused on this cluster characterized by high expression of genes in the XPO1+Epithelial group. Next, we collected tumor tissues and adjacent-tumor tissues from 40 HCC patients for immunohistochemical staining (**Figure 5I**) to validate our findings. We observed that the expression levels of XPO1 and RCN2 were elevated in tumor tissues compared with adjacent-tumor tissues (**Figure 5J**). Further analysis indicated that the expression of XPO1 and RCN2 was greater in tumor tissues with high Ki67 expression (**Figures 5I, K, L**). These results suggest that the elevated expression of XPO1 and RCN2 is closely associated with the proliferation of HCC.

**Figure 6:**
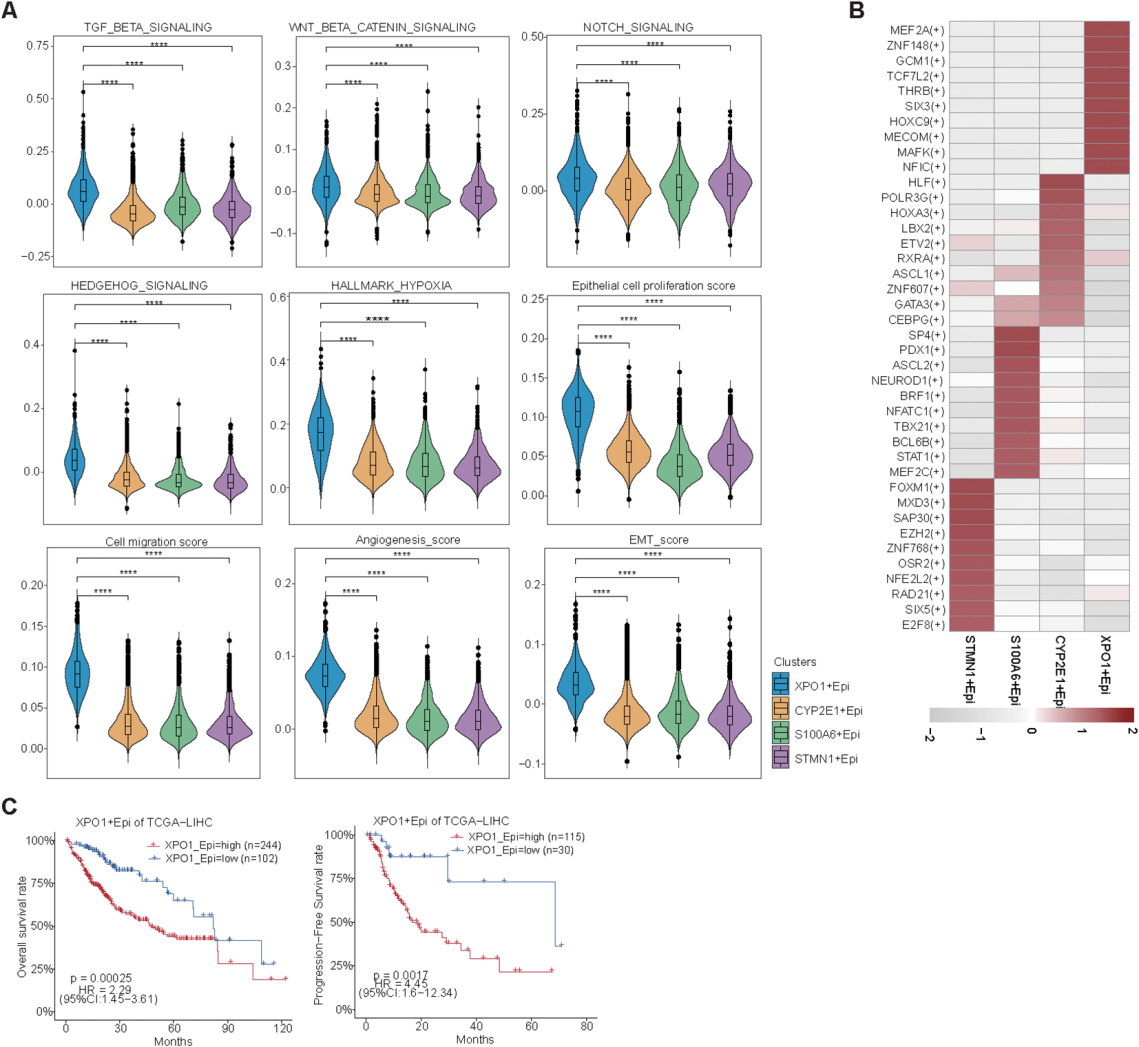
Biological function scoring and survival analysis of epithelial cells. **(A)** Violin plots illustrating various epithelial cell clusters in relation to TGF-beta signaling, Wnt beta/catenin signaling, Notch signaling, Hedgehog signaling, hypoxia, epithelial cell proliferation, cell migration, angiogenesis, and EMT scores. The Wilcoxon test was employed to evaluate the differences between groups. Statistical significance is indicated by ‘****’, corresponding to P < 0.0001, respectively. **(B)** Heatmap displaying the top 10 transcription factors with the highest activity across different epithelial cell clusters. **(C)** A line graph depicting the overall survival and progression-free survival of XPO1+ epithelial cells.

Further analysis revealed that the XPO1+Epithelial cluster scored significantly increase than other clusters did in terms of functions associated with the TGF-beta signaling pathway, WNT/beta-catenin signaling, Notch signaling, Hedgehog signaling, and hypoxia signaling (**Figure 6A**). This finding was consistent with the upregulation of these functions observed in high-risk patients within the TP-TME risk model (**Figures 4A–F**). Additionally, compared with the other clusters, the XPO1+Epithelial cluster presented significantly increase scores for proliferation, migration, epithelial-mesenchymal transition (EMT), and angiogenesis (**Figure 6A**). Our analysis of the key TFs driving the distinct functions of these clusters revealed that the ten most active transcription factors in XPO1+Epithelial were predominantly associated with tumor proliferation, migration, and invasion (**Figure 6B**). For example, MEF2A may play a dual role in promoting tumor proliferation and metastasis by inducing the activation of EMT and WNT/beta -catenin signaling (45), whereas TCF7L2 serves as a core TF of the WNT signaling pathway, and is involved in regulating tumor cell proliferation and migration (46). Furthermore, we observed that patients exhibiting high expression of XPO1+Epithelial signatures had significantly shorter OS and PFS in the TCGA cohort (**Figure 6C**).

In summary, we found that XPO1+Epithelial constitute a cluster of cells characterized by TP high-risk subtypes, which exhibit elevated expression in cancer tissues. This cluster shows higher expression levels in patients with tumors that have high proliferation rates and is positively correlated with poor patient prognosis. The underlying mechanism may involve the contribution of this cluster to tumor progression through pathways such as EMT and WNT/beta-catenin signaling.

### 3.5 Crosstalk between XPO1+ Epithelial and TME

**Figure 7:**
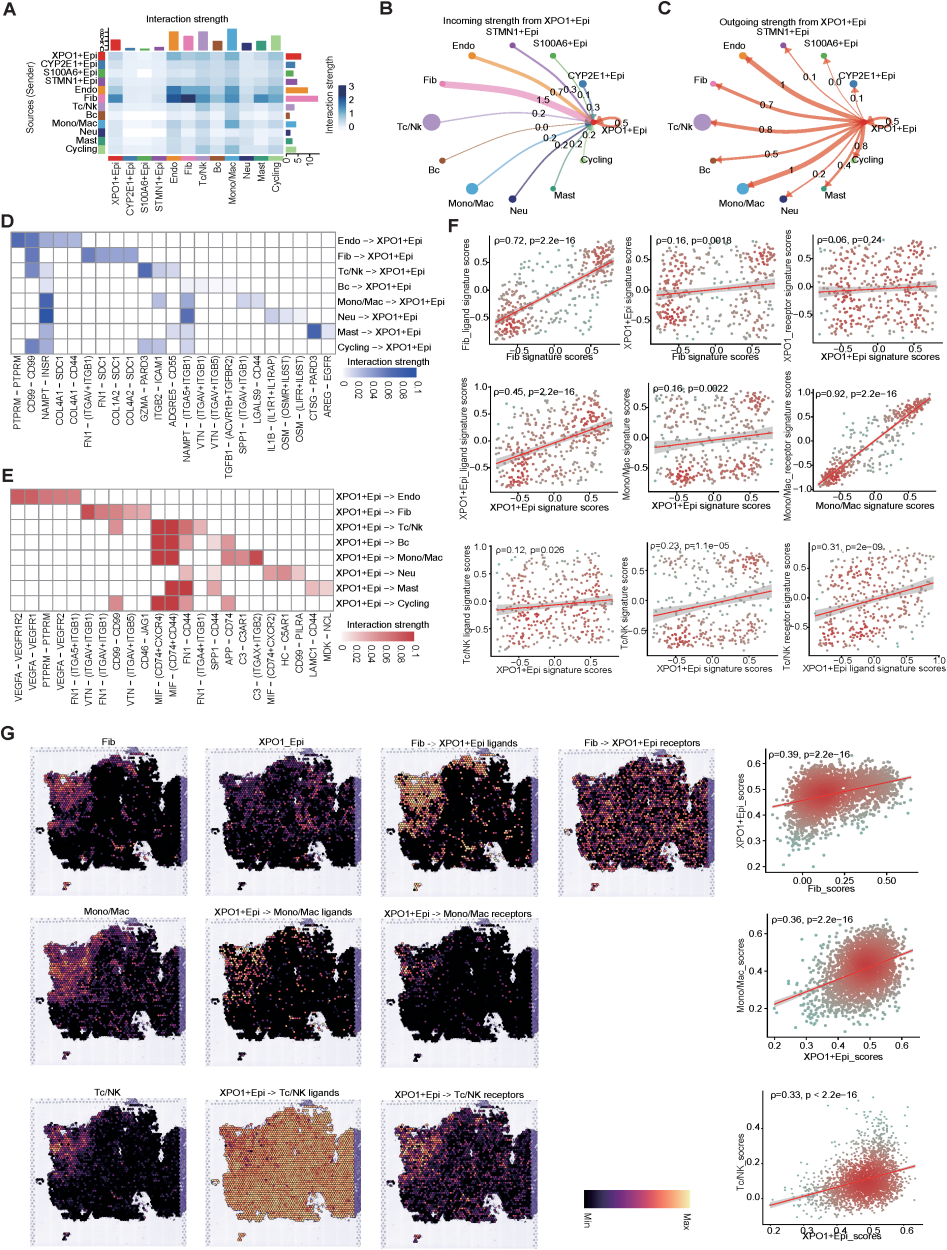
Cell-cell interaction network of HCC epithelial cells. **(A)** Heatmap illustrating the interaction intensities among various cell types in HCC. **(B, C)** Circular plot depicting the interaction intensities of incoming **(B)** and outgoing **(C)** interactions involving XPO1+Epithelial. **(D, E)** Heatmaps demonstrating the enhancement of ligand-receptor interaction intensities between XPO1+Epithelial and other cell types, with **(D)** focusing on incoming interactions and **(E)** on outgoing interactions. **(F)** A scatter plot revealing the correlation between XPO1+Epithelial and fibroblasts, monocytes/macrophages, and T/NK cells, along with their associated ligand receptors within the TCGA-LIHC cohort (n=374). **(G)** ST-seq was used to assess the spatial distribution and correlation between XPO1+Epithelial, fibroblasts, monocytes/macrophages, T/NK cells, and their interacting ligand receptors in HCC.

To further explore the crosstalk between XPO1+Epithelial and the TME in HCC, we performed intercellular communication analysis via ‘CellChat’. The results indicated that among the four epithelial cell clusters, XPO1+Epithelial exhibited the highest degree of communication with TME components (**Figure 7A**). XPO1+Epithelial was regulated primarily by fibroblasts (**Figure 7B**). Additionally, signals sent by XPO1+Epithelial predominantly regulate monocyte/macrophages, endothelial cells, and T/NK cells (**Figure 7C**). Our further analysis of key ligand-receptor pairs that interact with XPO1+Epithelial (**Figures 7D, E**) revealed that fibroblasts regulate XPO1+Epithelial mainly through ligand-receptor pairs such as CD99-CD99 and FN1-(ITGAV+ITGB1) (**Figure 7D**). Conversely, XPO1+Epithelial promotes monocyte/macrophage recruitment by regulating monocytes through MIF-(CD74+CXCR4) and MIF-(CD74+CD44), as well as C3-(ITGAX+ITGB2). Furthermore, the iso-ligand receptors MIF-(CD74+CXCR4) and MIF-(CD74+CD44) regulate T/NK cells (**Figure 7E**), potentially facilitating tumor cell evasion of immune surveillance (47, 48). Additionally, XPO1+Epithelial regulates endothelial cells via VEGFA-VEGFR1R2 and VEGFA-VEGFR1, which may be associated with promoting tumor vascular production (49, 50).

To confirm the results observed in the cell-cell communication analyses, we validated our findings via the TCGA-LIHC cohort at the bulk RNA-seq level. These data corroborated the positive correlation between the expression levels of specific receptors in the target cells (**Figure 7F**; **Supplementary Figure S4A**). Further evidence was provided by spatial transcriptome analysis (**Figure 7G**; **Supplementary Figure S4B**), which consistently demonstrated that fibroblasts co-localized with XPO1+Epithelial in specific physical locations. Additionally, the scores for both cell types were positively correlated, and fibroblasts regulated the co-localization of the primary ligand receptor for XPO1+Epithelial cells. Similarly, we observed physical positional co-localization between XPO1+Epithelial and monocytes/macrophages, T/NK cells, endothelial cells, and their corresponding ligands and receptors, suggesting communication exchanges among these cell types. Thus, we validated the interaction network between XPO1+Epithelial cells and the TME via multi-omics.

In summary, our findings indicate that XPO1+Epithelial cells are key components in the remodeling of the TME, and are regulated primarily by ligand signaling from fibroblasts. This interaction may modulate endothelial cells, monocyte macrophages, and T-cells through seeded ligand receptors, potentially influencing immune cell recruitment, immunosuppression, and pro-angiogenesis.

### 3.6 Analysis and mechanistic exploration of the correlation between TP-TME risk subtypes and drug efficacy

**Figure 8:**
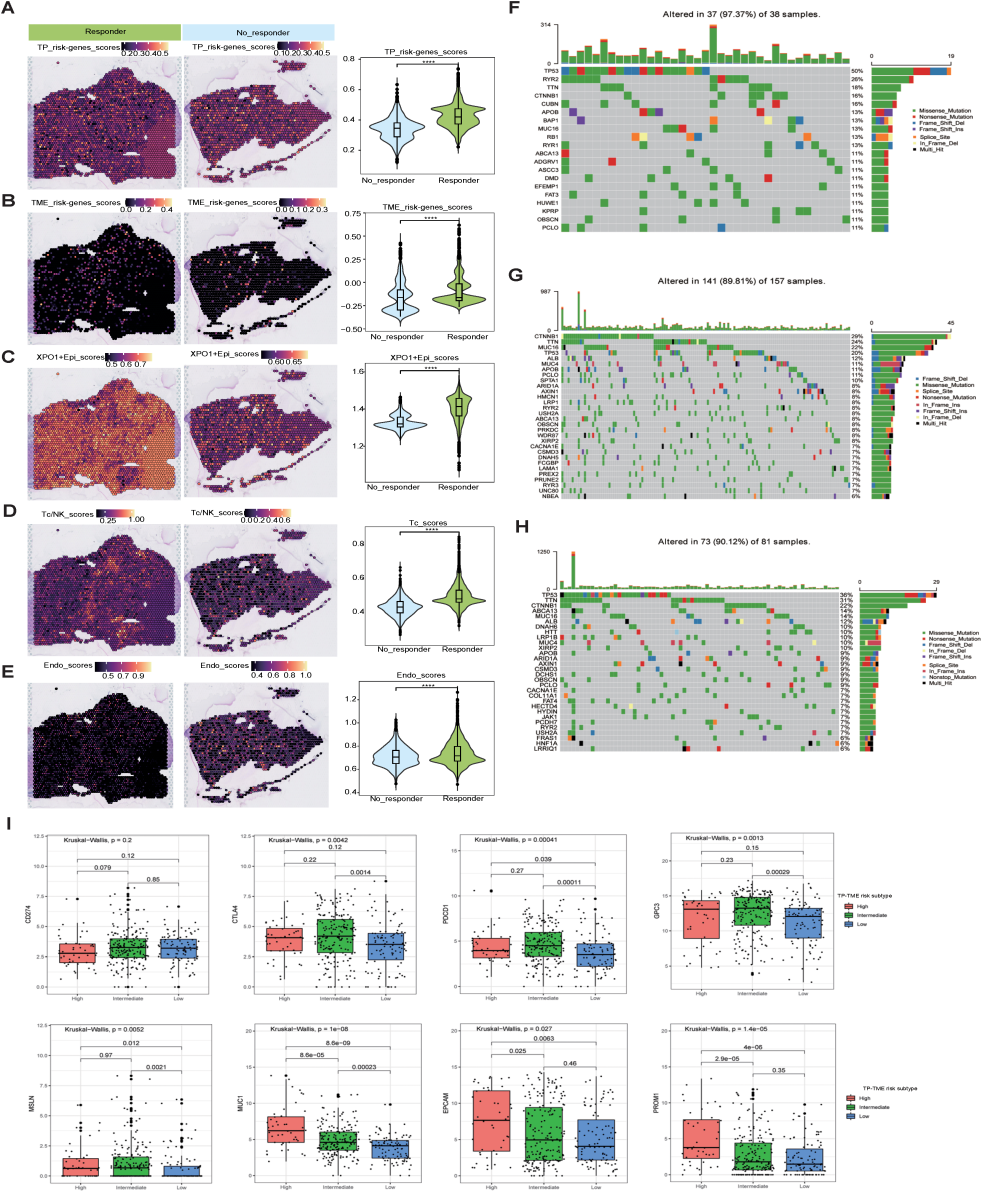
Mutation, stemness, and immunotherapeutic efficacy analysis. **(A–E)** Spatial transcriptomics data (GSE238264) reveal genes associated with TP risk profiles **(A)**, genes linked to TME risk profiles **(B)**, the spatial distribution of XPO1+Epi **(C)**, T/NK cells **(D)**, and endothelial cells **(E)**, and their statistical quantification in patients who either responded or did not respond to TKIs in combination with PD1 therapy. The Wilcoxon test was employed to evaluate differences between groups, with significance levels indicated as follows: ‘****’, corresponding to P<0.0001, respectively. **(F–H)** Violin plots illustrating the top 30 mutated genes across three risk subtypes: **(F)** TP-TME high-risk subtype, **(G)** TP-TME intermediate-risk subtype, and **(H)** TP-TME low-risk subtype. **(I)** The expression levels of CD274, CTLA4, PDCD1, GPC3, MSLN, MUC1, EPCAM, and PROM1 are presented across the three TP-TME risk subtypes.

In light of the analyses conducted at both the scRNA-seq and ST-seq levels, we determined that XPO1+Epithelial in HCC significant interacts with endothelial cells and T cells. This observation led us to speculate that XPO1+Epithelial may serve as a potential target for anti-angiogenic therapies and immunotherapy. To further investigate this hypothesis, we validated our findings via spatial transcriptome samples from the HCC treatment cohort. Compared with the nonresponsive group, the group that responded to the combination of TKIs and PD-1 treatment presented significantly increase signature gene scores (**Figures 8A, B**) for TP-TME risk subtypes, as well as elevated scores for XPO1+Epithelial (**Figure 8C**). Additionally, T-cell and endothelial cell infiltration was notably more pronounced in the combination treatment response group (**Figures 8D, E**).

Through PATHOME-Drug analysis, we constructed drug-target networks to identify potentially effective drugs for the high-risk subtypes (**Supplementary Figure S5A**). The identified drugs included recommended agents, such as sorafenib, regorafenib, cabozantinib, and pembrolizumab (**Supplementary Table S6**). Collectively, these results suggest that TP-TME risk subtypes may be used to predict the efficacy of targeted and immunological therapies, warranting further investigation in follow-up cohort studies.

We conducted a preliminary exploration of the potential mechanisms associated with the genes that characterize the TP-TME risk subtypes and their implications for immunotherapy efficacy. Analysis of the top 30 mutated genes (**Figures 8F–H**) across the high-, intermediate-, and low-risk subtypes revealed that the genes with the highest mutation percentages in the different risk groups included TP53, TTN, and CTNNB1. Notably, the mutation percentages are greater in the intermediate- and high-risk groups, and these mutations are closely linked to the onset and progression of various tumors. There are currently few known drugs that target these mutated genes. Additionally, we observed that the tumor mutational burden (TMB) was low in HCC patients and did not differ significantly among the three subtypes (**Supplementary Figure S5B**), indicating that TMB may not serve as a valid biomarker for selecting HCC patients for treatment with immune checkpoint inhibitors (ICIs). Furthermore, our analyses revealed no significant differences in the stemness scores among the three risk subtypes (**Supplementary Figure S5C**). Although no significant differences were observed in the expression of CD274 (also known as PDL1) among the three risk subtypes, the expression levels of CTLA4 and PDCD1 were significantly increase in the intermediate- and high-risk subtypes than in compared to the low-risk subtype (**Figure 8I**). Consequently, the response rates of the intermediate- and high-risk subtypes to ICIs treatment may be greater than that those of the low-risk subtype. Furthermore, the three risk subtypes of the TP-TME presented distinct expression levels of the 5 cancer antigens targeted in chimeric antigen receptor-modified T cell (CAR-T) therapy (**Figure 8I**). Specifically, GPC3 expression was elevated in the intermediate-risk subtype relative to the low-risk subtype, whereas MSLN expression was higher in both the intermediate- and high-risk subtypes than in the low-risk subtype. The highest expression levels of MUC1, EPCAM, and PROM1 were observed in the high-risk subtype. Therefore, the three TP-TME risk subtypes may exhibit varying therapeutic responses to the corresponding CAR-T therapies.

## 4 Discussion

The heterogeneity of HCC is attributed to various etiologies, such as vial or parasitic infections, chemical carcinogens, cigarette smoking, excess alcohol intake, and dietary factors (51, 77). One of the essential efforts for improving the poor outcome of HCC is to provide a subtyping system that is capable of accurately defining tumor risk subtypes, each displaying unique molecular characteristics linked to potentially druggable driver genes, in order to provide personalized treatment choices on basis of the subtyping system. Although many efforts, which have focused mainly on malignant cells, have focused on intertumor heterogeneity and proposed various single- or multi-omics-based molecular typing systems (52, 53), their effectiveness for providing precision treatment remains limited. Given that the crucial role of the TME in cancers has been confirmed (54), TME-related molecules should contribute to the subtyping of HCC. Another challenge of previous molecular typing methods is cost effectiveness, because hundreds of genes or even multiple omics data types are needed.

In the present study, we first identified the genes of related to TP and the TME and subsequently generated a TP-related PRS and a TME-related PRS according to the expression patterns of these types of genes, and further proposed a novel risk subtyping method that could successfully divide patients with HCC into three risk subtypes. Similar to other molecular typing systems (55–58), our subtypes have distinct prognoses and were validated in two independent external datasets.

Unsurprisingly, some of these candidates eleven TP-related PRS genes and seven TME-related genes have been associated with HCC or other types of cancers in previous studies. ESM1 was identified as a biomarker of macrotrabecular-massive HCC (59). HEY1 plays a critical role in the hypoxia-related regulation of mitochondrial activity in HCC (60). The interactions between CTSC and the TNF-α/p38 MAPK signaling pathway are associated with proliferation and metastasis in HCC (61). LANCL1 was reported to protect prostate cancer cells from oxidative stress (62). XPO1 not only regulates tumor proliferation but also enhances sorafenib resistance by promoting EMT (63, 64). It has been identified as a therapeutic target for HCC (65). RCN2 promotes HCC progression by activating the MYC signaling pathway and regulating the EGFR-ERK pathway. In this context, our proteomic analysis revealed that the protein levels of several tumor progression-related PRS genes were elevated in cancer tissues from patients with HCC.

Our research revealed indicates that the genes characterizing the TP risk subtypes, such as XPO1 and RCN2, in HCC have not yet been examined at the single-cell level. In this context, we investigated the critical functions of genes defining TP risk subtypes in HCC at single-cell resolution. We identified a previously unreported malignant cell cluster, XPO1+Epithelial, exhibiting features associated with the TP-TME risk subtype. This cluster involves the upregulation of the TFs MEF2A, TCF7L2, and ZNF148, which significant activate the TGF-beta signaling pathway, and the WNT/beta -catenin signaling pathway, and promote EMT, all of which play a crucial pro-oncogenic roles (45, 46, 66–68). Furthermore, these factors are associated with elevated tumorigenic characteristics such as proliferation and migration. Collectively, these findings suggest that the XPO1+Epithelial, with TP-TME risk subtype-related features, can serve as a predictor of tumor malignancy.

There is a consensus regarding the significant impact of the TME on various tumor phenotypes. Accordingly, we further analyzed the cell-cell communication between XPO1+ Epithelial and various components of the TME. Our findings indicate that fibroblasts are the predominant cell type regulating XPO1+ Epithelial, primarily through enhanced ligand-receptor interactions, such as those involving FN1-(ITGAV+ITGB1) and CD99-CD99. These interactions, which are consistent with previous reports, correlated with increased up-regulation of tumor EMT through ligand-receptor signaling. Furthermore, XPO1+ Epithelial can modulate monocyte macrophages, T cells, and endothelial cells through multiple ligand-receptor pairs. For example, XPO1+ Epithelial can interact with immune cells via several ligand-receptor pairs, such as MIF-(CD74+CXCR4) and MIF-(CD74+CD44), which, as previously reported, function as recruiters of immunosuppressive cells and thus promote immunosuppression, enabling tumor cells to evade immune surveillance (47, 48, 69). Additionally, XPO1+ Epithelial can promote angiogenesis via VEGFA-VEGFR1/R2, thereby facilitating tumor growth, which aligns with our previous study (19). We fully validated these findings through multi-omics, utilizing both the TCGA-LIHC cohort and the paired ST-seq cohort.

By analyzing ST-seq data from HCC patients treated with TKIs in combination with PD-1 inhibitors, we observed that the scores of TP, TME-RPS-related genes, and XPO1+Epithelial genes were significantly increase in the responsive group. Furthermore, endothelial cell and T-cell infiltration were significantly increase in the responsive group than in the nonresponsive group. This strongly suggests that the TP-TME high-risk subtype may exhibit greater sensitivity to TKIs combined with PD-1 therapy; however, this finding requires validation through further studies. Additionally, while further research is necessary, we propose potential immunotherapies and drugs for high-risk subtypes, which may aid in clinical decision-making.

Mutations in several key genes play crucial roles in tumorigenesis (70). Consistent with previous studies, both TP53 and CTNNB1 presented high mutation probabilities across different risk groups (71). Research has demonstrated that HCC with CTNNB1 mutations tends to be well differentiated and associated with a better prognosis. In contrast, HCC with TP53 mutations, particularly in the absence of CTNNB1 mutations, is more aggressive and strongly linked to poor outcomes (72). Our findings indicate that a higher mutation probability of TP53, coupled with a lower mutation probability of CTNNB1, is prevalent in high-risk groups, strongly suggesting that HCC classified within the TP-TME high-risk subtype is more aggressive.

Furthermore, we investigated the sensitivity of various TP-TME risk subtypes to immunotherapy. Our comparative analysis of immune checkpoint expression across different TP-TME risk subtypes revealed elevated levels of immune checkpoints such as PDCD1 and CTLA4, in intermediate- and high-risk subtypes. These findings suggest that these risk subtypes may respond more effectively to immune checkpoint inhibitors (73). Additionally, the expression levels of antigens used in CAR-T therapy vary among the different risk subtypes, indicating that the three TP-TME risk subtypes may exhibit distinct responses to CAR-T therapy.

Owing to the low sensitivity of conventional diagnostic techniques and the lack of pronounced early symptoms, HCC is often diagnosed at an advanced stage (2, 78). Despite recent advancements in HCC treatment, many patients still experience treatment resistance and disease progression (74, 79). Our proposed classification method aims to improve prognosis evaluation in HCC and identify patients likely to benefit from TKIs and PD-1 inhibitors. By leveraging a risk score and the proportion of XPO1+Epithelial expression, clinicians can predict patient responses to TKIs and PD-1 therapy, facilitating the development of personalized immunotherapy regimens designed to improve patient outcomes.

Although our current study introduces a novel molecular classification system and elaborates on the biological and clinical significance of the XPO1+Epithelial, several noteworthy limitations exist. First, the TP-TME risk model was derived from retrospective analyses and needs to be validated and optimized in future prospective trials to ensure its applicability and accuracy in different populations and settings. Second, our sample of included scRNA-seq data was limited; despite the use of ST-seq and bulk RNA-seq for validation, the findings must still be validated in a larger cohort. For future research directions, in addition to validating the risk subtype model, further investigations of subtype-specific responses to immunotherapy are crucial. Future studies could initiate exploratory clinical trials tailored to the characteristics of TP-TME high-risk subtypes, allowing for the assessment of their responses to existing or novel immunotherapies, thereby supporting individualized treatment strategies. Specifically, targeting particular molecular markers that may be present in XPO1+ Epithelial cells could facilitate the further development and optimization of targeted drugs, ultimately enhancing therapeutic efficacy while minimizing toxic side effects.

## 5 Conclusion

We proposed and validated a novel risk subtype system for HCC that is based on tumor progression in the TP-TME. Additionally, we identified and validated the biological behavior and clinical significance of XPO1+Epithelial, a novel category among the TP risk subtypes characterized, across multiple cohorts. These findings enhance prognostic risk prediction for HCC patients and provide valuable insights for predicting personalized targeted therapy and immunotherapy.

## Data Availability

The original contributions presented in the study are included in the article/**Supplementary Material**. Further inquiries can be directed to the corresponding authors.

## Supplementary material

Supplementary Figure 1 Protein-protein interaction networks of the TP-related genes and TME-related genes. Red represents upregulated, and blue represents downregulated genes in hepatocellular carcinoma. The circular nodes represent TP-related genes, and the diamond nodes represent TME-related genes.

Supplementary Figure 2 Validation of TP-TME risk subtypes in multiple cohorts. **(A–E)** Validation of TP-TME risk subtypes in the TCGA-LIHC. **(A)** HCC with high TP-related PRS had shorter overall survival than those with low TP-related PRS. **(B)** HCC with high TME-related PRS had shorter overall survival than those with low TME-related PRS. **(C)** There were significant differences in overall survival among the three subtypes of the TP-TME risk subtypes. **(D)** There were significant differences in progression-free survival among the three subtypes of the TP-TME risk subtypes. **(E)** The TP-TME risk subtype system was proven to be an independent prognostic factor, after adjusting for other clinicopathological characteristics. **(F–I)** Validation of TP-TME risk subtypes in LIRI-JP. **(F)** HCC with high TP-related PRS had shorter overall survival than those with low TP-related PRS. **(G)** HCC with high TME-related PRS had shorter overall survival than those with low TME-related PRS. **(H)** There were significant differences in overall survival among the three subtypes in the TP-TME risk subtypes. **(I)** The TP-TME risk subtype system was proved to be an independent prognostic factor, after adjusting for other clinicopathological characteristics.

Supplementary Figure 3 Illustrates the functional scoring of epithelial cells. **(A)** Hierarchical heatmap displaying large-scale copy number variations (CNVs) in epithelial cells. **(B)** Bubble plots depict the average expression and percentage of expression of the TME risk subtype signature genes across various cell types.

Supplementary Figure 4 XPO1+Epi and endothelial cell interactions in the TCGA-LIHC cohort and spatial transcriptome cohort correlations. **(A)** A scatterplot illustrating the correlation between XPO1+Epithelial and endothelial cells, along with their ligand receptors, in the TCGA-LIHC cohort (n=374). **(B)** ST-seq analysis revealing the spatial distribution and correlation between endothelial cells and ligand receptors of XPO1+Epithelial interacting with endothelial cells in HCC.

Supplementary Figure 5 Drug-target networks for potentially effective drugs for the TP-TME high-risk subtypes. **(A)** Drug-target networks for potentially effective drugs for the TP-TME high-risk subtypes. Red represents upregulated, and blue represents downregulated in the TP-TME high-risk subtypes. The Wilcox test was used to assess the differences between groups. **(B)** Tumor mutation burden scores of the three TP-TME risk subtypes. **(C)** Stemness scores of the three TP-TME risk subtypes.

## Glossary

HCC: Hepatocellular carcinoma
scRNA-seq: Single-cell RNA sequencing
ST-seq: Spatial transcriptomics sequencing
TP: Tumor purity
TME: Tumor microenvironment
EMT: Epithelial-mesenchymal transition
TKIs: Tyrosine Kinase Inhibitors
PD-1: Programmed Cell Death Protein 1
IHC: Immunohistochemical
TCGA: The Cancer Genome Atlas Program
DEGs: Differentially expressed genes
HR: Hazard Ratio
AUC: Area under curve
KEGG: The Kyoto Encyclopedia of Genes and Genomes
MSigDB: The Molecular Signatures Database
GO: The Gene Ontology
PPI: Protein-protein interaction
PRS: Polygenic risk score
OS: Overall survival
PFS: Progression free survival
LASSO: The least absolute shrinkage and selection operator
CNV: Chromosome copy number variation
TFs: Transcription factors
MAF: Mutation annotation format
TMB: Tumor mutational burden
tROC: Time-dependent receiver operating characteristic curve
L-R: Ligand-receptor
Epi: Epithelial cell
Fib: Fibrobalst cell
Endo: Endothlial cell
Tc/Nk: T cell/Natural killer cell
Bc: B cell
Mono/Mac: Monocytes/Macrophages cell
Mast: Mast cell
Neu: Neutrophil cell
Cycling: Cycling cell

## References

[1] Siegel RL Giaquinto AN Jemal A. Cancer statistics, 2024. CA Cancer J Clin. (2024) 74:12–49. doi: 10.3322/caac.21820 38230766

[2] Forner A Reig M Bruix J. Hepatocellular carcinoma. Lancet. (2018) 391:1301–14. doi: 10.1016/S0140-6736(18)30010-2 29307467

[3] Caruso S Calatayud AL Pilet J La Bella T Rekik S Imbeaud S. Analysis of liver cancer cell lines identifies agents with likely efficacy against hepatocellular carcinoma and markers of response. Gastroenterology. (2019) 157:760–76. doi: 10.1053/j.gastro.2019.05.001 31063779

[4] de Bono JS Ashworth A. Translating cancer research into targeted therapeutics. Nature. (2010) 467:543–9. doi: 10.1038/nature09339 20882008

[5] Losic B Craig AJ Villacorta-Martin C Martins-Filho SN Akers N Chen X. Intratumoral heterogeneity and clonal evolution in liver cancer. Nat Commun. (2020) 11:291. doi: 10.1038/s41467-019-14050-z 31941899 PMC6962317

[6] Minagawa M Ikai I Matsuyama Y Yamaoka Y Makuuchi M. Staging of hepatocellular carcinoma: assessment of the Japanese TNM and AJCC/UICC TNM systems in a cohort of 13,772 patients in Japan. Ann Surg. (2007) 245:909–22. doi: 10.1097/01.sla.0000254368.65878.da PMC1876960 17522517

[7] Nault JC Martin Y Caruso S Hirsch TZ Bayard Q Calderaro J. Clinical impact of genomic diversity from early to advanced hepatocellular carcinoma. Hepatology. (2020) 71:164–82. doi: 10.1002/hep.30811 31206197

[8] Joyce JA Pollard JW. Microenvironmental regulation of metastasis. Nat Rev Cancer. (2009) 9:239–52. doi: 10.1038/nrc2618 PMC3251309 19279573

[9] Zhang C Cheng W Ren X Wang Z Liu X Li G. Tumor purity as an underlying key factor in glioma. Clin Cancer Res. (2017) 23:6279–91. doi: 10.1158/1078-0432.CCR-16-2598 28754819

[10] Aran D Sirota M Butte AJ. Systematic pan-cancer analysis of tumour purity. Nat Commun. (2015) 6:8971. doi: 10.1038/ncomms9971 26634437 PMC4671203

[11] Rhee JK Jung YC Kim KR Yoo J Kim J Lee YJ. Impact of tumor purity on immune gene expression and clustering analyses across multiple cancer types. Cancer Immunol Res. (2018) 6:87–97. doi: 10.1158/2326-6066.CIR-17-0201 29141981

[12] Hanahan D Coussens LM. Accessories to the crime: functions of cells recruited to the tumor microenvironment. Cancer Cell. (2012) 21:309–22. doi: 10.1016/j.ccr.2012.02.022 22439926

[13] Pitt JM Marabelle A Eggermont A Soria JC Kroemer G Zitvogel L. Targeting the tumor microenvironment: removing obstruction to anticancer immune responses and immunotherapy. Ann Oncol. (2016) 27:1482–92. doi: 10.1093/annonc/mdw168 27069014

[14] Mayakonda A Lin DC Assenov Y Plass C Koeffler HP. Maftools: efficient and comprehensive analysis of somatic variants in cancer. Genome Res. (2018) 28:1747–56. doi: 10.1101/gr.239244.118 PMC6211645 30341162

[15] Miao YR Zhang Q Lei Q Luo M Xie GY Wang H. ImmuCellAI: A unique method for comprehensive T-cell subsets abundance prediction and its application in cancer immunotherapy. Adv Sci (Weinh). (2020) 7:1902880. doi: 10.1002/advs.201902880 32274301 PMC7141005

[16] Lu J Chen Y Zhang X Guo J Xu K Li L. A novel prognostic model based on single-cell RNA sequencing data for hepatocellular carcinoma. Cancer Cell Int. (2022) 22:38. doi: 10.1186/s12935-022-02469-2 35078458 PMC8787928

[17] Gulati GS D'Silva JP Liu Y Wang L Newman AM. Profiling cell identity and tissue architecture with single-cell and spatial transcriptomics. Nat Rev Mol Cell Biol. (2024). doi: 10.1038/s41580-024-00768-2 39169166

[18] Bressan D Battistoni G Hannon GJ. The dawn of spatial omics. Science. (2023) 381:eabq4964. doi: 10.1126/science.abq4964 37535749 PMC7614974

[19] Ye J Gao X Huang X Huang S Zeng D Luo W. Integrating single-cell and spatial transcriptomics to uncover and elucidate GP73-mediated pro-angiogenic regulatory networks in hepatocellular carcinoma. Res (Wash D C). (2024) 7:0387. doi: 10.34133/research.0387 PMC11208919 38939041

[20] Lian Q Wang S Zhang G Wang D Luo G Tang J. HCCDB: A database of hepatocellular carcinoma expression atlas. Genomics Proteomics Bioinf. (2018) 16:269–75. doi: 10.1016/j.gpb.2018.07.003 PMC6205074 30266410

[21] Roessler S Jia HL Budhu A Forgues M Ye QH Lee JS. A unique metastasis gene signature enables prediction of tumor relapse in early-stage hepatocellular carcinoma patients. Cancer Res. (2010) 70:10202–12. doi: 10.1158/0008-5472.CAN-10-2607 PMC3064515 21159642

[22] Gao Q Zhu H Dong L Shi W Chen R Song Z. Integrated proteogenomic characterization of HBV-related hepatocellular carcinoma. Cell. (2019) 179:561–77.e22. doi: 10.1016/j.cell.2019.10.038 31585088

[23] Zhang S Yuan L Danilova L Mo G Zhu Q Deshpande A. Spatial transcriptomics analysis of neoadjuvant cabozantinib and nivolumab in advanced hepatocellular carcinoma identifies independent mechanisms of resistance and recurrence. Genome Med. (2023) 15:72. doi: 10.1186/s13073-023-01218-y 37723590 PMC10506285

[24] Yoshihara K Shahmoradgoli M Martínez E Vegesna R Kim H Torres-Garcia W. Inferring tumour purity and stromal and immune cell admixture from expression data. Nat Commun. (2013) 4:2612. doi: 10.1038/ncomms3612 24113773 PMC3826632

[25] Aran D Hu Z Butte AJ. xCell: digitally portraying the tissue cellular heterogeneity landscape. Genome Biol. (2017) 18:220. doi: 10.1186/s13059-017-1349-1 29141660 PMC5688663

[26] Ritchie ME Phipson B Wu D Hu Y Law CW Shi W. limma powers differential expression analyses for RNA-sequencing and microarray studies. Nucleic Acids Res. (2015) 43:e47. doi: 10.1093/nar/gkv007 25605792 PMC4402510

[27] Mootha VK Lindgren CM Eriksson KF Subramanian A Sihag S Lehar J. PGC-1alpha-responsive genes involved in oxidative phosphorylation are coordinately downregulated in human diabetes. Nat Genet. (2003) 34:267–73. doi: 10.1038/ng1180 12808457

[28] Subramanian A Tamayo P Mootha VK Mukherjee S Ebert BL Gillette MA. Gene set enrichment analysis: a knowledge-based approach for interpreting genome-wide expression profiles. Proc Natl Acad Sci U S A. (2005) 102:15545–50. doi: 10.1073/pnas.0506580102 PMC1239896 16199517

[29] Liberzon A Subramanian A Pinchback R Thorvaldsdóttir H Tamayo P Mesirov JP. Molecular signatures database (MSigDB) 3.0. Bioinformatics. (2011) 27:1739–40. doi: 10.1093/bioinformatics/btr260 PMC3106198 21546393

[30] Wu T Hu E Xu S Chen M Guo P Dai Z. clusterProfiler 4.0: A universal enrichment tool for interpreting omics data. Innovation (Camb). (2021) 2:100141. doi: 10.1016/j.xinn.2021.100141 34557778 PMC8454663

[31] Szklarczyk D Gable AL Lyon D Junge A Wyder S Huerta-Cepas J. STRING v11: protein-protein association networks with increased coverage, supporting functional discovery in genome-wide experimental datasets. Nucleic Acids Res. (2019) 47:D607–d13. doi: 10.1093/nar/gky1131 PMC6323986 30476243

[32] Shannon P Markiel A Ozier O Baliga NS Wang JT Ramage D. Cytoscape: a software environment for integrated models of biomolecular interaction networks. Genome Res. (2003) 13:2498–504. doi: 10.1101/gr.1239303 PMC403769 14597658

[33] Friedman J Hastie T Tibshirani R. Regularization paths for generalized linear models via coordinate descent. J Stat Softw. (2010) 33:1–22. doi: 10.18637/jss.v033.i01 20808728 PMC2929880

[34] Hao Y Hao S Andersen-Nissen E Mauck WM 3rd Zheng S Butler A. Integrated analysis of multimodal single-cell data. Cell. (2021) 184:3573–87.e29. doi: 10.1016/j.cell.2021.04.048 34062119 PMC8238499

[35] McGinnis CS Murrow LM Gartner ZJ. DoubletFinder: doublet detection in single-cell RNA sequencing data using artificial nearest neighbors. Cell Syst. (2019) 8:329–37.e4. doi: 10.1016/j.cels.2019.03.003 30954475 PMC6853612

[36] Korsunsky I Millard N Fan J Slowikowski K Zhang F Wei K. Fast, sensitive and accurate integration of single-cell data with Harmony. Nat Methods. (2019) 16:1289–96. doi: 10.1038/s41592-019-0619-0 PMC6884693 31740819

[37] Van de Sande B Flerin C Davie K De Waegeneer M Hulselmans G Aibar S. A scalable SCENIC workflow for single-cell gene regulatory network analysis. Nat Protoc. (2020) 15:2247–76. doi: 10.1038/s41596-020-0336-2 32561888

[38] Jin S Guerrero-Juarez CF Zhang L Chang I Ramos R Kuan CH. Inference and analysis of cell-cell communication using CellChat. Nat Commun. (2021) 12:1088. doi: 10.1038/s41467-021-21246-9 33597522 PMC7889871

[39] Hänzelmann S Castelo R Guinney J. GSVA: gene set variation analysis for microarray and RNA-seq data. BMC Bioinf. (2013) 14:7. doi: 10.1186/1471-2105-14-7 PMC3618321 23323831

[40] Colaprico A Silva TC Olsen C Garofano L Cava C Garolini D. TCGAbiolinks: an R/Bioconductor package for integrative analysis of TCGA data. Nucleic Acids Res. (2016) 44:e71. doi: 10.1093/nar/gkv1507 26704973 PMC4856967

[41] Thorsson V Gibbs DL Brown SD Wolf D Bortone DS Ou Yang TH. The immune landscape of cancer. Immunity. (2018) 48:812–30.e14. doi: 10.1016/j.immuni.2018.03.023 29628290 PMC5982584

[42] Malta TM Sokolov A Gentles AJ Burzykowski T Poisson L Weinstein JN. Machine learning identifies stemness features associated with oncogenic dedifferentiation. Cell. (2018) 173:338–54.e15. doi: 10.1016/j.cell.2018.03.034 29625051 PMC5902191

[43] Ma S Li X Wang X Cheng L Li Z Zhang C. Current progress in CAR-T cell therapy for solid tumors. Int J Biol Sci. (2019) 15:2548–60. doi: 10.7150/ijbs.34213 PMC6854376 31754328

[44] Blanche P Dartigues JF Jacqmin-Gadda H. Estimating and comparing time-dependent areas under receiver operating characteristic curves for censored event times with competing risks. Stat Med. (2013) 32:5381–97. doi: 10.1002/sim.v32.30 24027076

[45] Xiao Q Gan Y Li Y Fan L Liu J Lu P. MEF2A transcriptionally upregulates the expression of ZEB2 and CTNNB1 in colorectal cancer to promote tumor progression. Oncogene. (2021) 40:3364–77. doi: 10.1038/s41388-021-01774-w PMC8116210 33863999

[46] Ravindranath AJ Cadigan KM. The role of the C-clamp in wnt-related colorectal cancers. Cancers (Basel). (2016) 8(8):74. doi: 10.3390/cancers8080074 27527215 PMC4999783

[47] Schwartz V Lue H Kraemer S Korbiel J Krohn R Ohl K. A functional heteromeric MIF receptor formed by CD74 and CXCR4. FEBS Lett. (2009) 583:2749–57. doi: 10.1016/j.febslet.2009.07.058 PMC2911026 19665027

[48] Borghese F Clanchy FI. CD74: an emerging opportunity as a therapeutic target in cancer and autoimmune disease. Expert Opin Ther Targets. (2011) 15:237–51. doi: 10.1517/14728222.2011.550879 21208136

[49] Shibuya M. Vascular endothelial growth factor receptor-1 (VEGFR-1/Flt-1): a dual regulator for angiogenesis. Angiogenesis. (2006) 9:225–30; discussion 31. doi: 10.1007/s10456-006-9055-8 17109193

[50] Mabeta P Steenkamp V. The VEGF/VEGFR axis revisited: implications for cancer therapy. Int J Mol Sci. (2022) 23(24):15585. doi: 10.3390/ijms232415585 36555234 PMC9779738

[51] Schulze K Nault JC Villanueva A. Genetic profiling of hepatocellular carcinoma using next-generation sequencing. J Hepatol. (2016) 65:1031–42. doi: 10.1016/j.jhep.2016.05.035 27262756

[52] Wu Y Liu Z Xu X. Molecular subtyping of hepatocellular carcinoma: A step toward precision medicine. Cancer Commun (Lond). (2020) 40:681–93. doi: 10.1002/cac2.v40.12 PMC7743018 33290597

[53] Lu LC Hsu CH Hsu C Cheng AL. Tumor heterogeneity in hepatocellular carcinoma: facing the challenges. Liver Cancer. (2016) 5:128–38. doi: 10.1159/000367754 PMC4906428 27386431

[54] Hinshaw DC Shevde LA. The tumor microenvironment innately modulates cancer progression. Cancer Res. (2019) 79:4557–66. doi: 10.1158/0008-5472.CAN-18-3962 PMC6744958 31350295

[55] Cancer Genome Atlas Research Network Ley TJ Miller C Ding L Raphael BJ Mungall AJ. Comprehensive and integrative genomic characterization of hepatocellular carcinoma. Cell. (2017) 169:1327–41.e23. doi: 10.1016/j.cell.2017.05.046 28622513 PMC5680778

[56] Fujimoto A Furuta M Totoki Y Tsunoda T Kato M Shiraishi Y. Whole-genome mutational landscape and characterization of noncoding and structural mutations in liver cancer. Nat Genet. (2016) 48:500–9. doi: 10.1038/ng.3547 27064257

[57] Lee JS Chu IS Heo J Calvisi DF Sun Z Roskams T. Classification and prediction of survival in hepatocellular carcinoma by gene expression profiling. Hepatology. (2004) 40:667–76. doi: 10.1002/hep.20375 15349906

[58] Jiang Y Sun A Zhao Y Ying W Sun H Yang X. Proteomics identifies new therapeutic targets of early-stage hepatocellular carcinoma. Nature. (2019) 567:257–61. doi: 10.1038/s41586-019-0987-8 30814741

[59] Calderaro J Meunier L Nguyen CT Boubaya M Caruso S Luciani A. ESM1 as a marker of macrotrabecular-massive hepatocellular carcinoma. Clin Cancer Res. (2019) 25:5859–65. doi: 10.1158/1078-0432.CCR-19-0859 31358545

[60] Kung-Chun Chiu D Pui-Wah Tse A Law CT Ming-Jing Xu I Lee D Chen M. Hypoxia regulates the mitochondrial activity of hepatocellular carcinoma cells through HIF/HEY1/PINK1 pathway. Cell Death Dis. (2019) 10:934. doi: 10.1038/s41419-019-2155-3 31819034 PMC6901483

[61] Zhang GP Yue X Li SQ. Cathepsin C interacts with TNF-α/p38 MAPK signaling pathway to promote proliferation and metastasis in hepatocellular carcinoma. Cancer Res Treat. (2020) 52:10–23. doi: 10.4143/crt.2019.145 31048666 PMC6962486

[62] Wang J Xiao Q Chen X Tong S Sun J Lv R. LanCL1 protects prostate cancer cells from oxidative stress via suppression of JNK pathway. Cell Death Dis. (2018) 9:197. doi: 10.1038/s41419-017-0207-0 29416001 PMC5833716

[63] Zheng Y Gery S Sun H Shacham S Kauffman M Koeffler HP. KPT-330 inhibitor of XPO1-mediated nuclear export has anti-proliferative activity in hepatocellular carcinoma. Cancer Chemother Pharmacol. (2014) 74:487–95. doi: 10.1007/s00280-014-2495-8 PMC4146741 25030088

[64] Wang Z Pan B Yao Y Qiu J Zhang X Wu X. XPO1 intensifies sorafenib resistance by stabilizing acetylation of NPM1 and enhancing epithelial-mesenchymal transition in hepatocellular carcinoma. BioMed Pharmacother. (2023) 160:114402. doi: 10.1016/j.biopha.2023.114402 36791564

[65] Azizian NG Li Y. XPO1-dependent nuclear export as a target for cancer therapy. J Hematol Oncol. (2020) 13:61. doi: 10.1186/s13045-020-00903-4 32487143 PMC7268335

[66] Zhang CZ Chen GG Lai PB. Transcription factor ZBP-89 in cancer growth and apoptosis. Biochim Biophys Acta. (2010) 1806:36–41. doi: 10.1016/j.bbcan.2010.03.002 20230874

[67] Wang N Li MY Liu Y Yu J Ren J Zheng Z. ZBP-89 negatively regulates self-renewal of liver cancer stem cells via suppression of Notch1 signaling pathway. Cancer Lett. (2020) 472:70–80. doi: 10.1016/j.canlet.2019.12.026 31874246 PMC7226908

[68] He S Tang S. WNT/β-catenin signaling in the development of liver cancers. BioMed Pharmacother. (2020) 132:110851. doi: 10.1016/j.biopha.2020.110851 33080466

[69] Gore Y Starlets D Maharshak N Becker-Herman S Kaneyuki U Leng L. Macrophage migration inhibitory factor induces B cell survival by activation of a CD74-CD44 receptor complex. J Biol Chem. (2008) 283:2784–92. doi: 10.1074/jbc.M703265200 18056708

[70] Hu HF Ye Z Qin Y Xu XW Yu XJ Zhuo QF. Mutations in key driver genes of pancreatic cancer: molecularly targeted therapies and other clinical implications. Acta Pharmacol Sin. (2021) 42:1725–41. doi: 10.1038/s41401-020-00584-2 PMC8563973 33574569

[71] Zucman-Rossi J Villanueva A Nault JC Llovet JM. Genetic landscape and biomarkers of hepatocellular carcinoma. Gastroenterology. (2015) 149:1226–39.e4. doi: 10.1053/j.gastro.2015.05.061 26099527

[72] Nishida N Nishimura T Kaido T Minaga K Yamao K Kamata K. Molecular scoring of hepatocellular carcinoma for predicting metastatic recurrence and requirements of systemic chemotherapy. Cancers (Basel). (2018) 10(10):367. doi: 10.3390/cancers10100367 30274313 PMC6210853

[73] Shi J Liu J Tu X Li B Tong Z Wang T. Single-cell immune signature for detecting early-stage HCC and early assessing anti-PD-1 immunotherapy efficacy. J Immunother Cancer. (2022) 10(1):e003133. doi: 10.1136/jitc-2021-003133 35101942 PMC8804705

[74] Yang X Yang C Zhang S Geng H Zhu AX Bernards R. Precision treatment in advanced hepatocellular carcinoma. Cancer Cell. (2024) 42:180–97. doi: 10.1016/j.ccell.2024.01.007 38350421

[75] Ye J Lin Y Liao Z Gao X Lu C Lu L. Single cell-spatial transcriptomics and bulk multi-omics analysis of heterogeneity and ecosystems in hepatocellular carcinoma. NPJ Precis Oncol. (2024) 8(1):262. doi: 10.1038/s41698-024-00752-1 39548284 PMC11568154

[76] Ruff SM Pawlik TM. The role of immune checkpoint inhibitors and/or Yttrium-90 radioembolization in the management of hepatocellular carcinoma: challenges of treatment sequence. Hepatoma Res. (2024) 10:33. doi: 10.20517/2394-5079.2024.59

[77] Singh A Anjum B Naz Q Raza S Sinha RA Ahmad MK. Night shift-induced circadian disruption: links to initiation of non-alcoholic fatty liver disease/non-alcoholic steatohepatitis and risk of hepatic cancer. Hepatoma Res. (2024), 2394-5079.2024.88. doi: 10.20517/2394-5079.2024.88 39525867 PMC7616786

[78] Ainiwaer A Chen Y Lu Y. Precise staging of advanced HCC promotes higher quality of personalized treatment management: Chinese experts consensus on precision diagnosis and management of advanced hepatocellular carcinoma (2023). Hepatoma Res. (2024) 10:4. doi: 10.20517/2394-5079.2023.155

[79] Liang R Zhang J Liu Z Liu Z Li Q Luo X. Mechanism and Molecular network of RBM8A-mediated regulation of oxaliplatin resistance in hepatocellular carcinoma. Front Oncol. (2021) 10:585452. doi: 10.3389/fonc.2020.585452 33552961 PMC7862710

