# Supplementary figures and images for: Integrative multi-omics analysis reveals a novel subtype of hepatocellular carcinoma with biological and clinical relevance
Source: Front Immunol. 2024 Dec 6;15:1517312. doi: 10.3389/fimmu.2024.1517312 (PMC11659151; doi:10.3389/fimmu.2024.1517312)

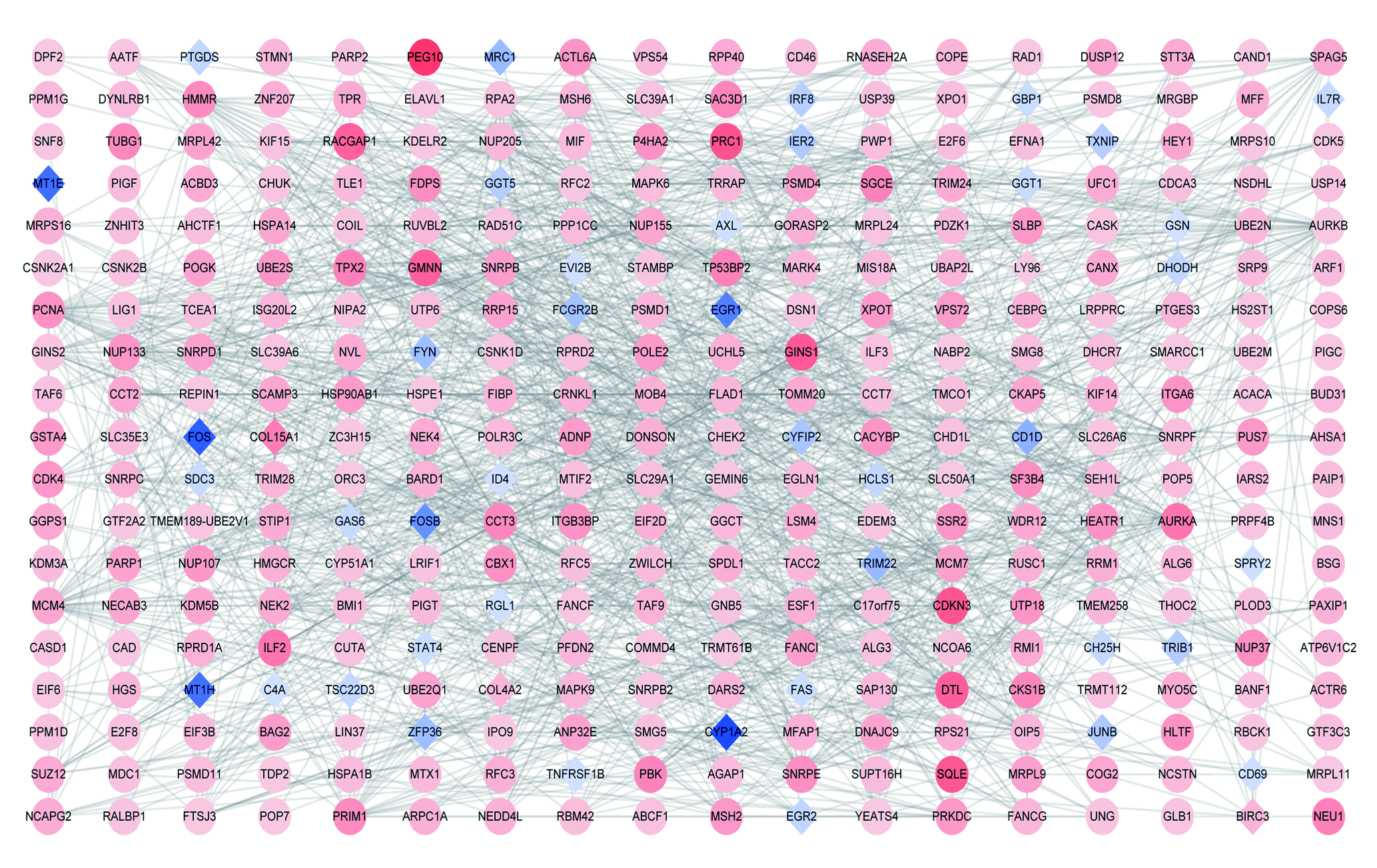

Supplement: Supplementary Figure 1 — Protein-protein interaction networks of the TP-related genes and TME-related genes. Red represents upregulated, and blue represents downregulated genes in hepatocellular carcinoma. The circular nodes represent TP-related genes, and the diamond nodes represent TME-related genes. [file Image1.tiff]

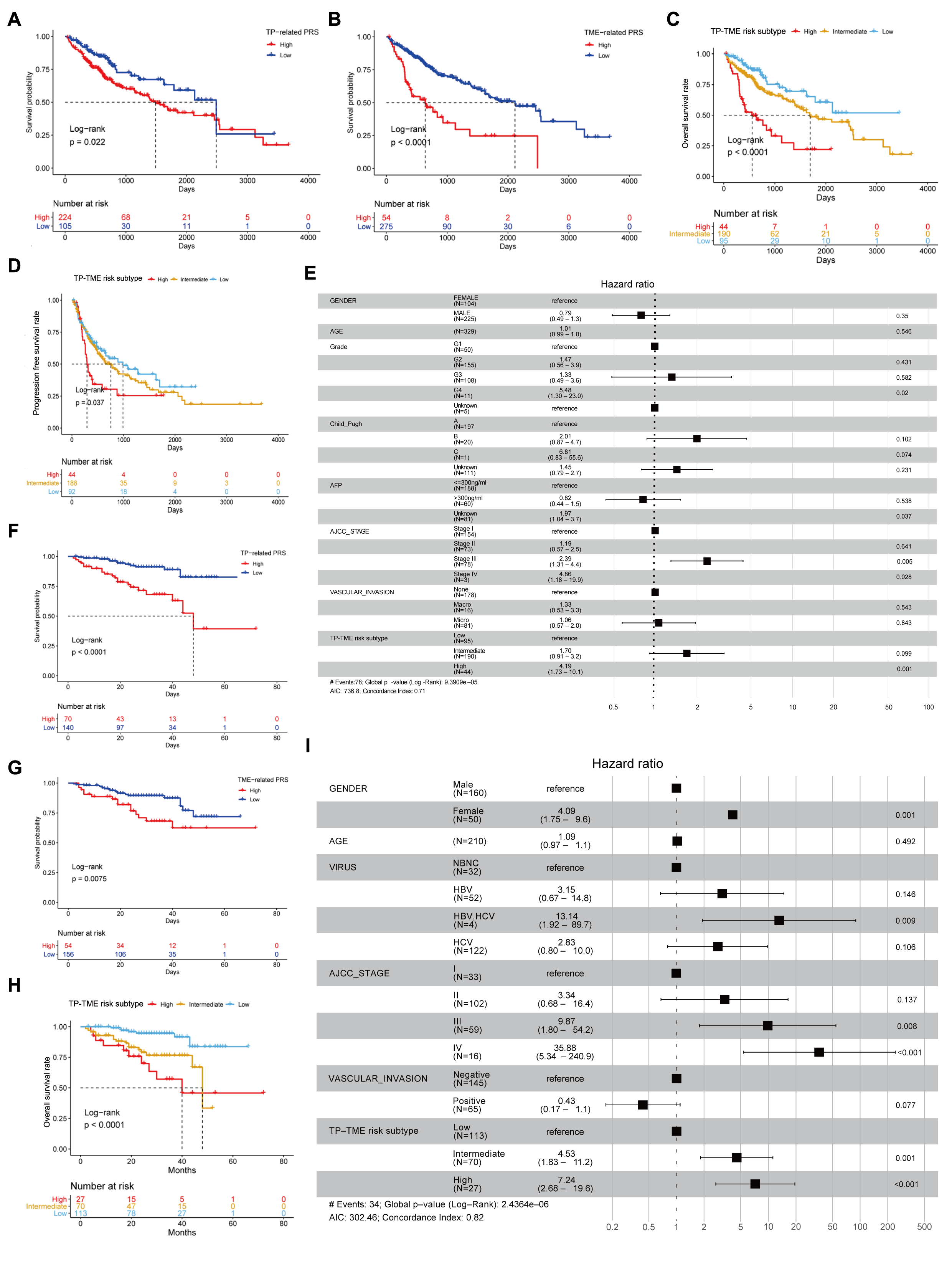

Supplement: Supplementary Figure 2 — Validation of TP-TME risk subtypes in multiple cohorts. (A–E) Validation of TP-TME risk subtypes in the TCGA-LIHC. (A) HCC with high TP-related PRS had shorter overall survival than those with low TP-related PRS. (B) HCC with high TME-related PRS had shorter overall survival than those with low TME-related PRS. (C) There were significant differences in overall survival among the three subtypes of the TP-TME risk subtypes. (D) There were significant differences in progression-free survival among the three subtypes of the TP-TME risk subtypes. (E) The TP-TME risk subtype system was proven to be an independent prognostic factor, after adjusting for other clinicopathological characteristics. (F–I) Validation of TP-TME risk subtypes in LIRI-JP. (F) HCC with high TP-related PRS had shorter overall survival than those with low TP-related PRS. (G) HCC with high TME-related PRS had shorter overall survival than those with low TME-related PRS. (H) There were significant differences in overall survival among the three subtypes in the TP-TME risk subtypes. (I) The TP-TME risk subtype system was proved to be an independent prognostic factor, after adjusting for other clinicopathological characteristics. [file Image2.tif]

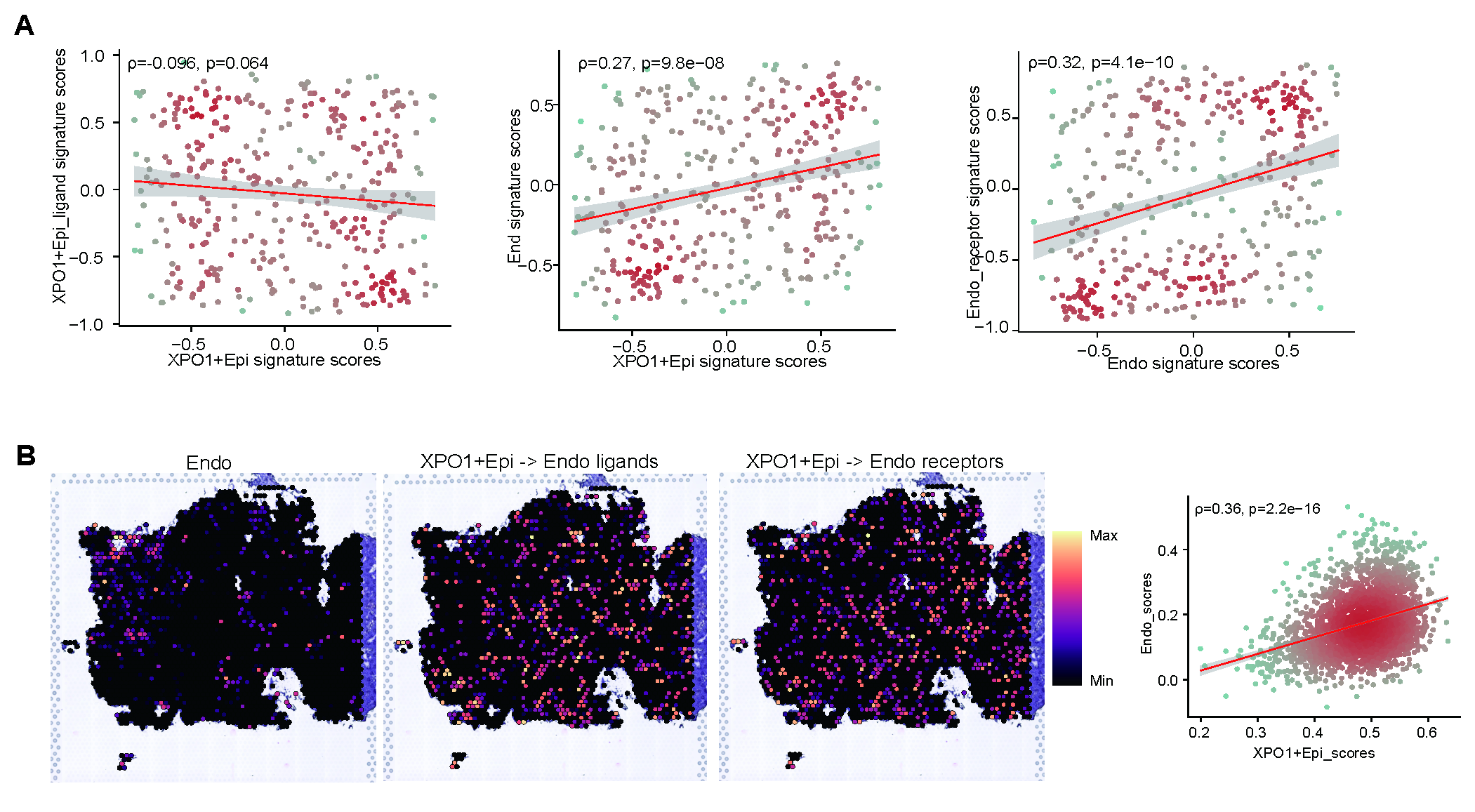

Supplement: Supplementary Figure 4 — XPO1+Epi and endothelial cell interactions in the TCGA-LIHC cohort and spatial transcriptome cohort correlations. (A) A scatterplot illustrating the correlation between XPO1+Epithelial and endothelial cells, along with their ligand receptors, in the TCGA-LIHC cohort (n=374). (B) ST-seq analysis revealing the spatial distribution and correlation between endothelial cells and ligand receptors of XPO1+Epithelial interacting with endothelial cells in HCC. [file Image4.tiff]

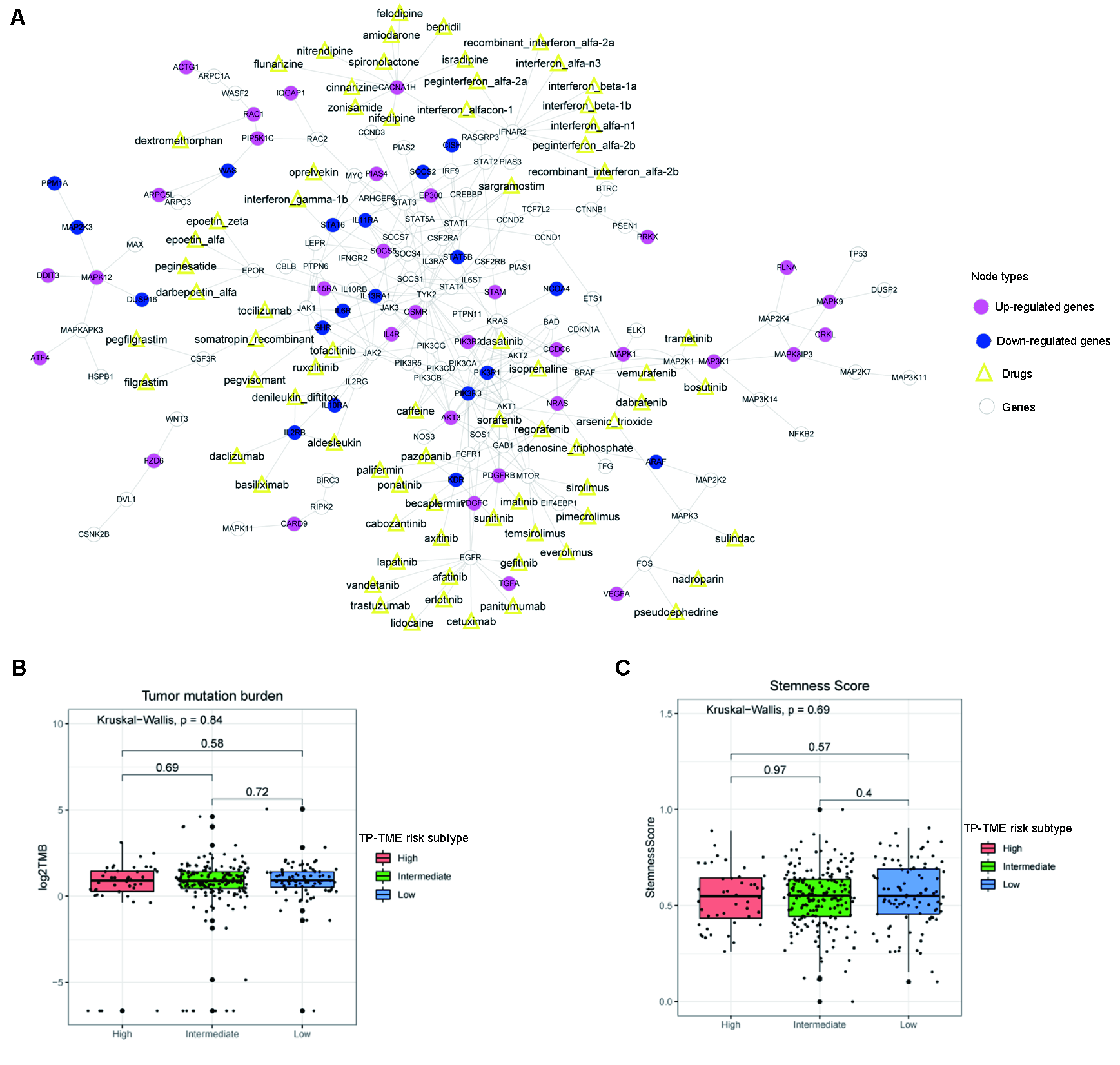

Supplement: Supplementary Figure 5 — Drug-target networks for potentially effective drugs for the TP-TME high-risk subtypes. (A) Drug-target networks for potentially effective drugs for the TP-TME high-risk subtypes. Red represents upregulated, and blue represents downregulated in the TP-TME high-risk subtypes. The Wilcox test was used to assess the differences between groups. (B) Tumor mutation burden scores of the three TP-TME risk subtypes. (C) Stemness scores of the three TP-TME risk subtypes. [file Image5.tiff]
