# Supplementary material for: Integrative multi-omics analysis reveals a novel subtype of hepatocellular carcinoma with biological and clinical relevance
Source: Front Immunol. 2024 Dec 6;15:1517312. doi: 10.3389/fimmu.2024.1517312 (PMC11659151; doi:10.3389/fimmu.2024.1517312)

High Ki67 expression

Tumor Adjacent

Patient1

XPO1

RCN2

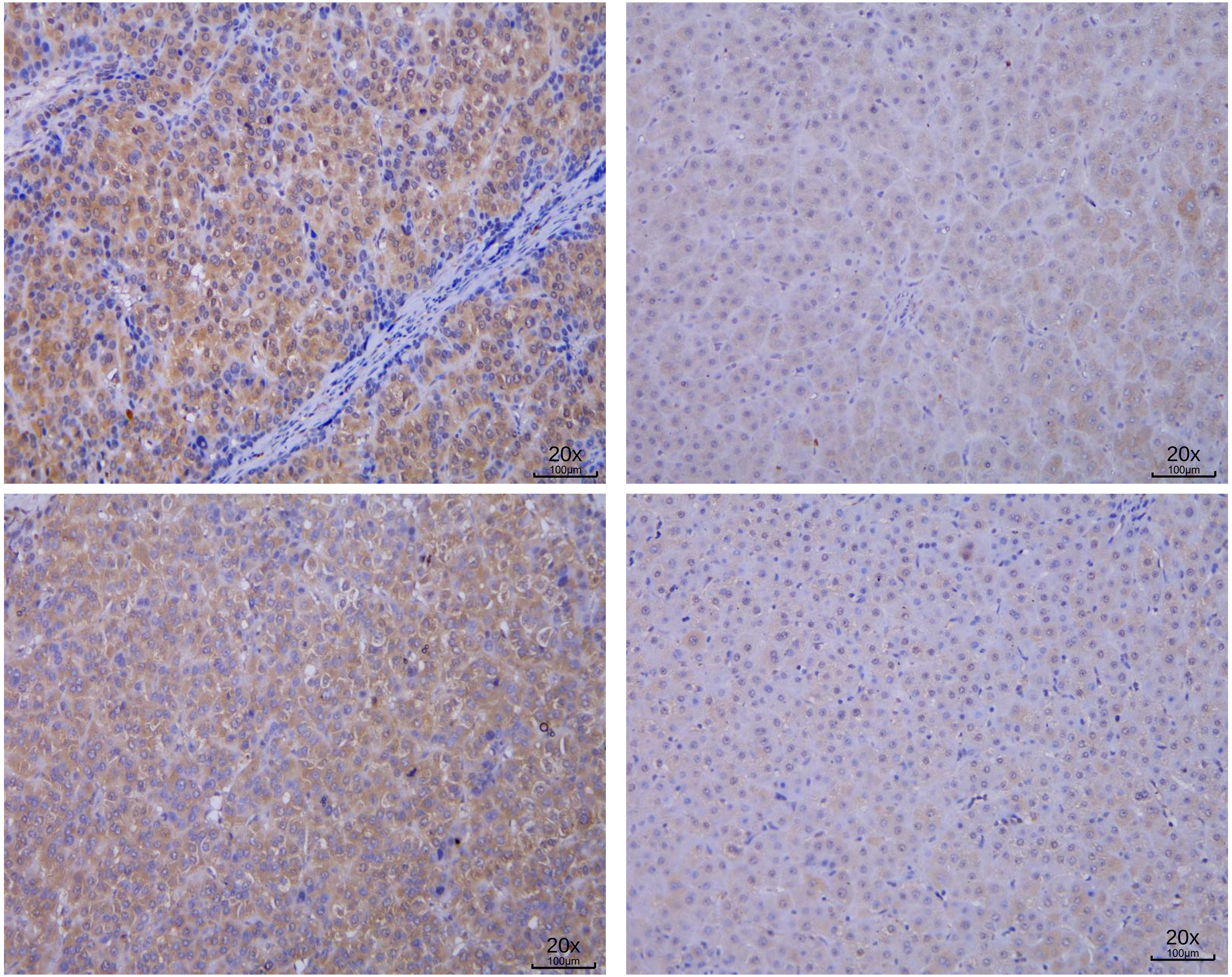

High Ki67 expression

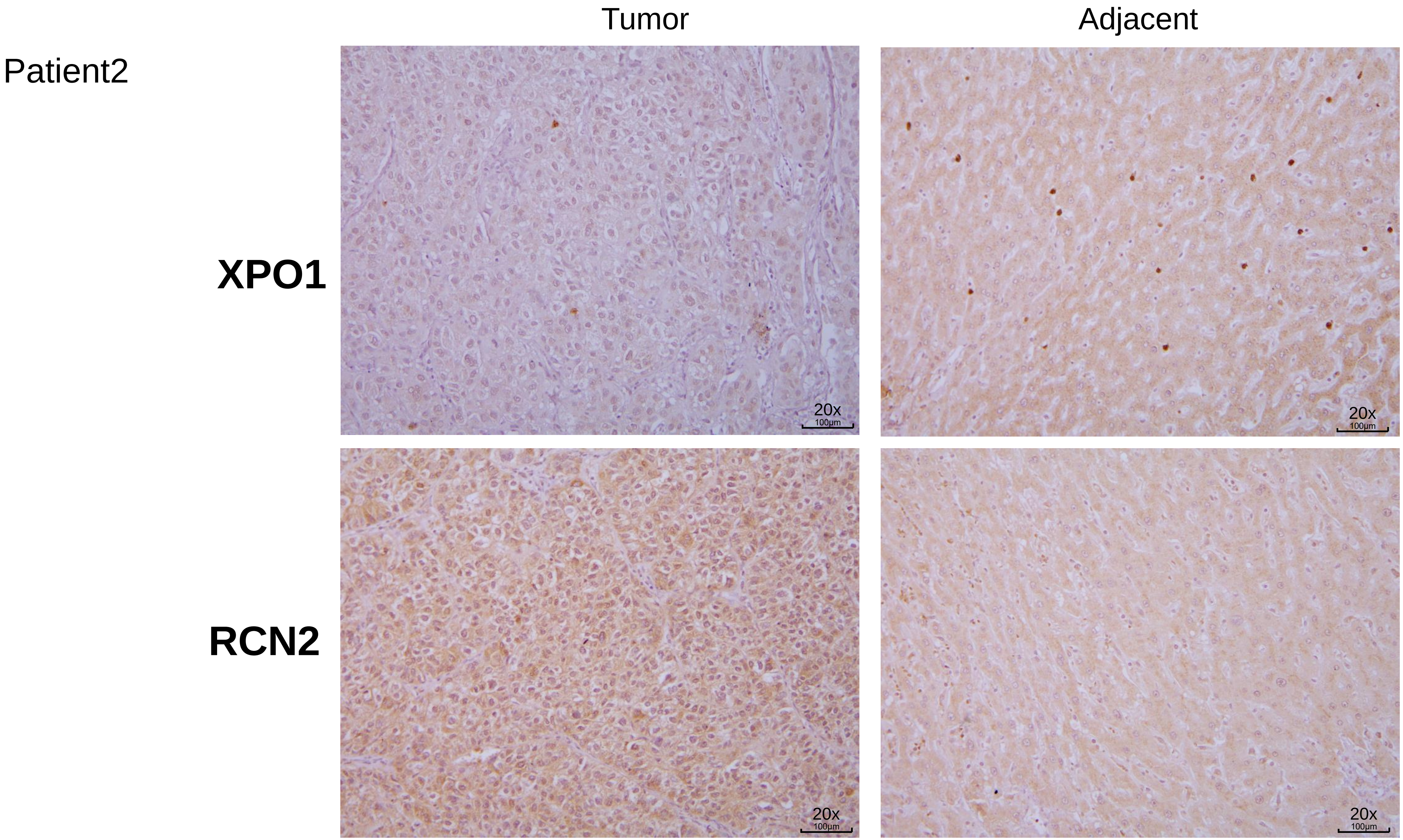

High Ki67 expression

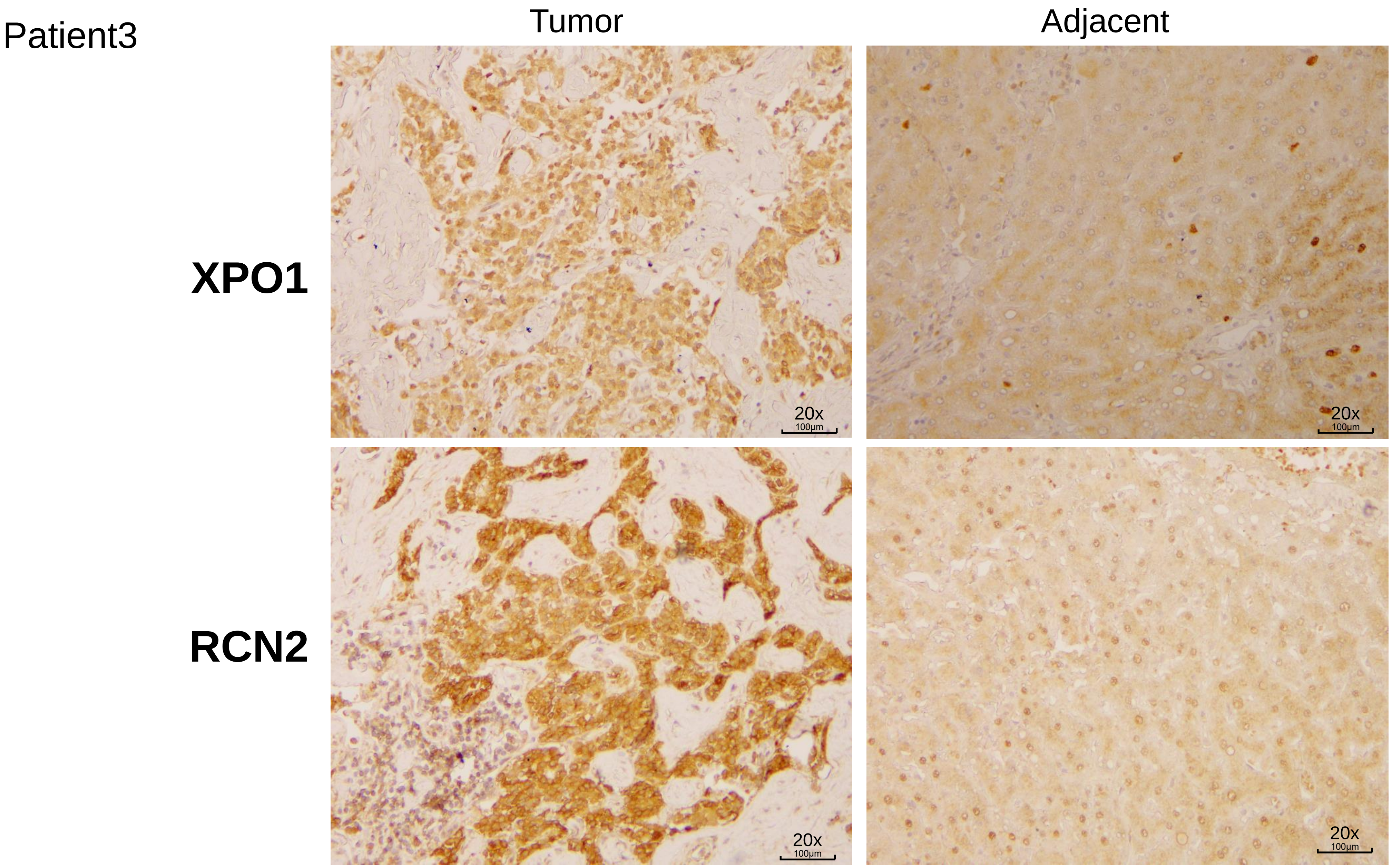

High Ki67 expression

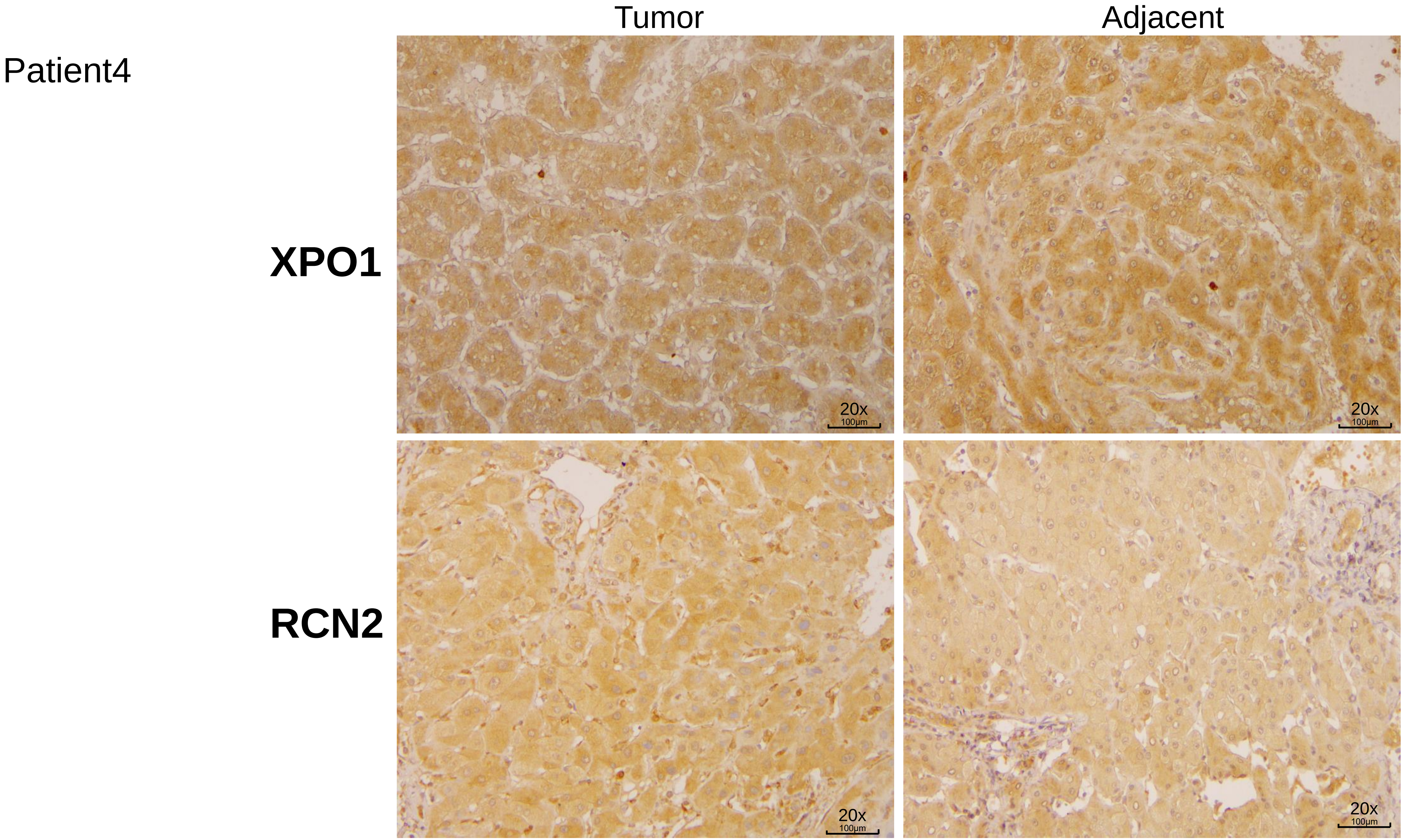

High Ki67 expression

Patient5

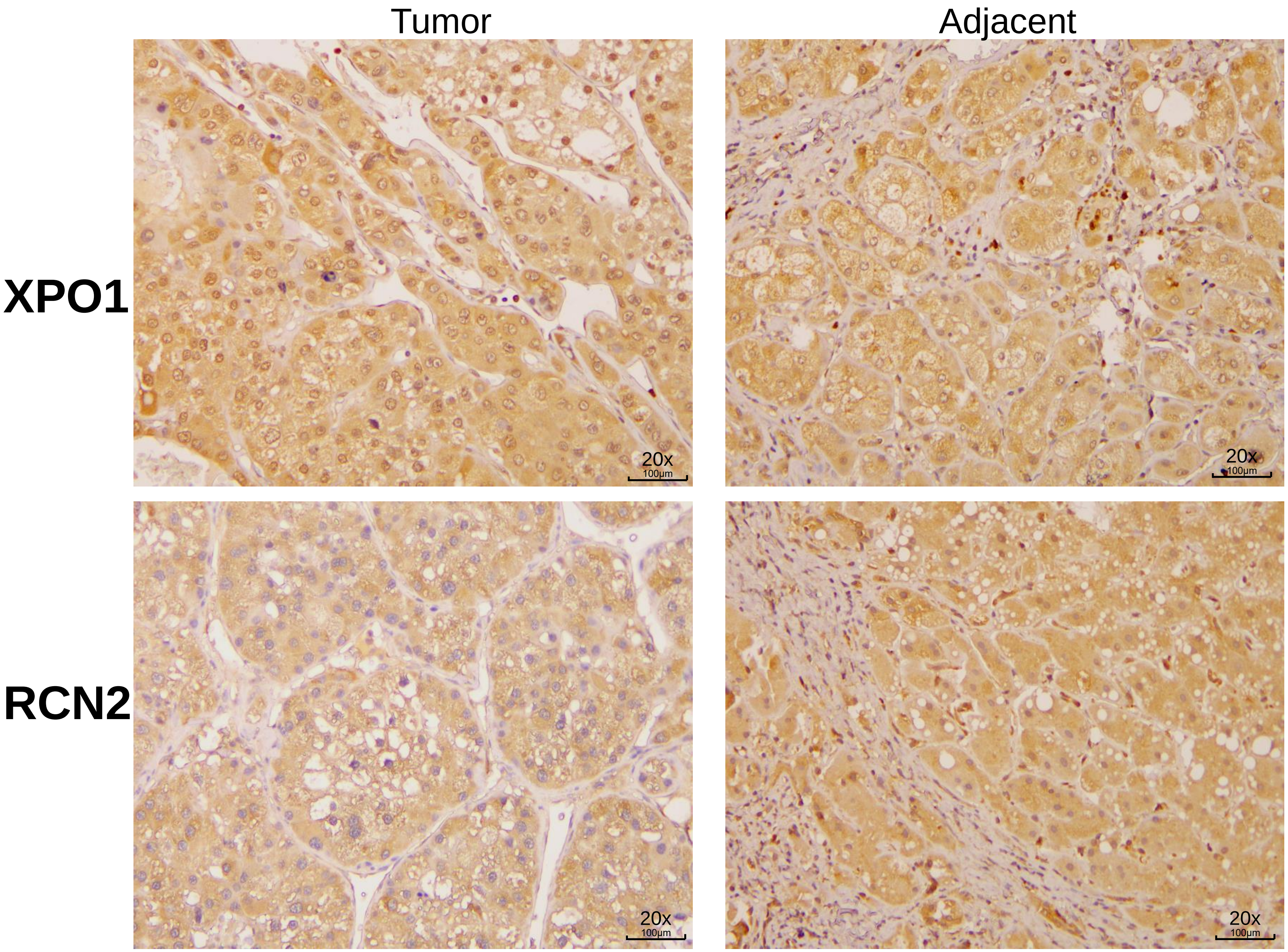

High Ki67 expression

Patient 6

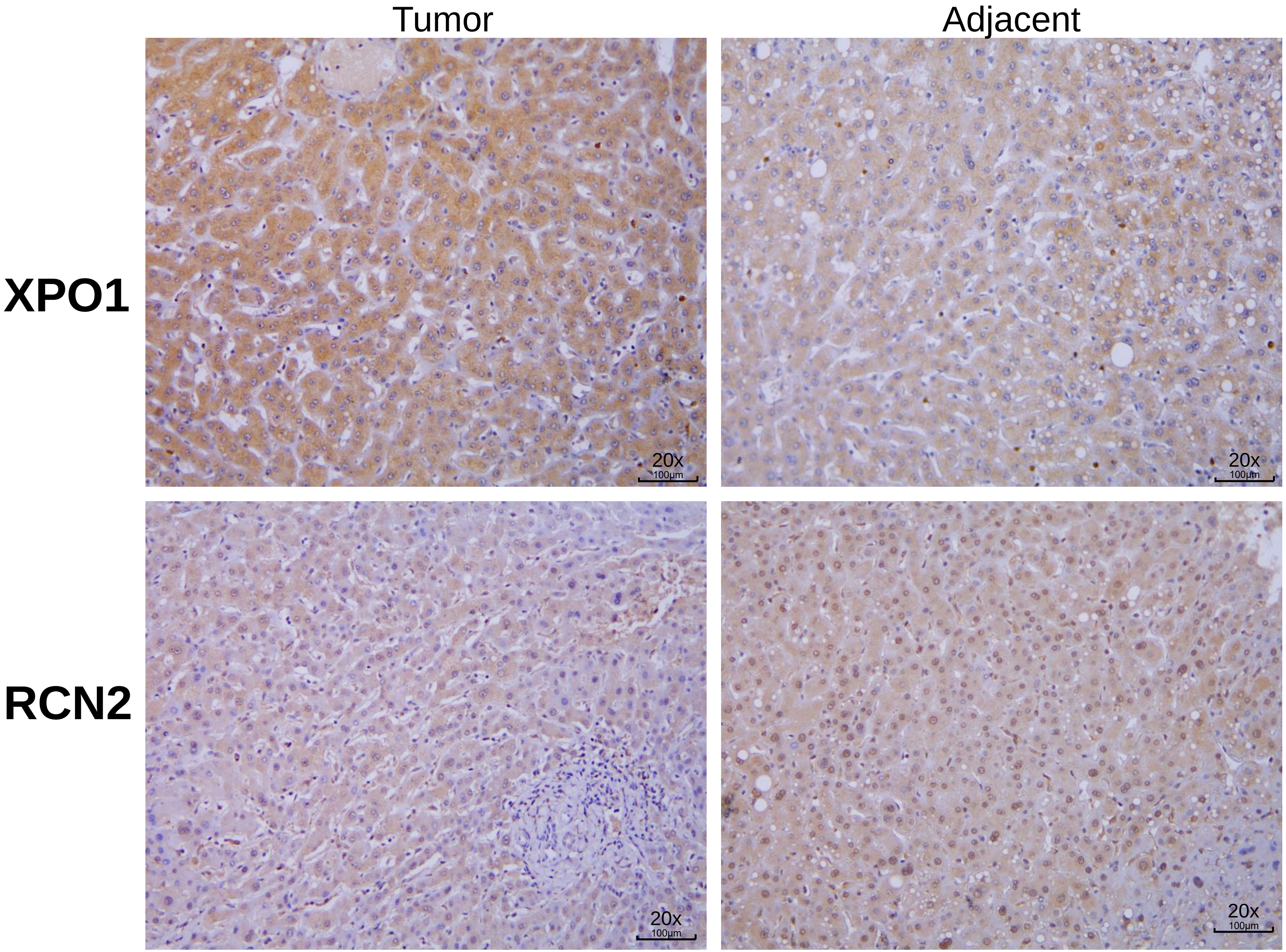

High Ki67 expression

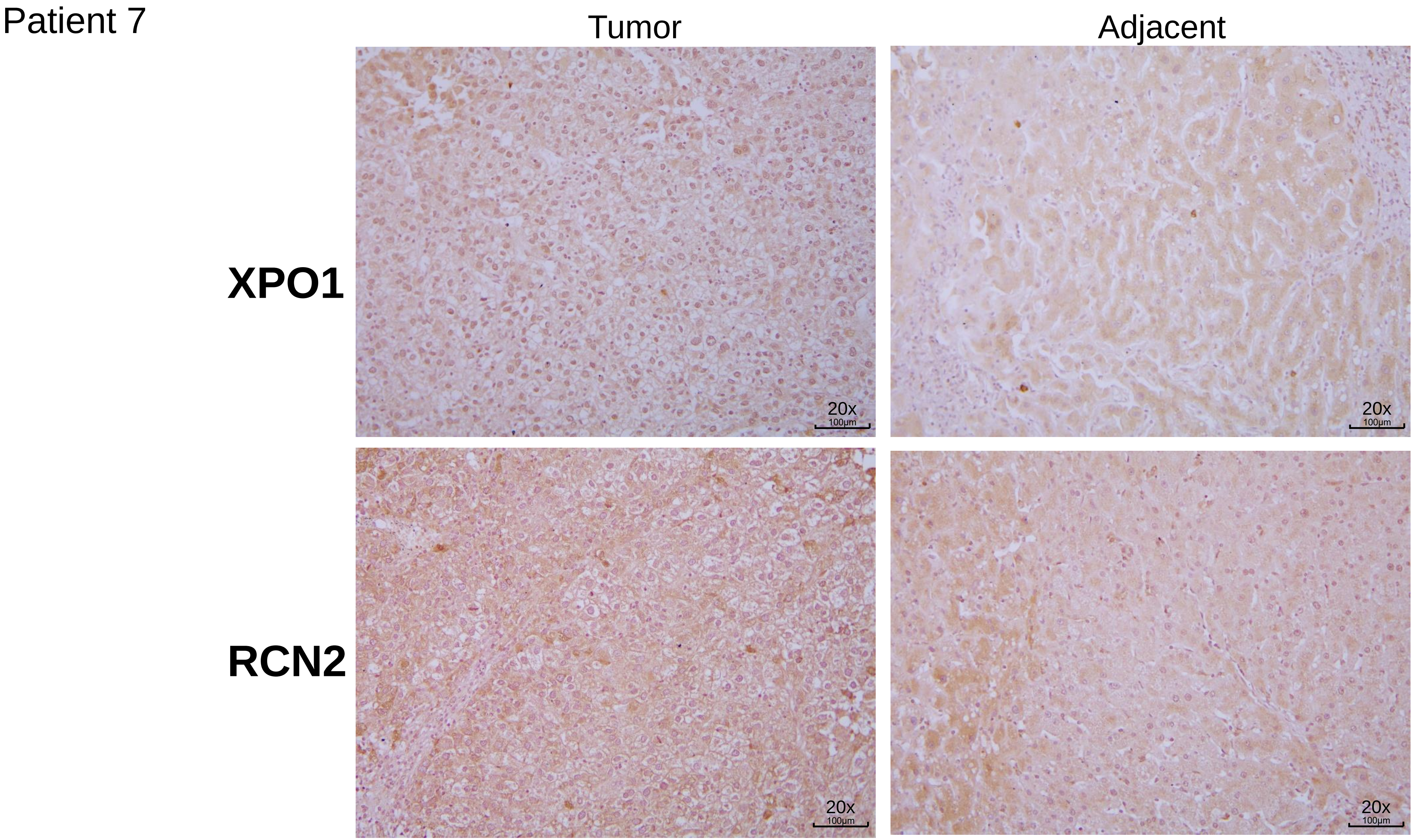

High Ki67 expression

Patient 8

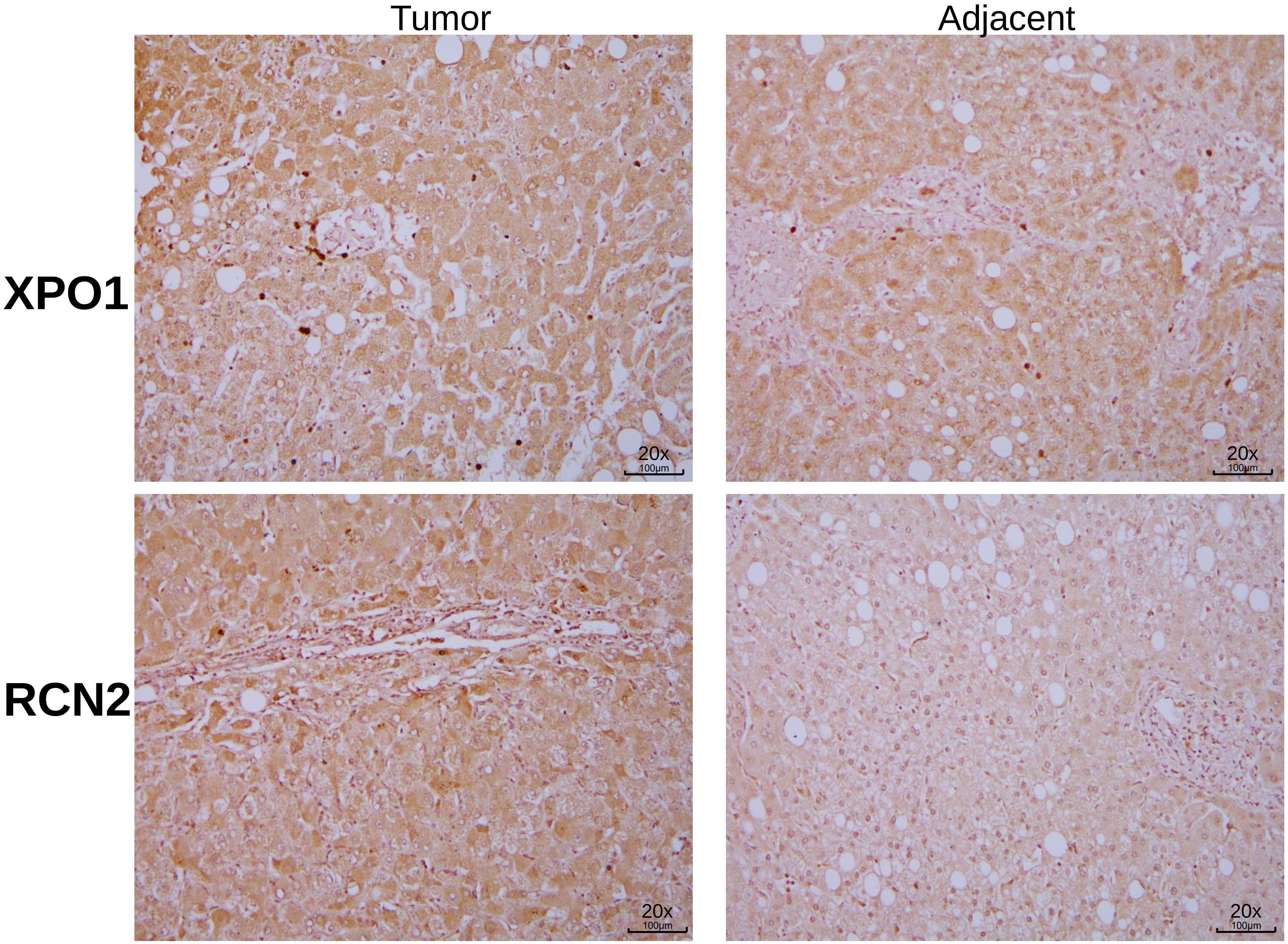

High Ki67 expression

Patient 9

Tumor | Adjacent

XPO1

RCN2

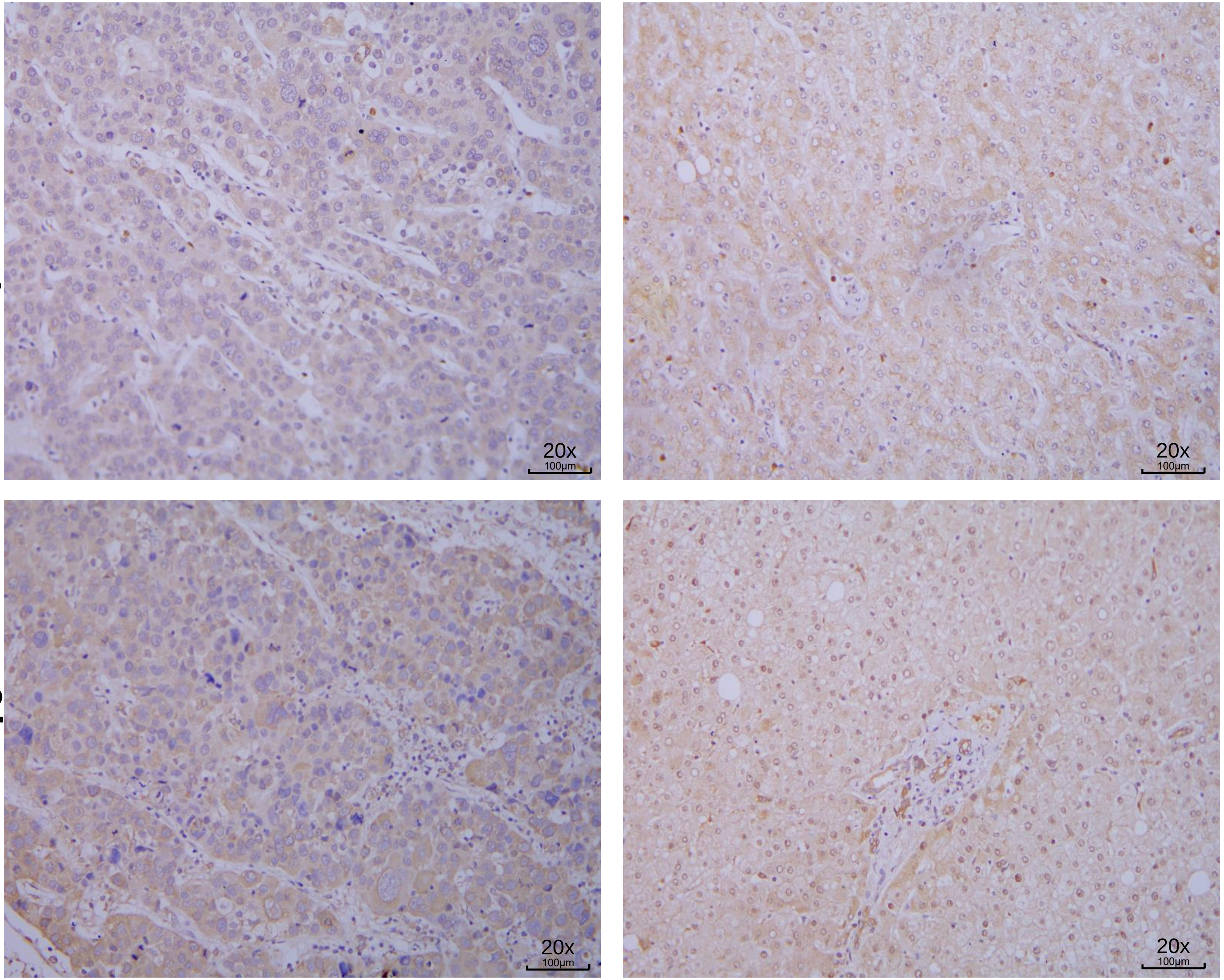

High Ki67 expression

Patient 10

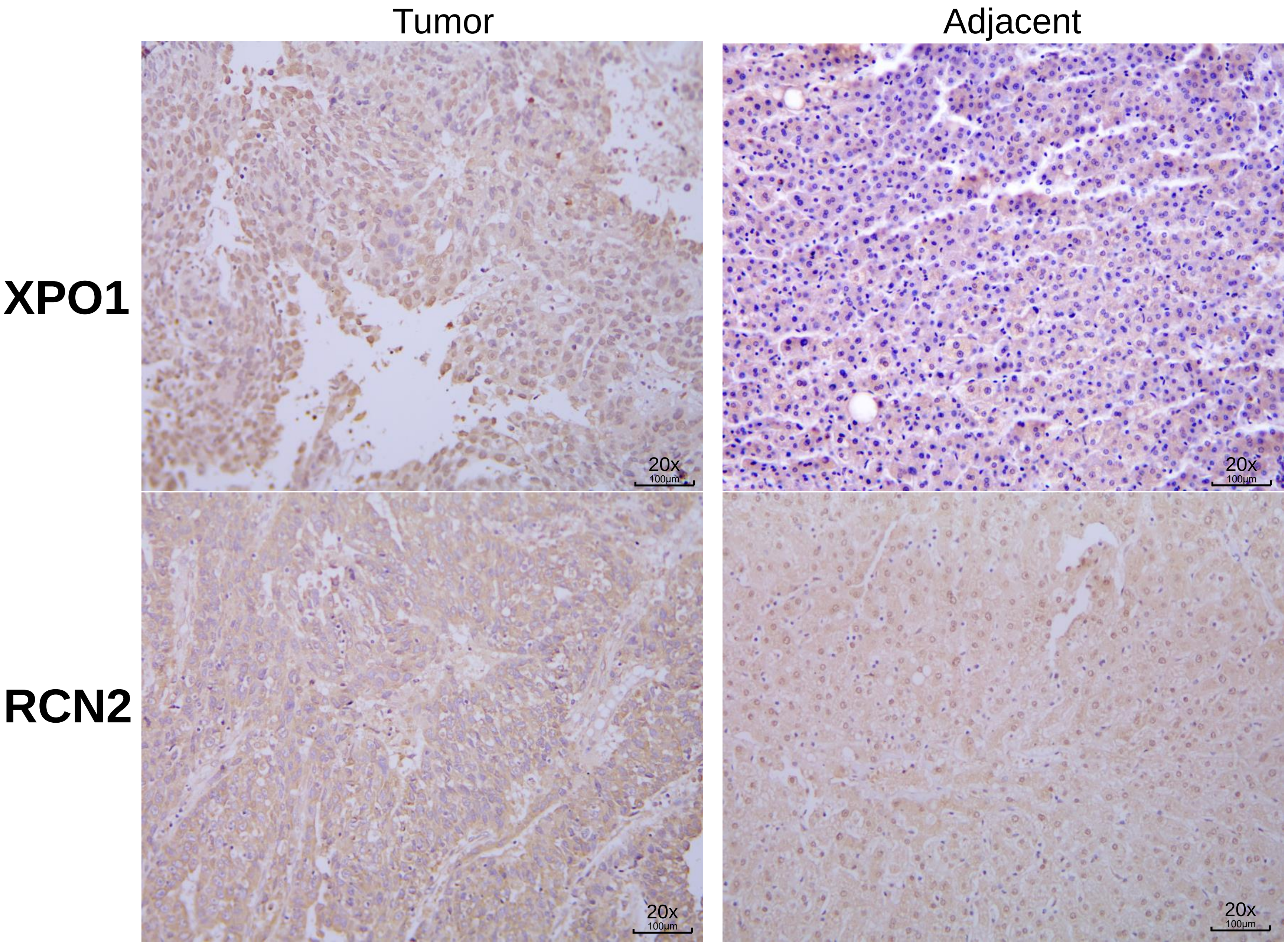

High Ki67 expression

Patient 11

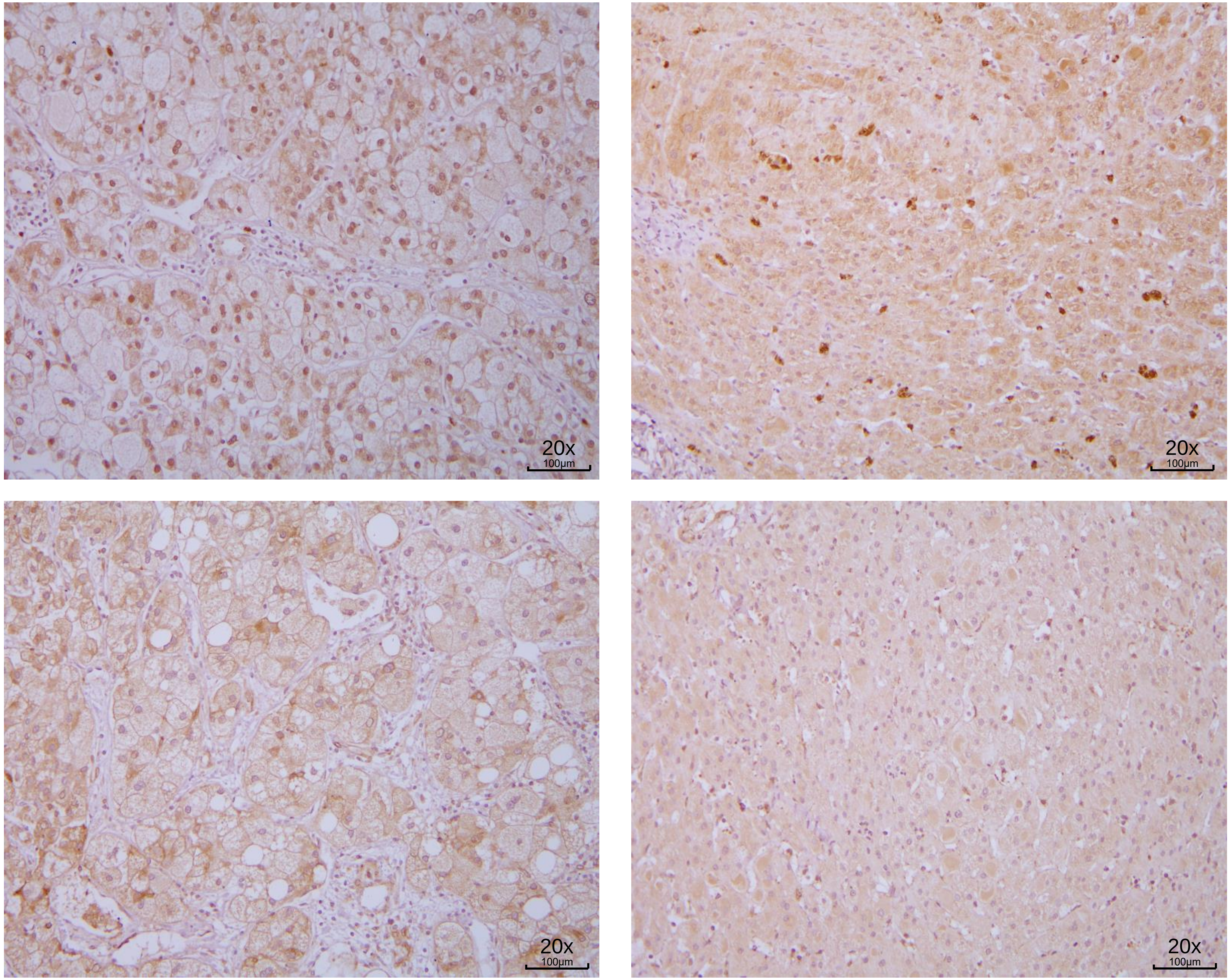

High Ki67 expression

Patient 12

Tumor | Adjacent

XPO1

RCN2

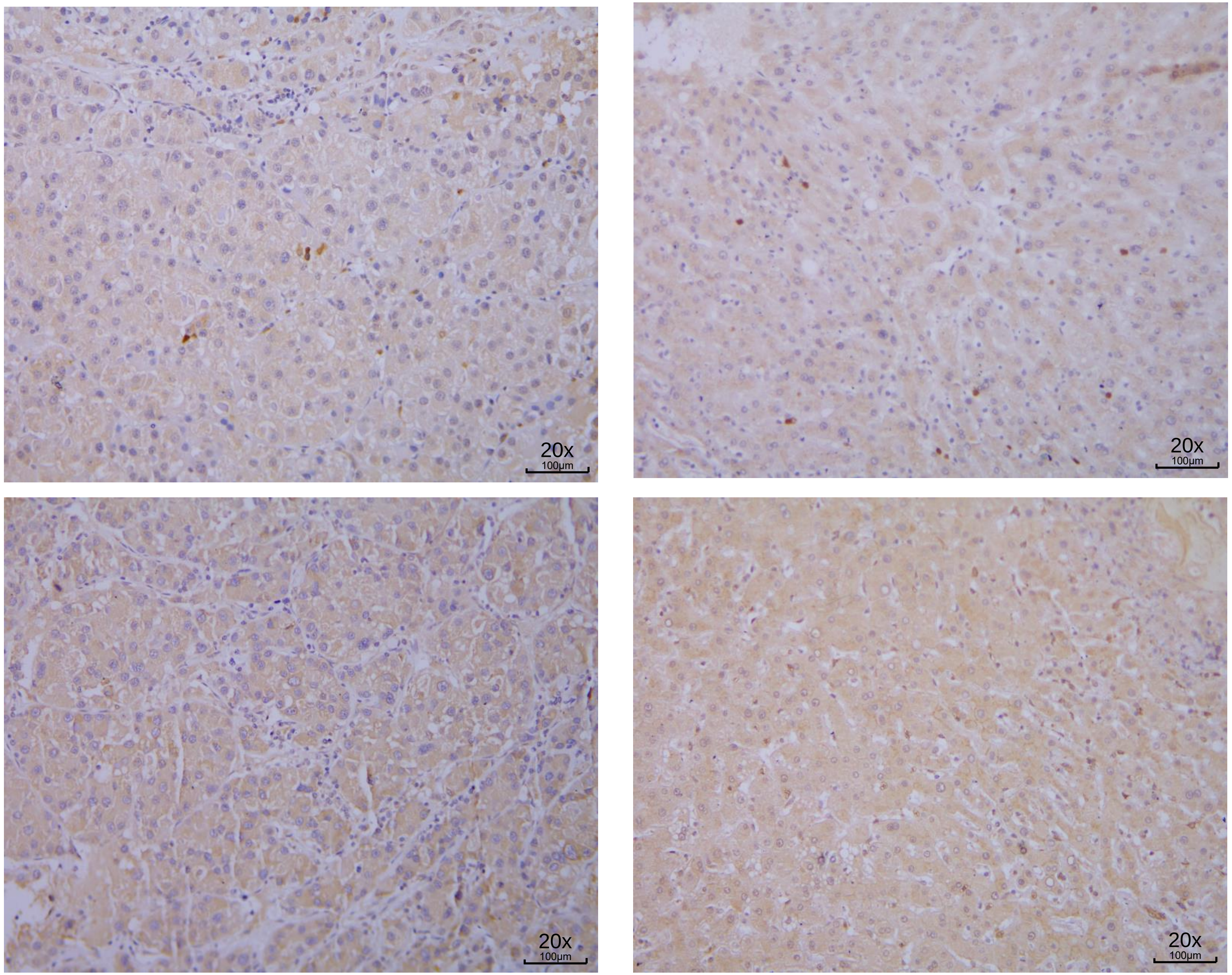

High Ki67 expression

Patient 13

Tumor | Adjacent

XPO1

RCN2

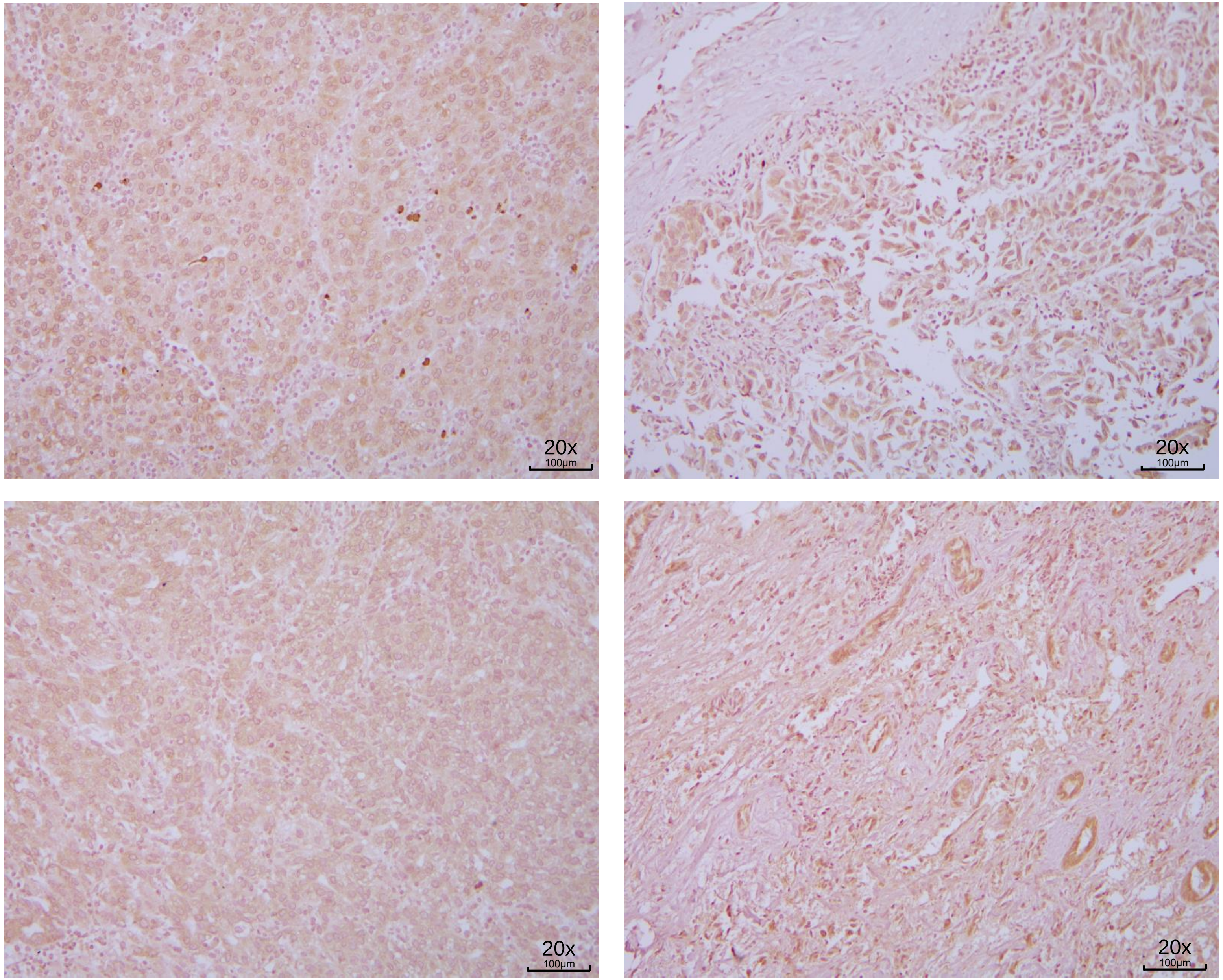

High Ki67 expression

Patient 14

Tumor | Adjacent

XPO1

RCN2

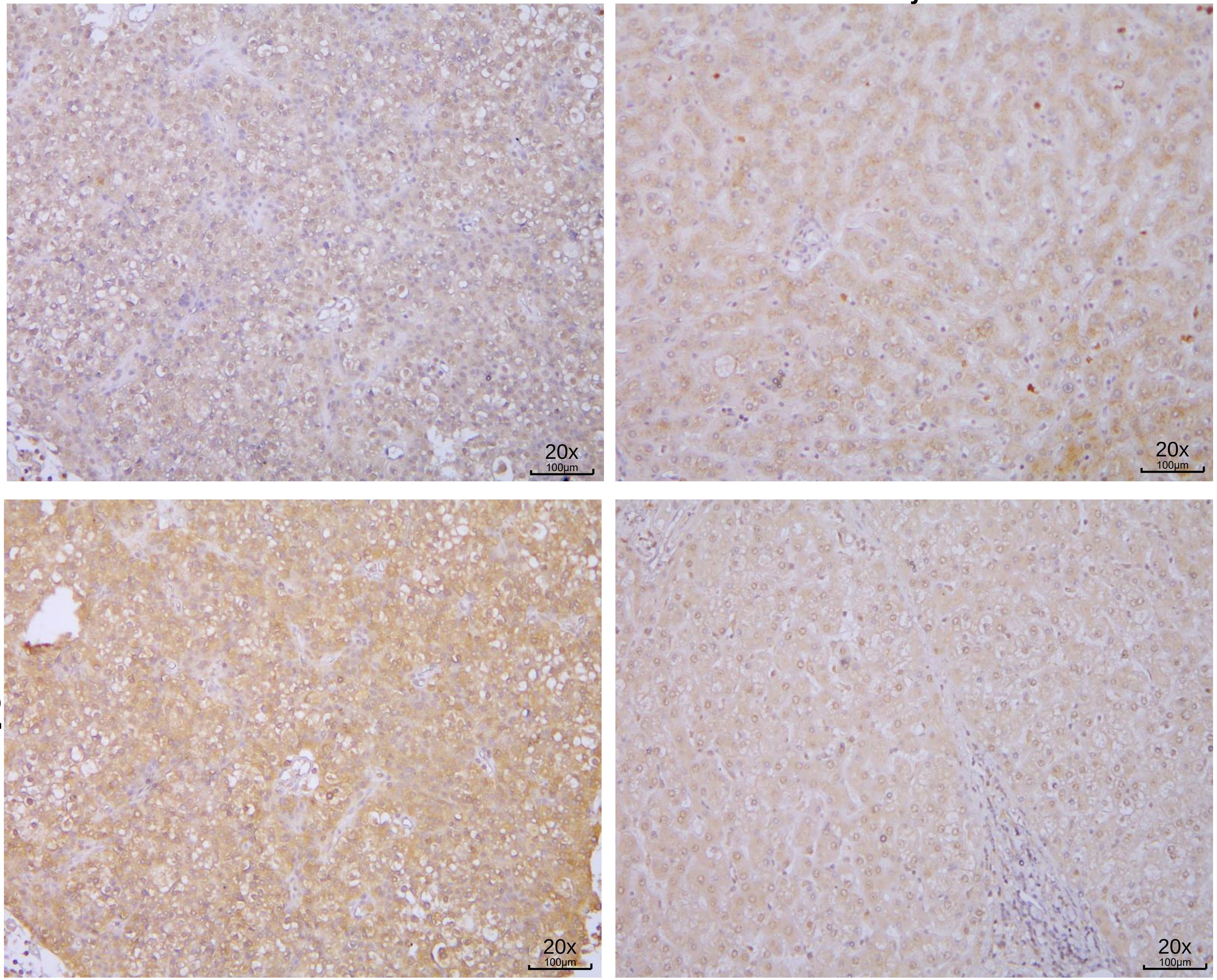

High Ki67 expression

Patient 15

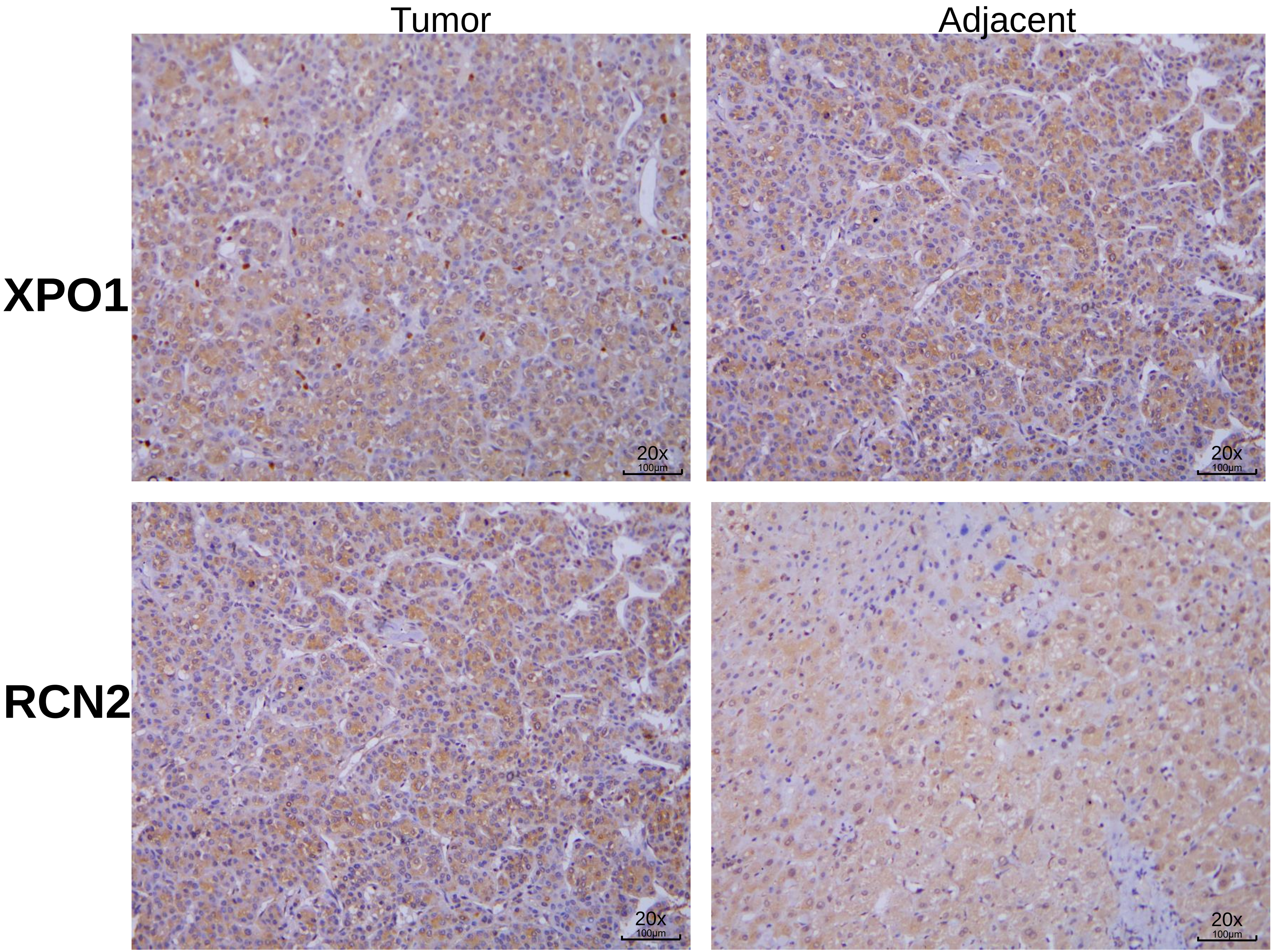

High Ki67 expression

Patient 16

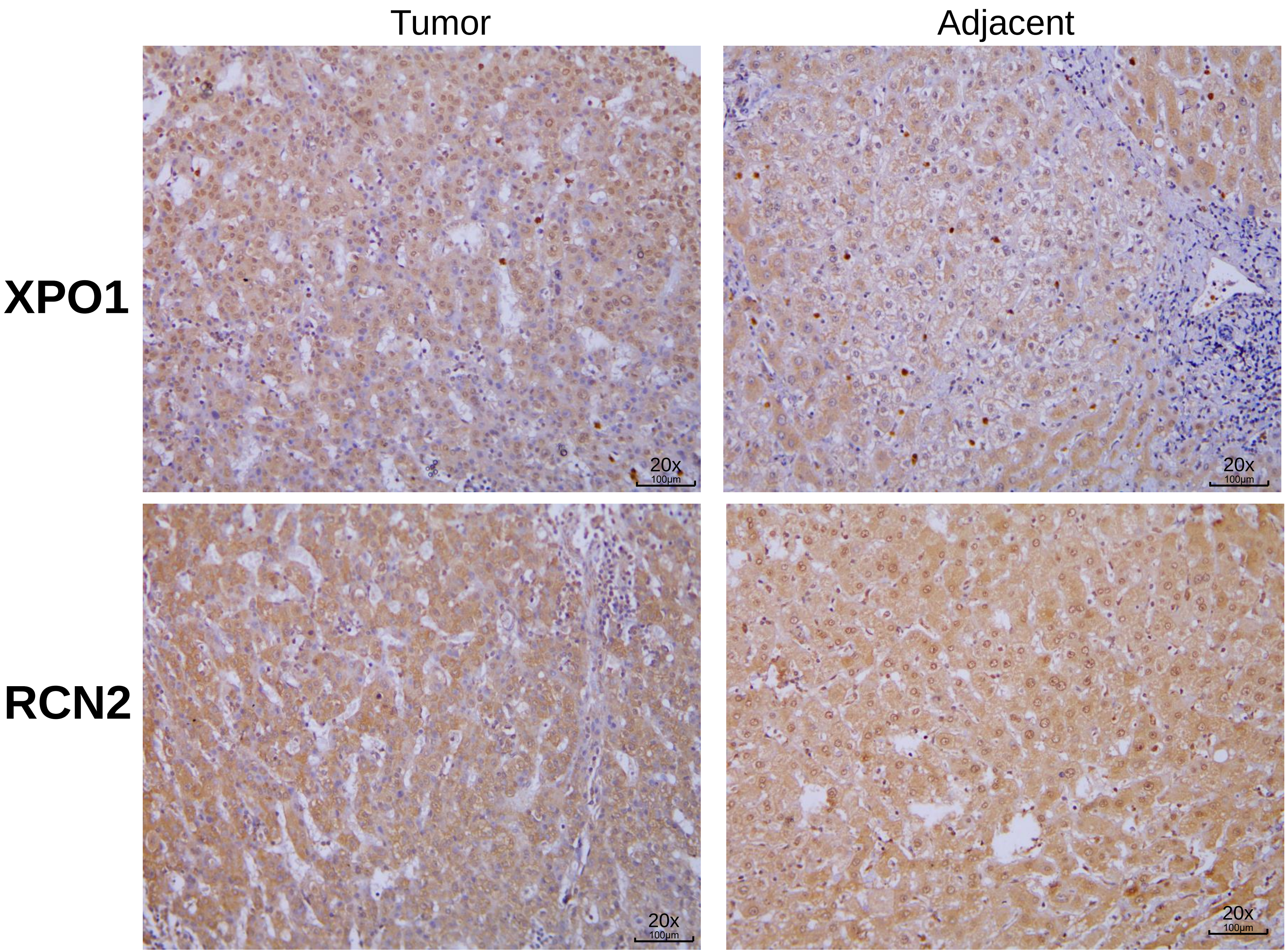

High Ki67 expression

Patient 17

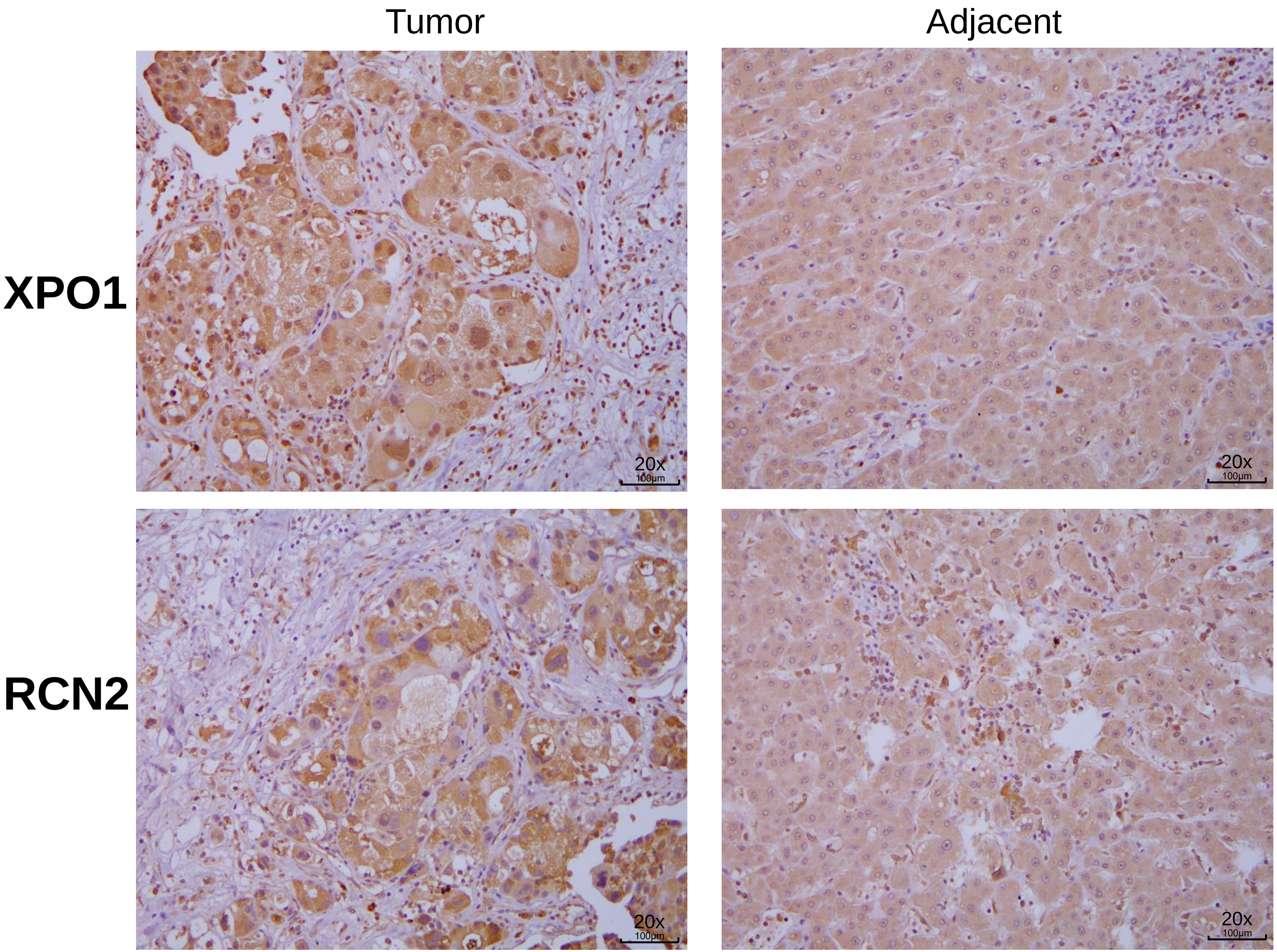

High Ki67 expression

Patient18

Tumor Adjacent

XPO1

RCN2

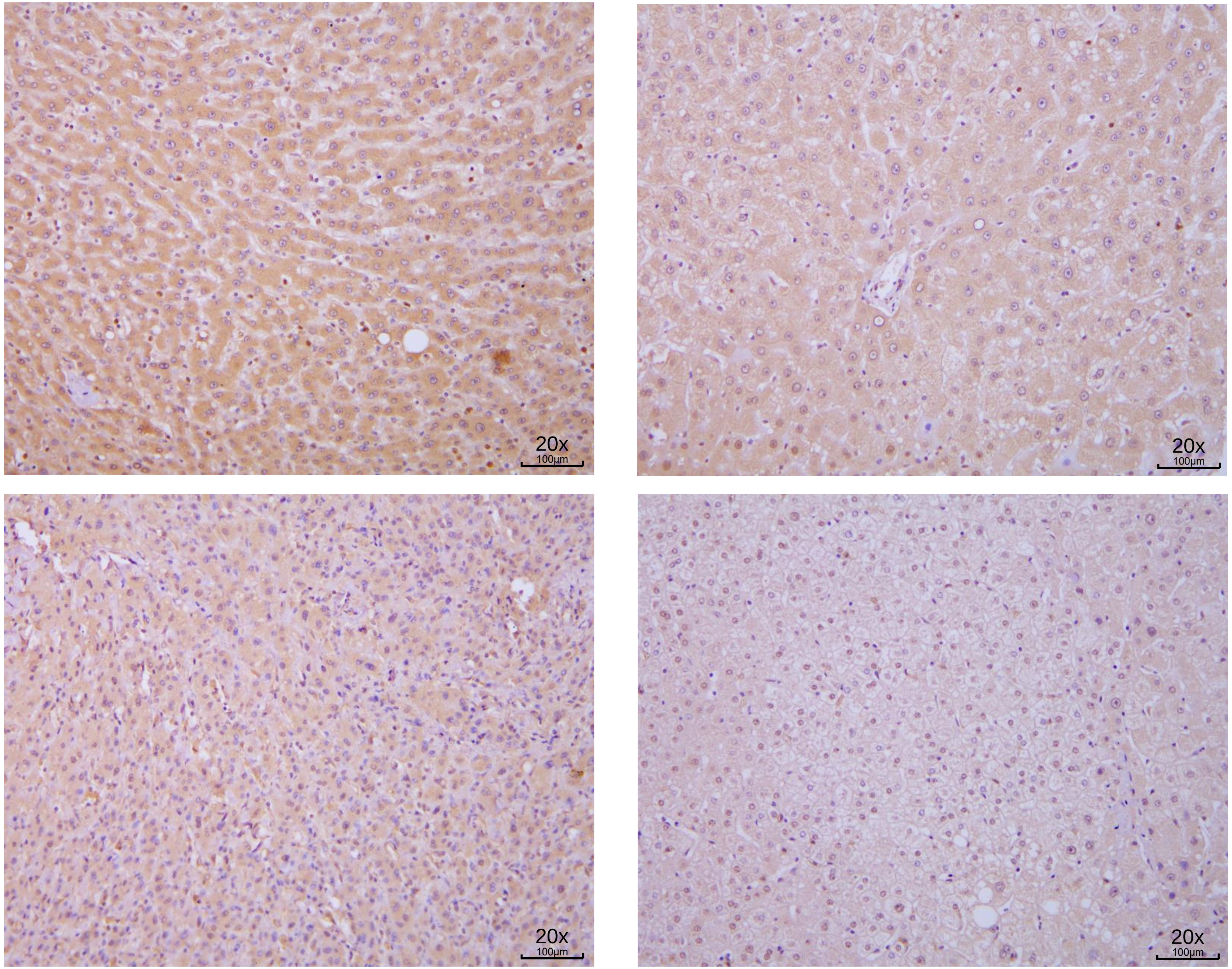

High Ki67 expression

Patient 19

Tumor | Adjacent

XPO1

RCN2

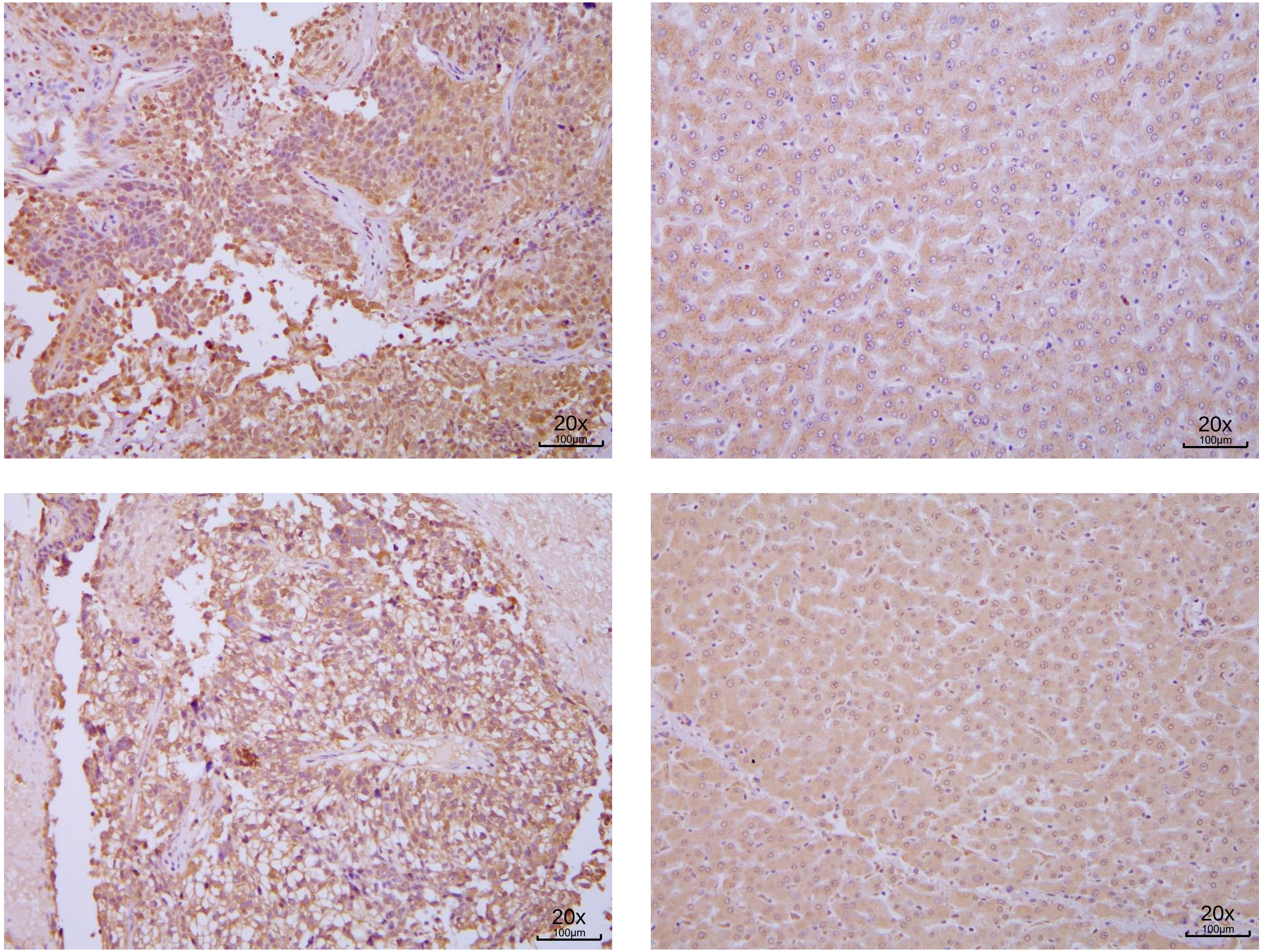

High Ki67 expression

Patient 20

Tumor | Adjacent

XPO1

RCN2

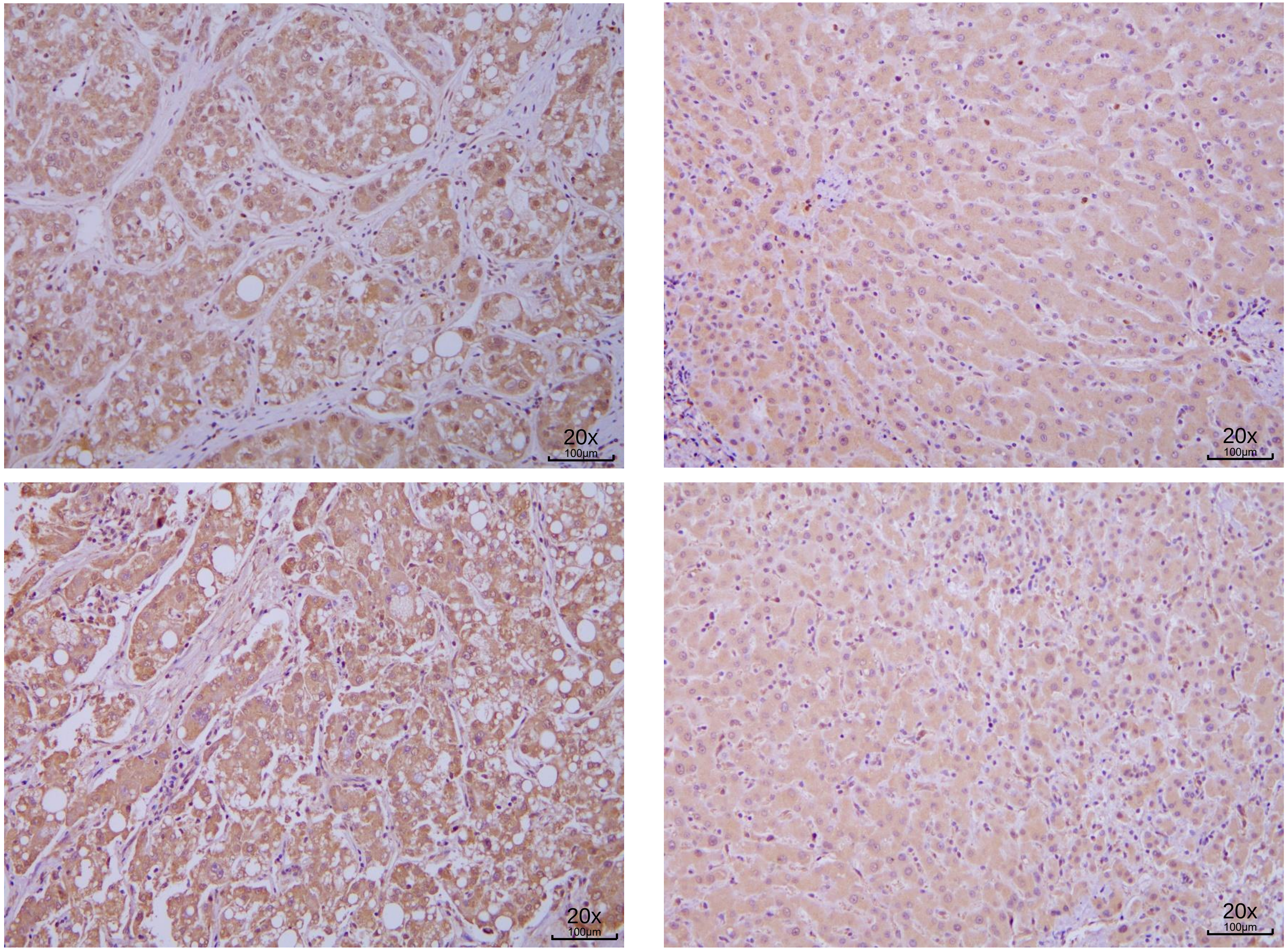

High Ki67 expression

Patient 21

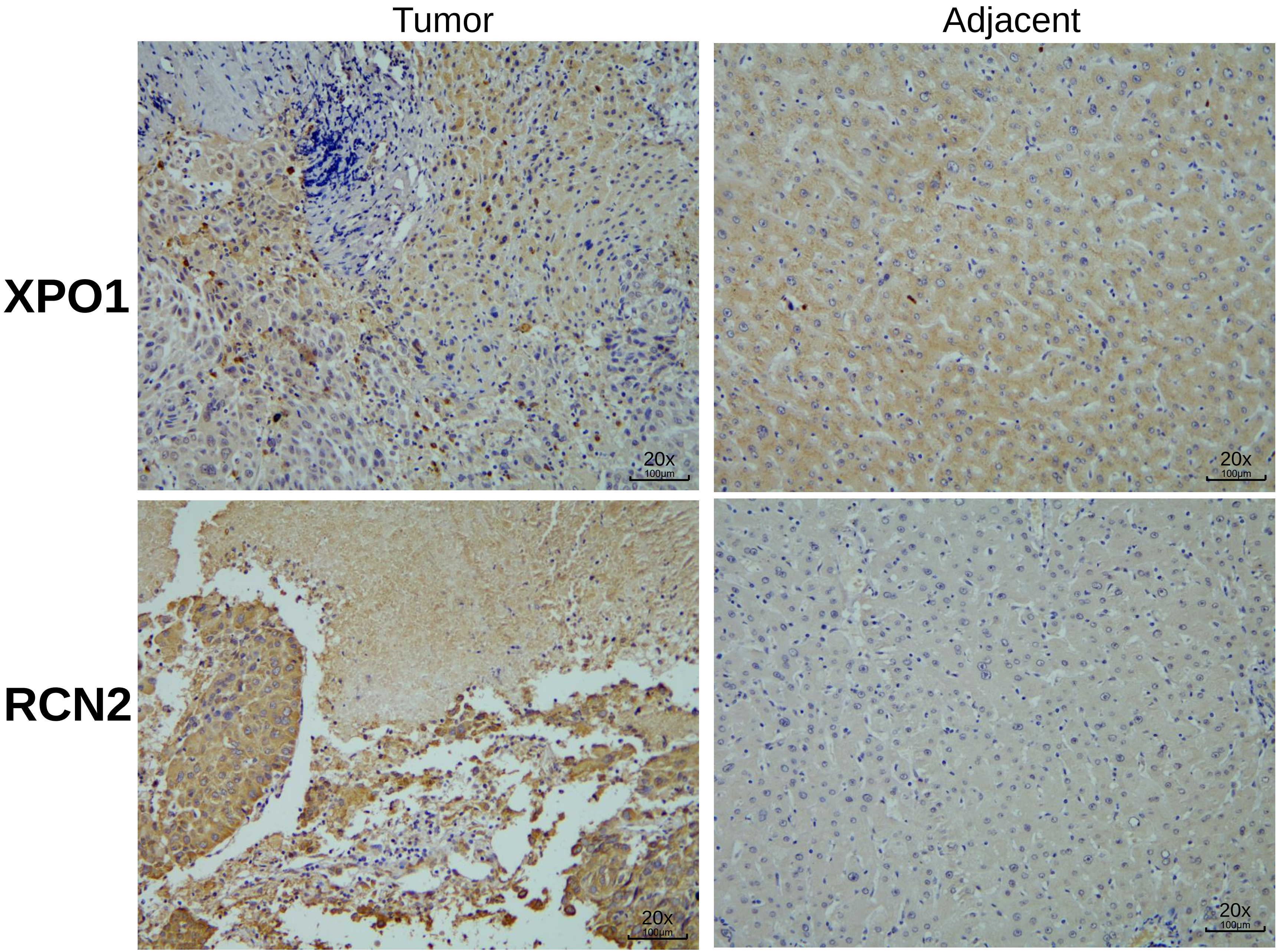

High Ki67 expression

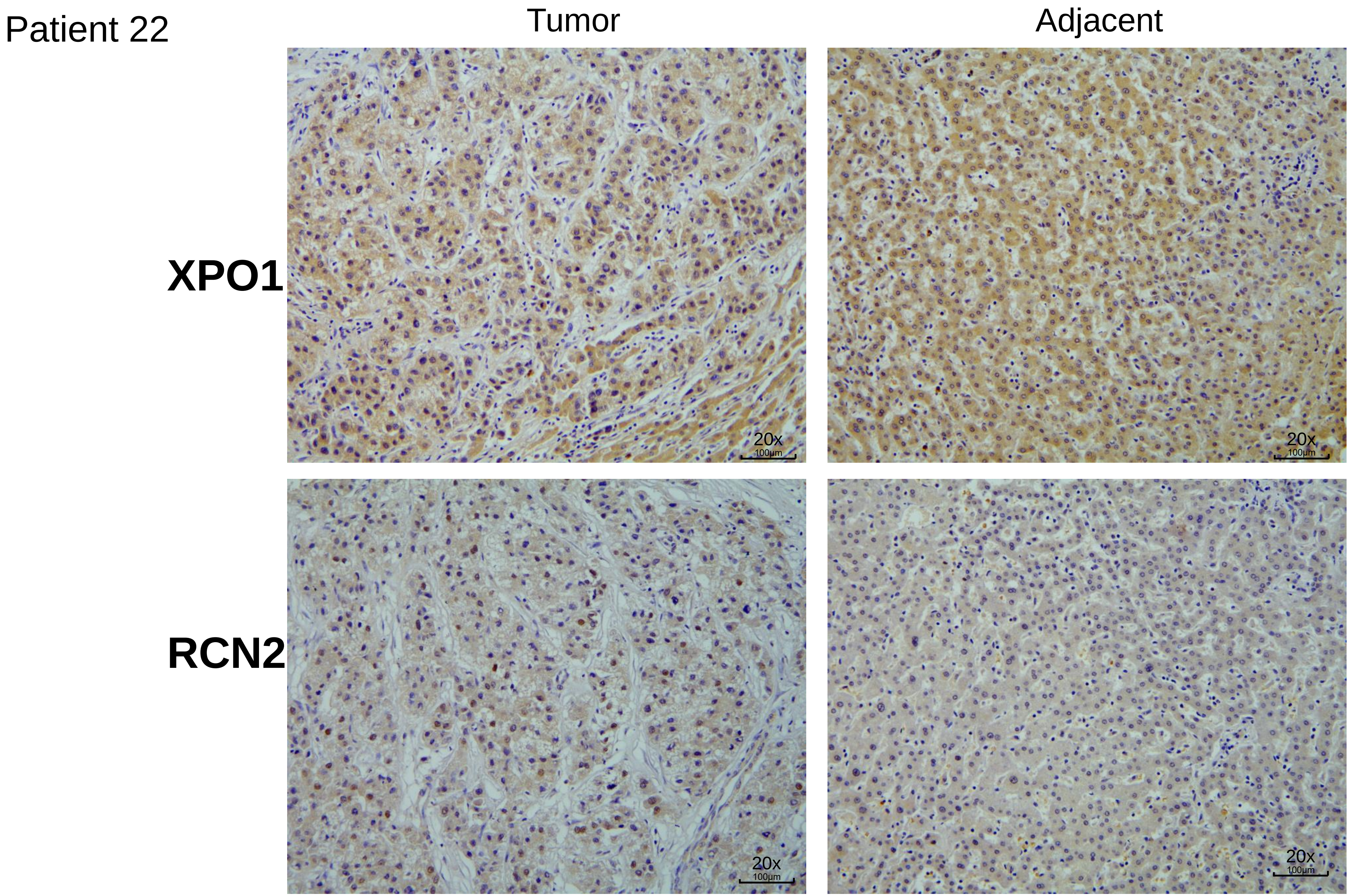

High Ki67 expression

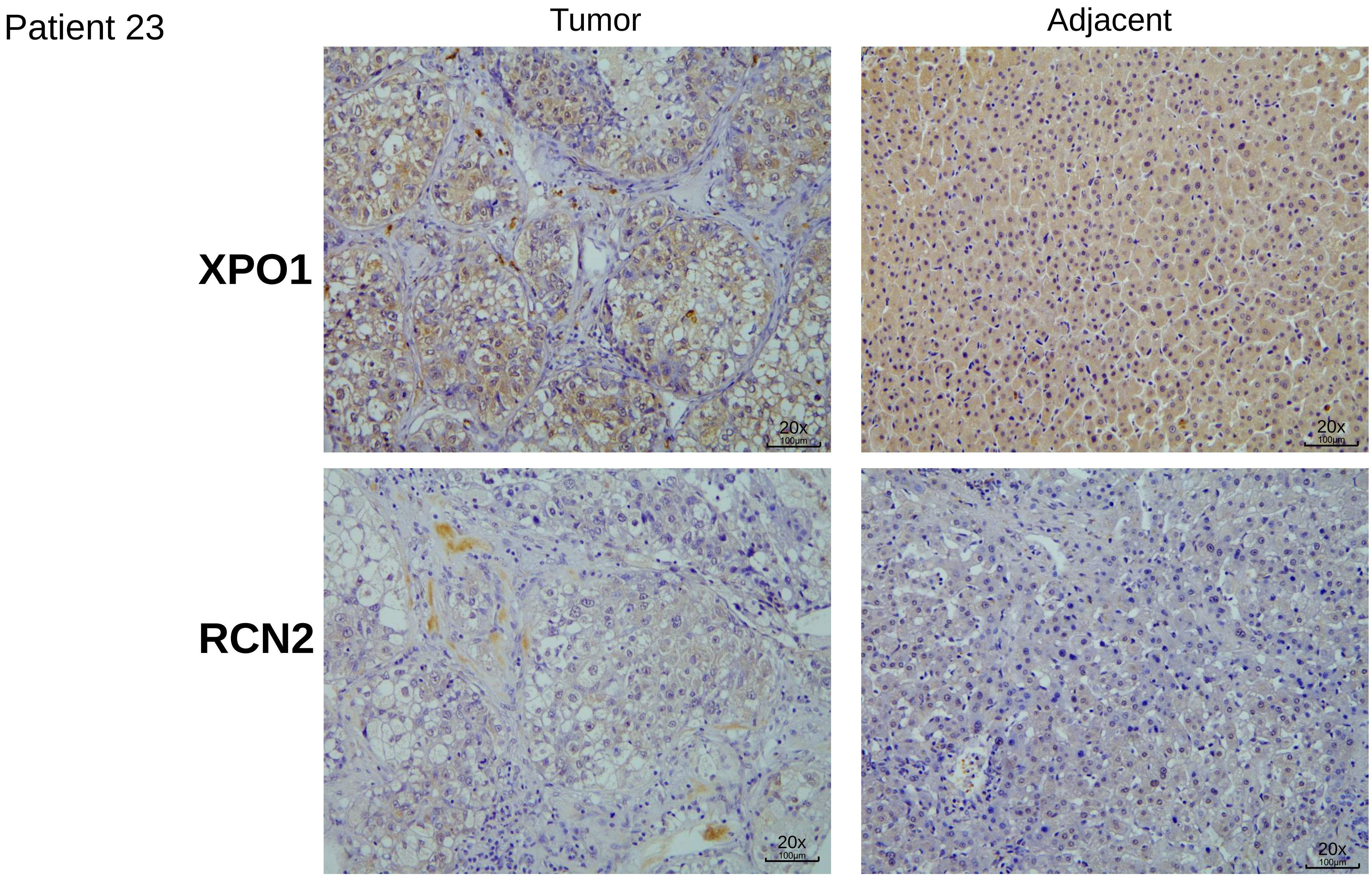

Low Ki67 expression

Patient1

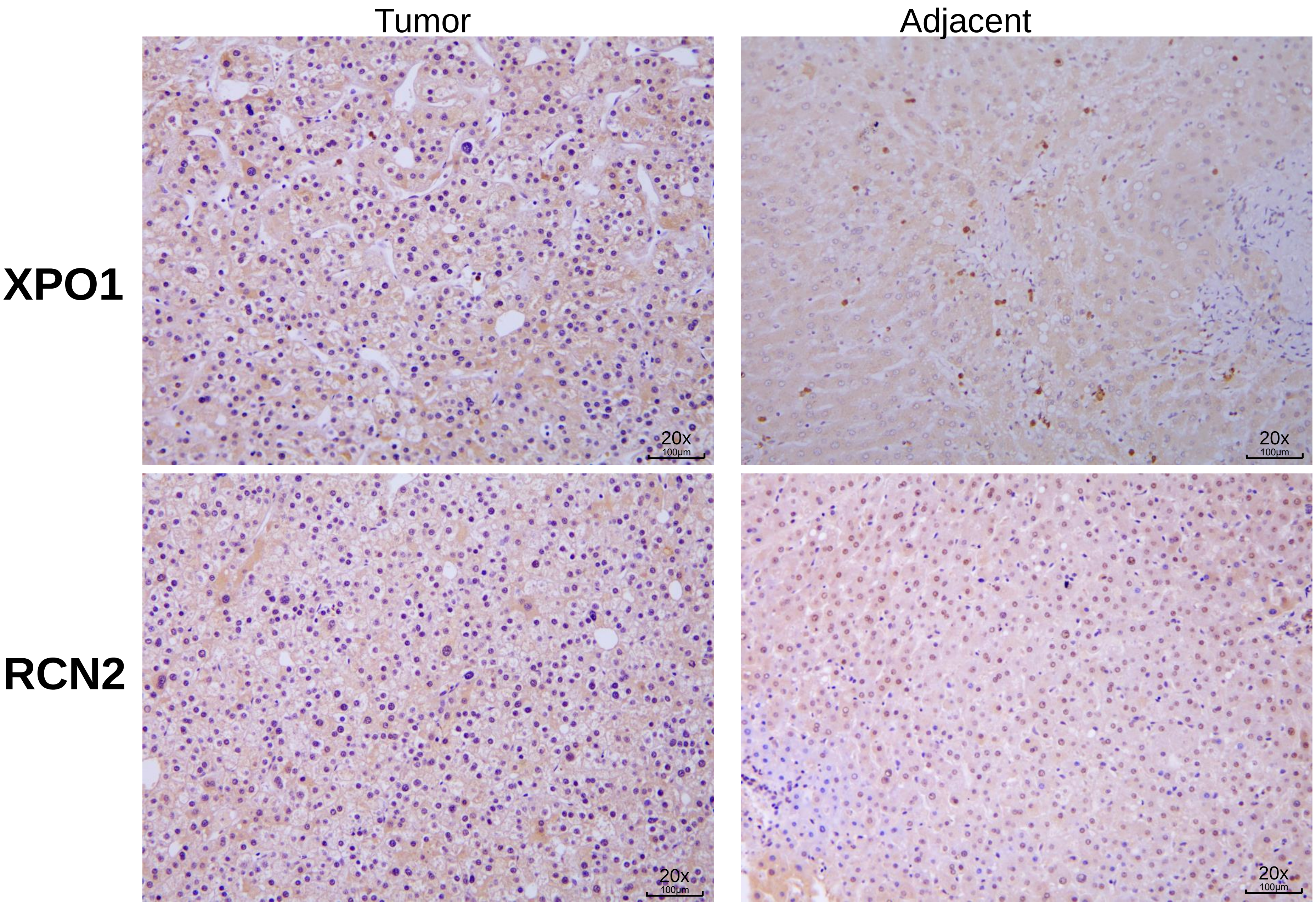

Low Ki67 expression

Patient 2

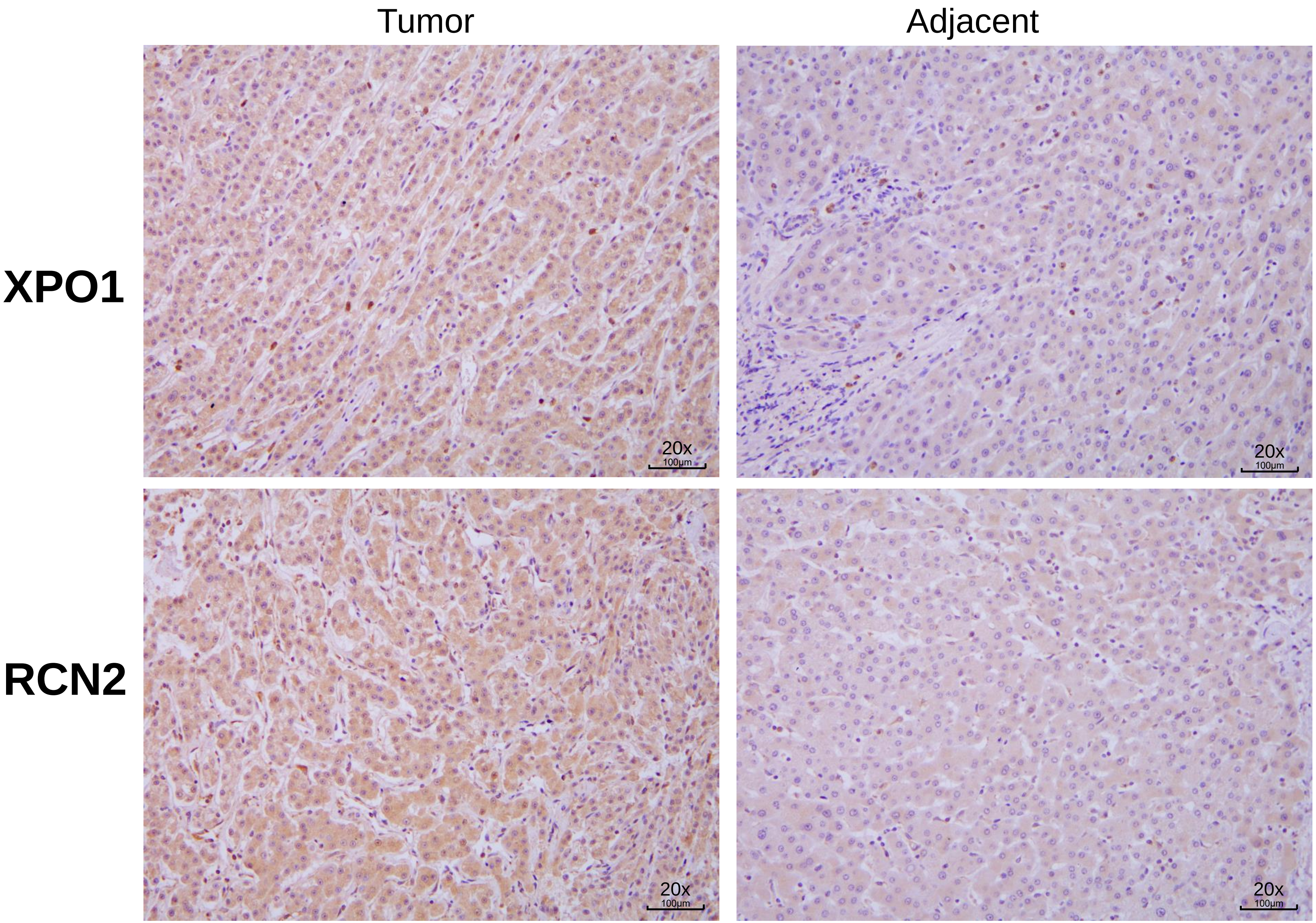

Low Ki67 expression

Patient3

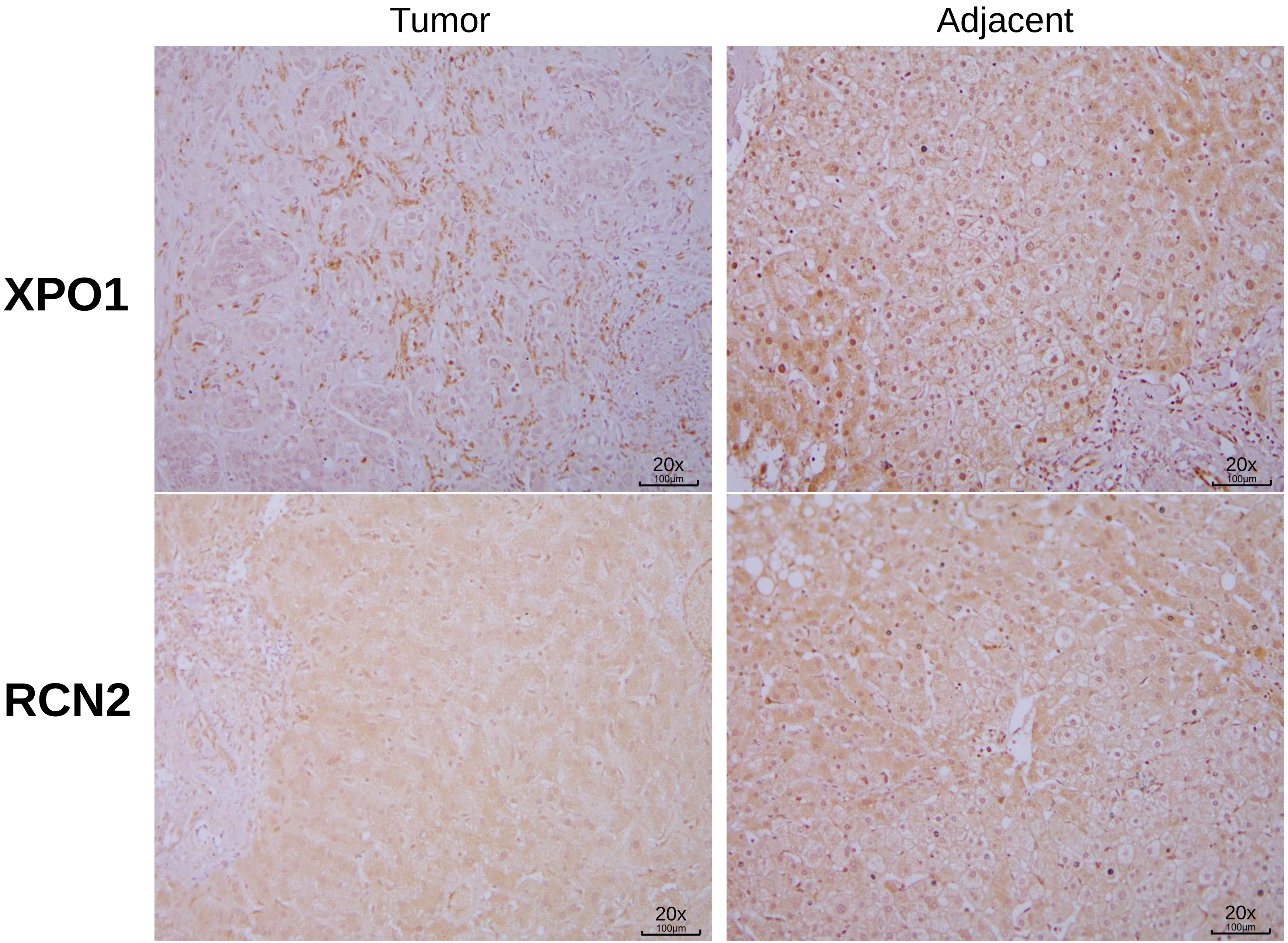

Low Ki67 expression

Patient 4

Tumor | Adjacent

XPO1

RCN2

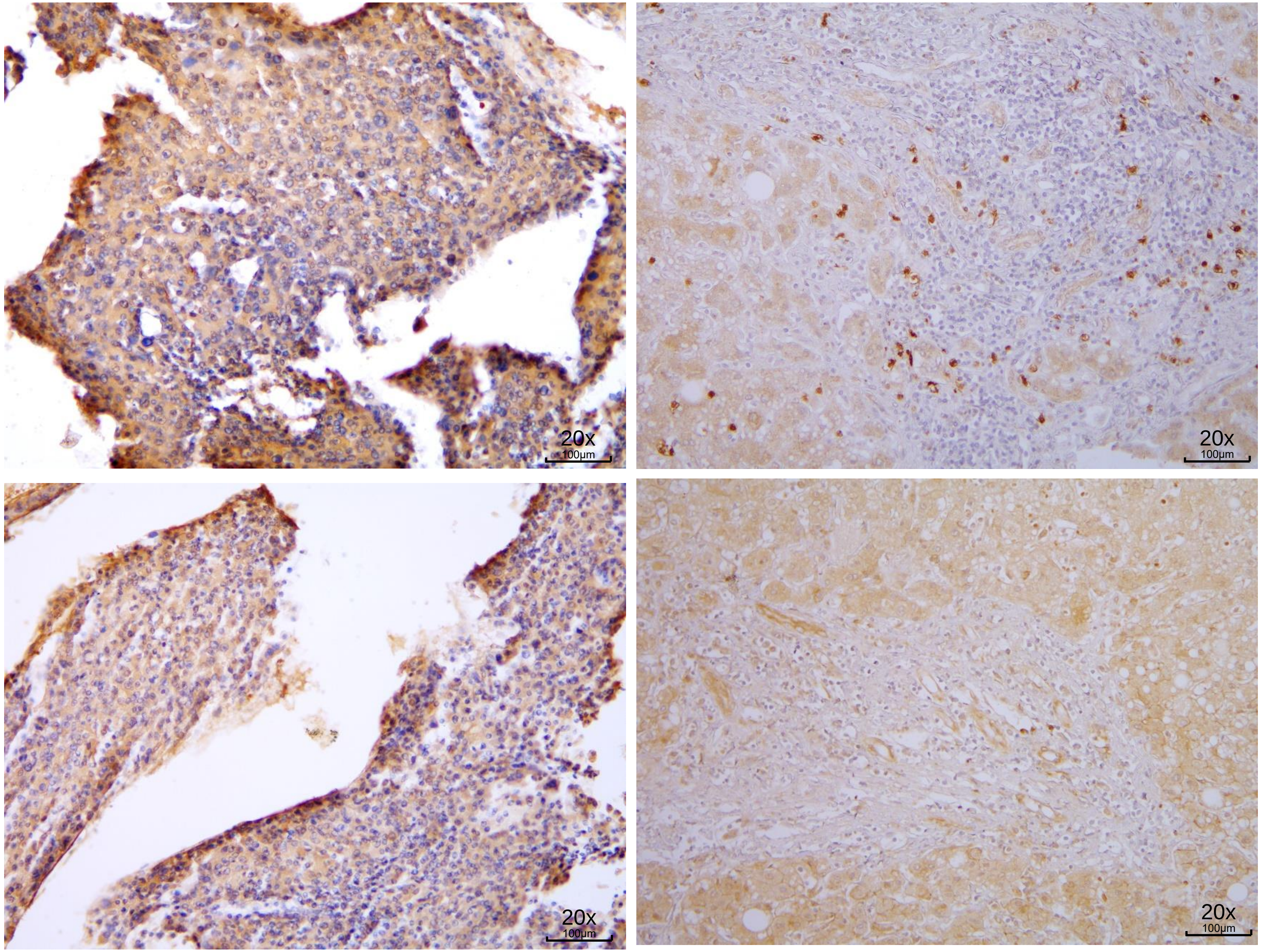

Low Ki67 expression

Patient 5

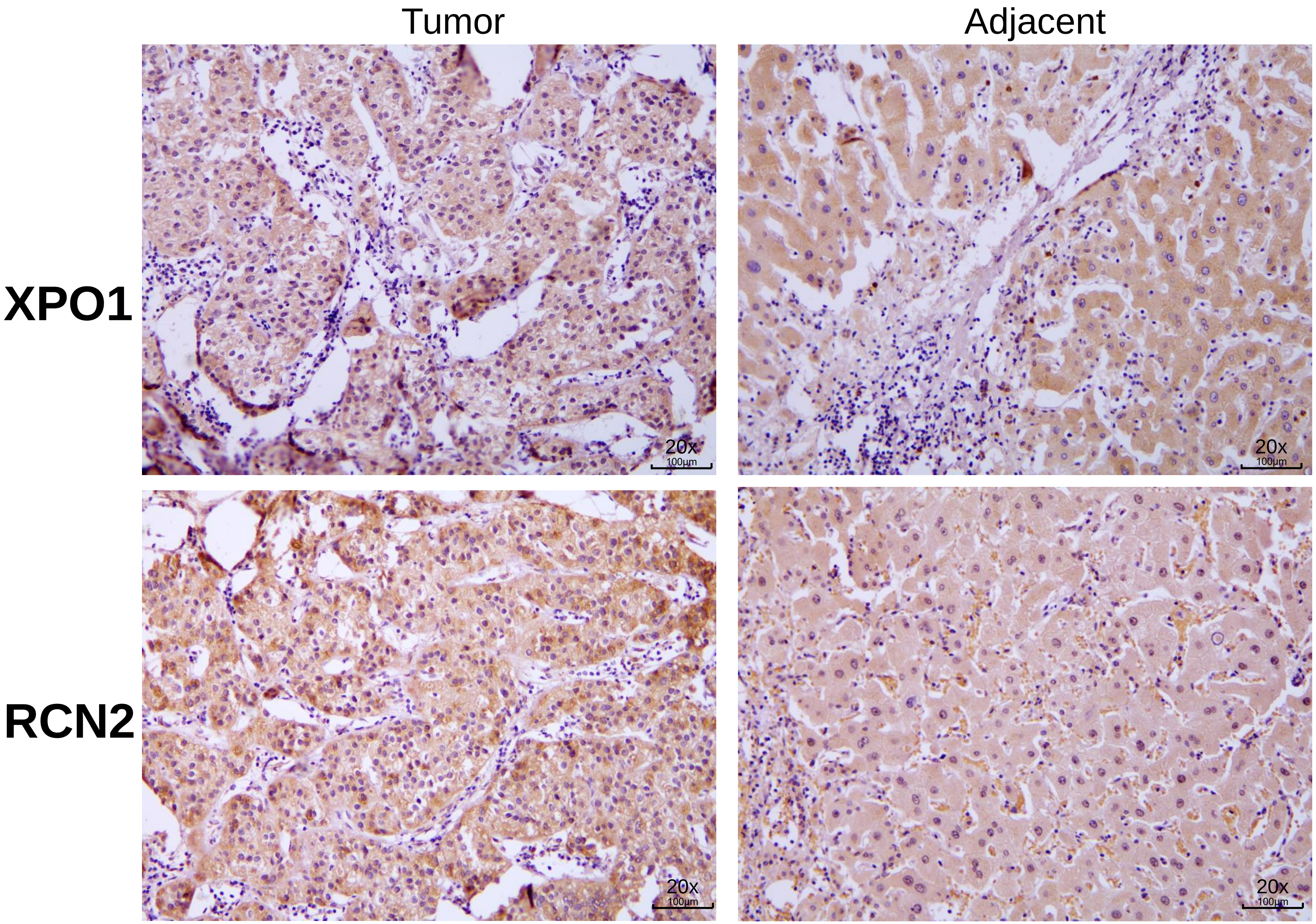

Low Ki67 expression

Patient 6

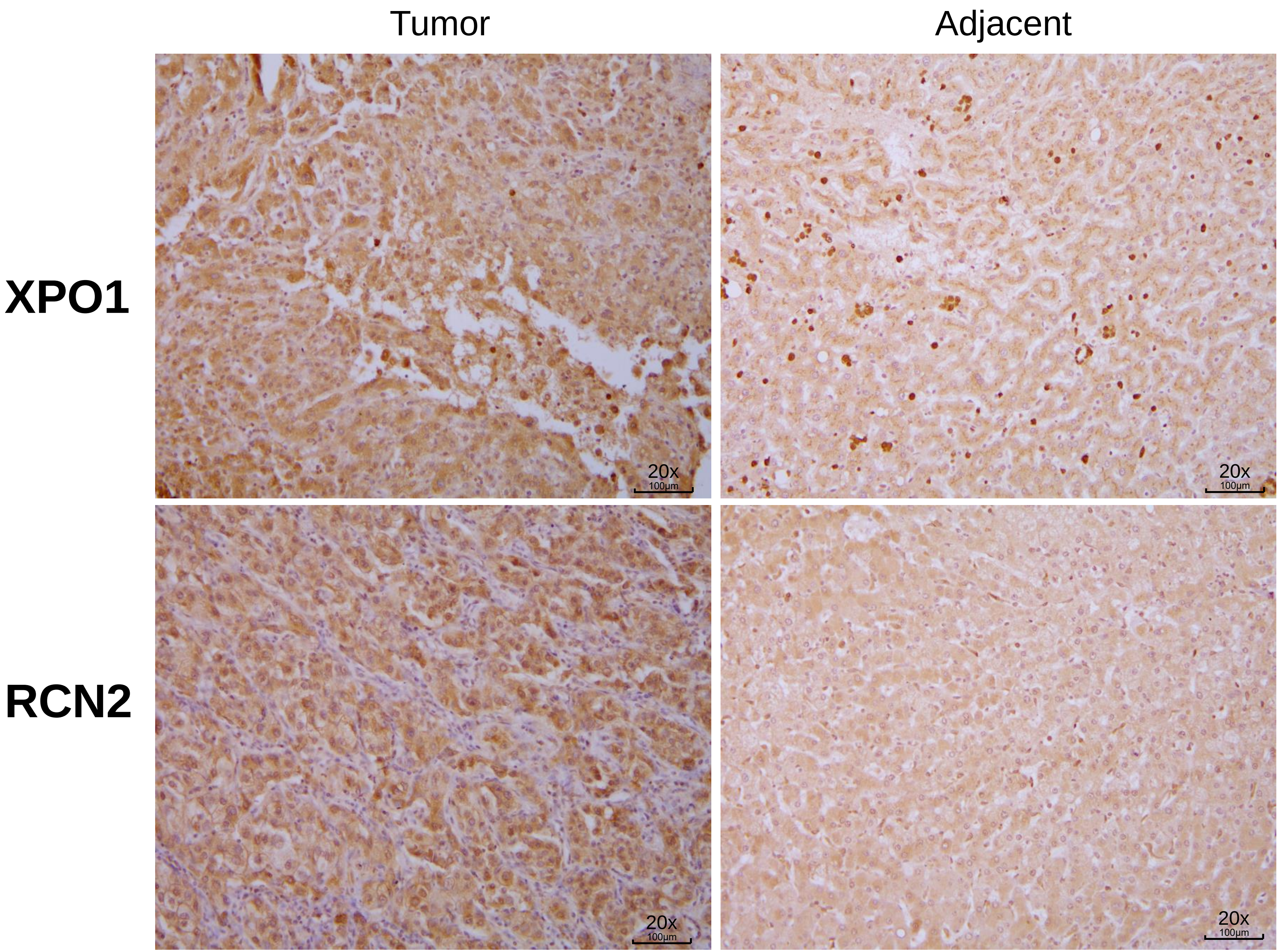

Low Ki67 expression

Patient 7

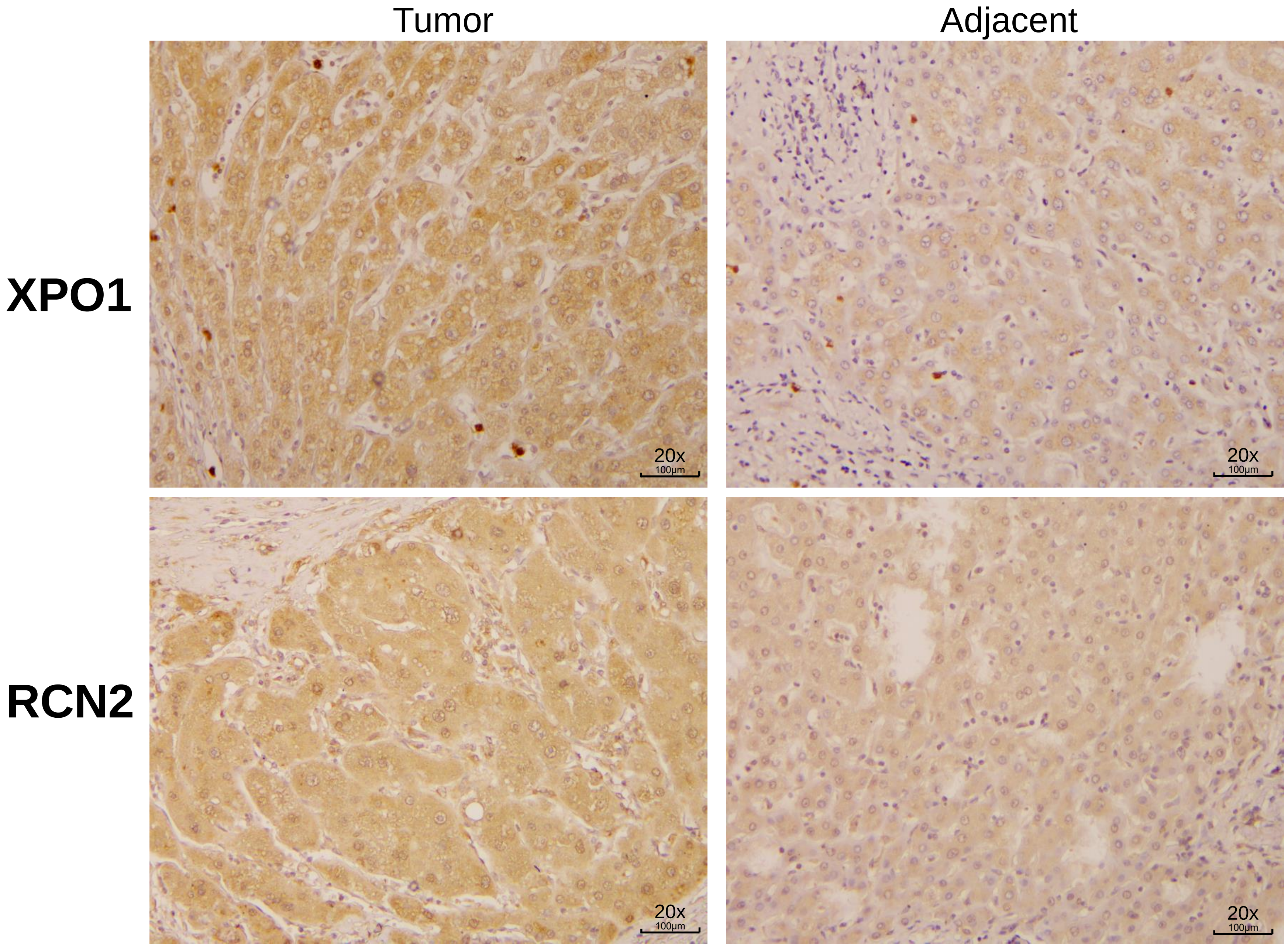

Low Ki67 expression

Patient 8

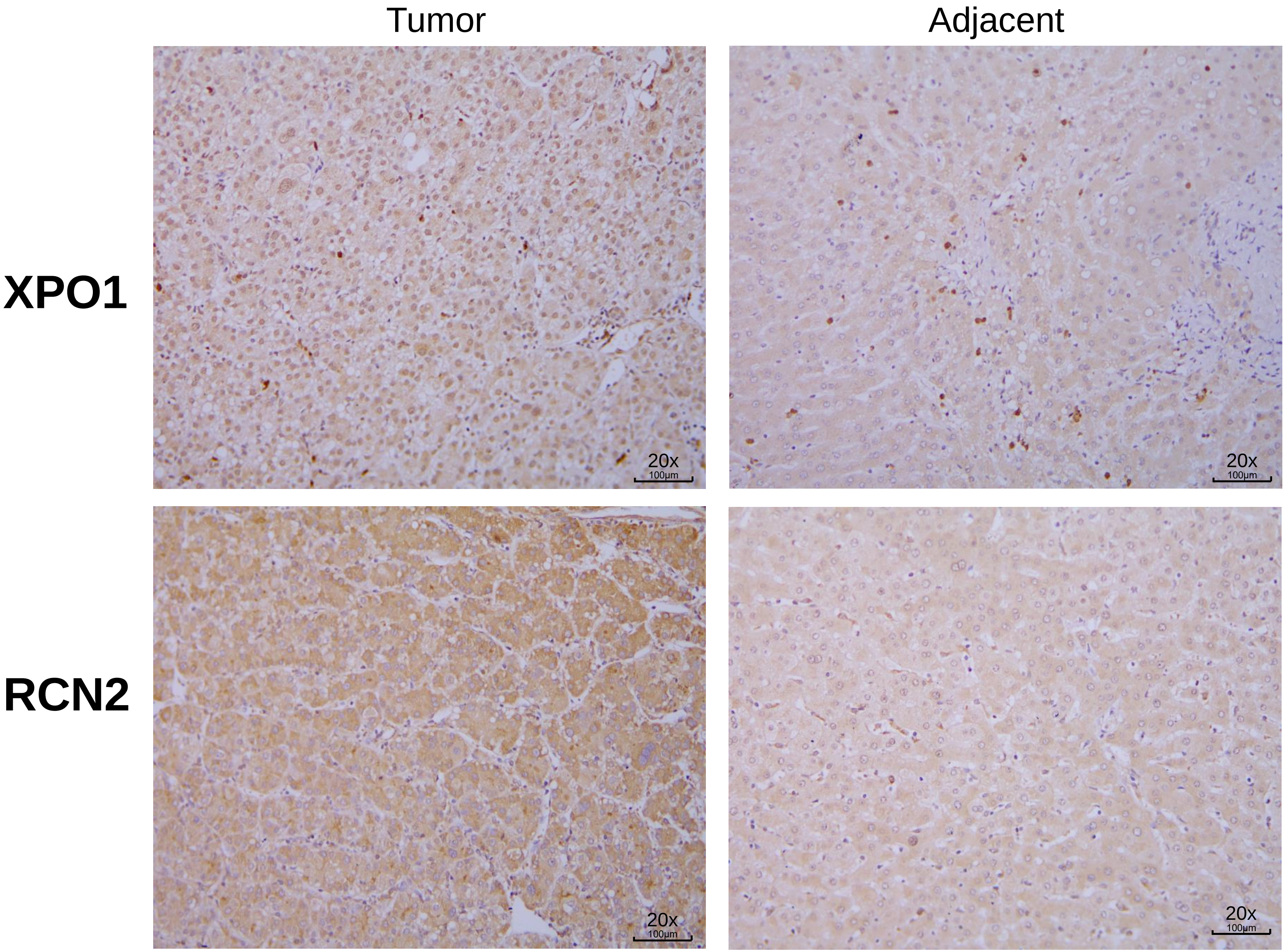

Low Ki67 expression

Patient 9

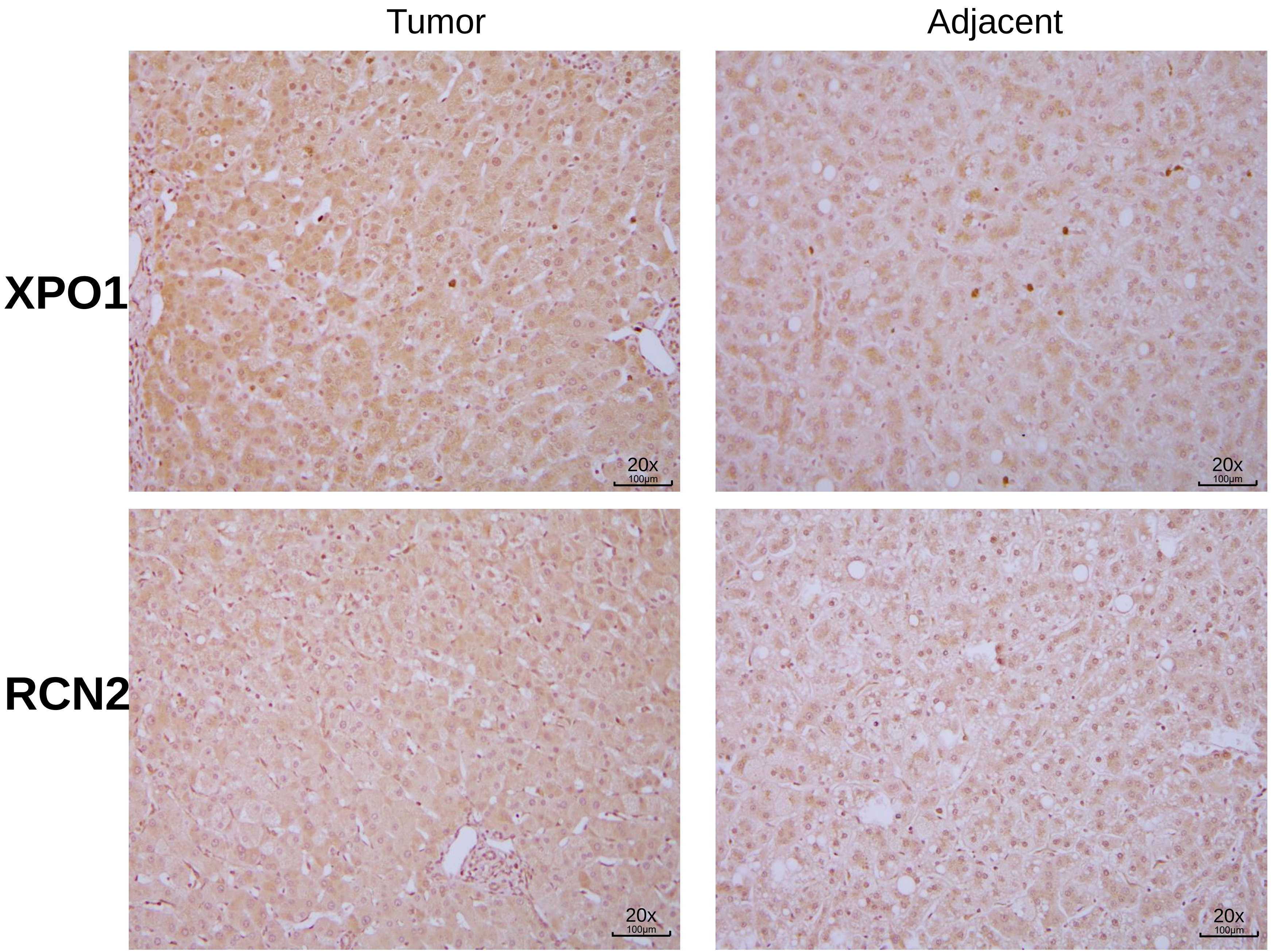

Low Ki67 expression

Patient 10

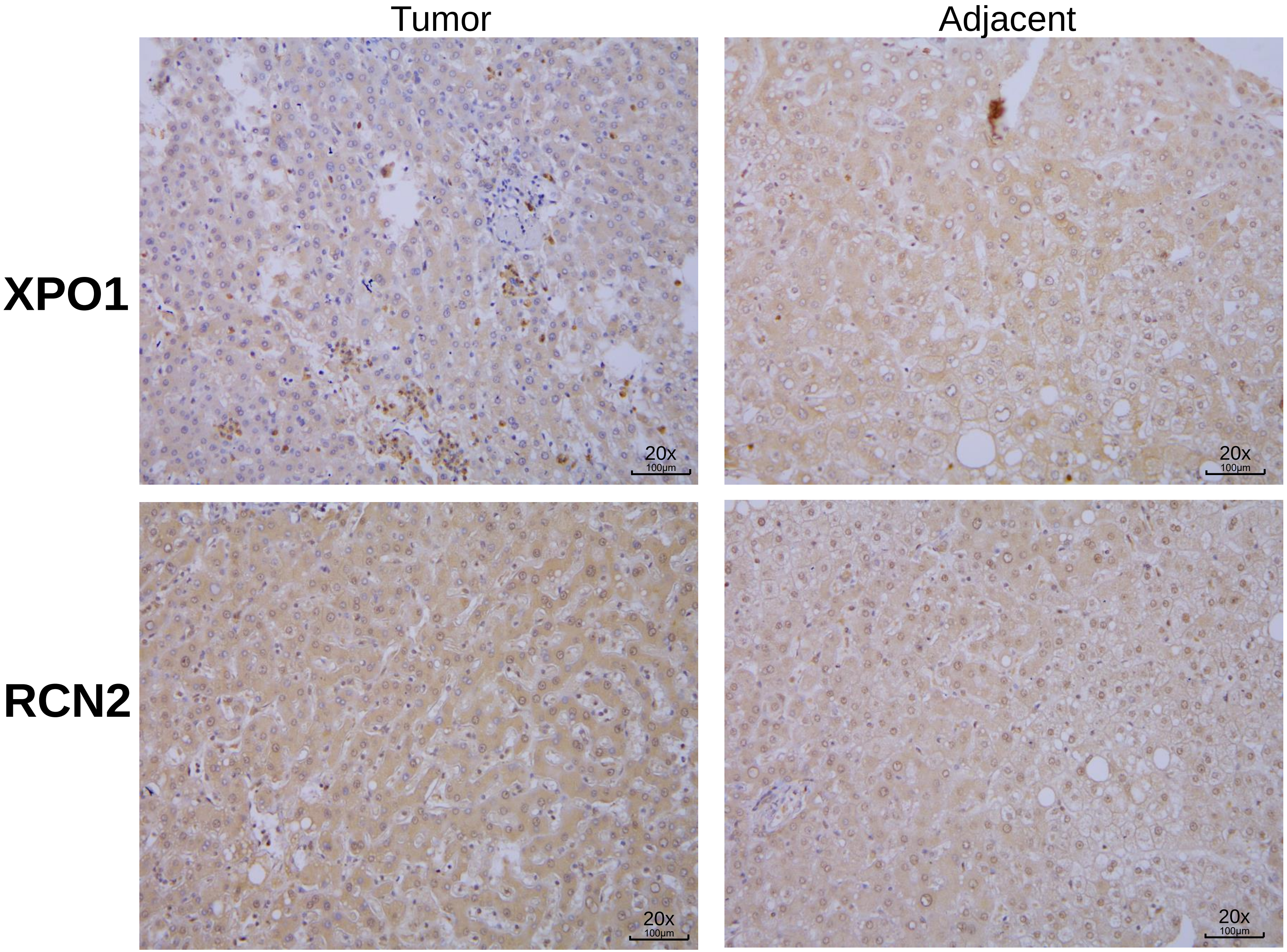

Low Ki67 expression

Patient 11

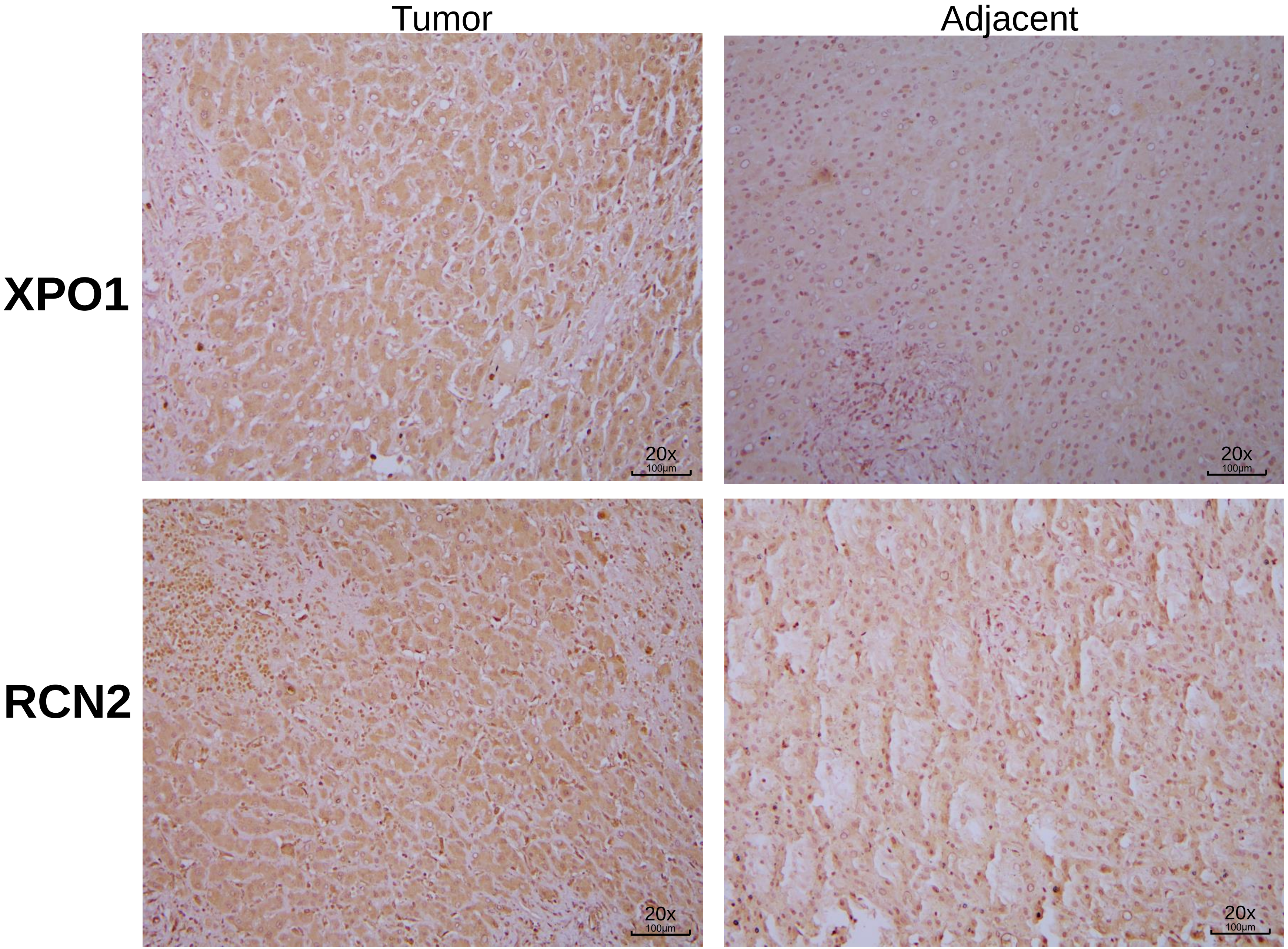

Low Ki67 expression

Patient 12

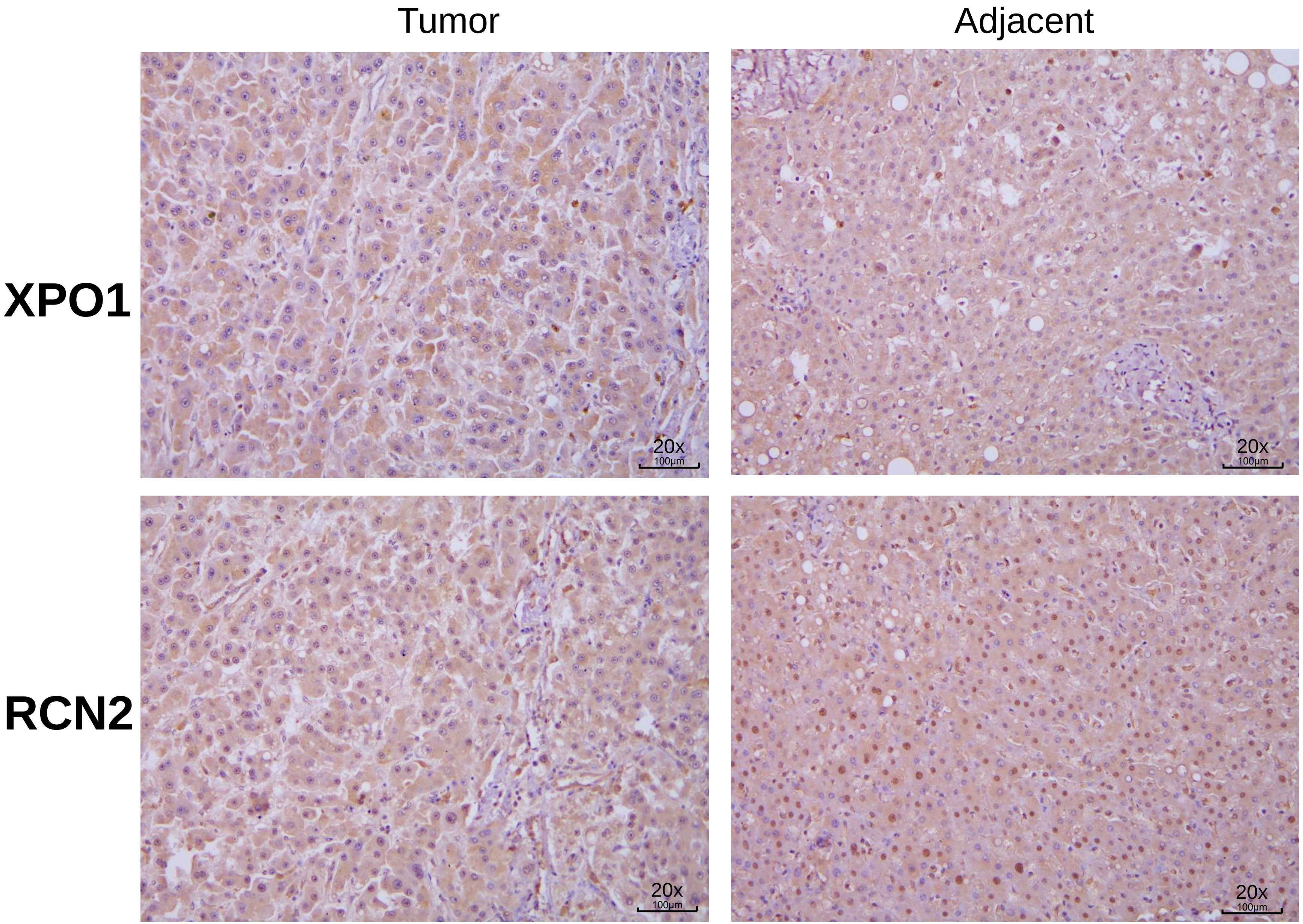

Low Ki67 expression

Patient 13

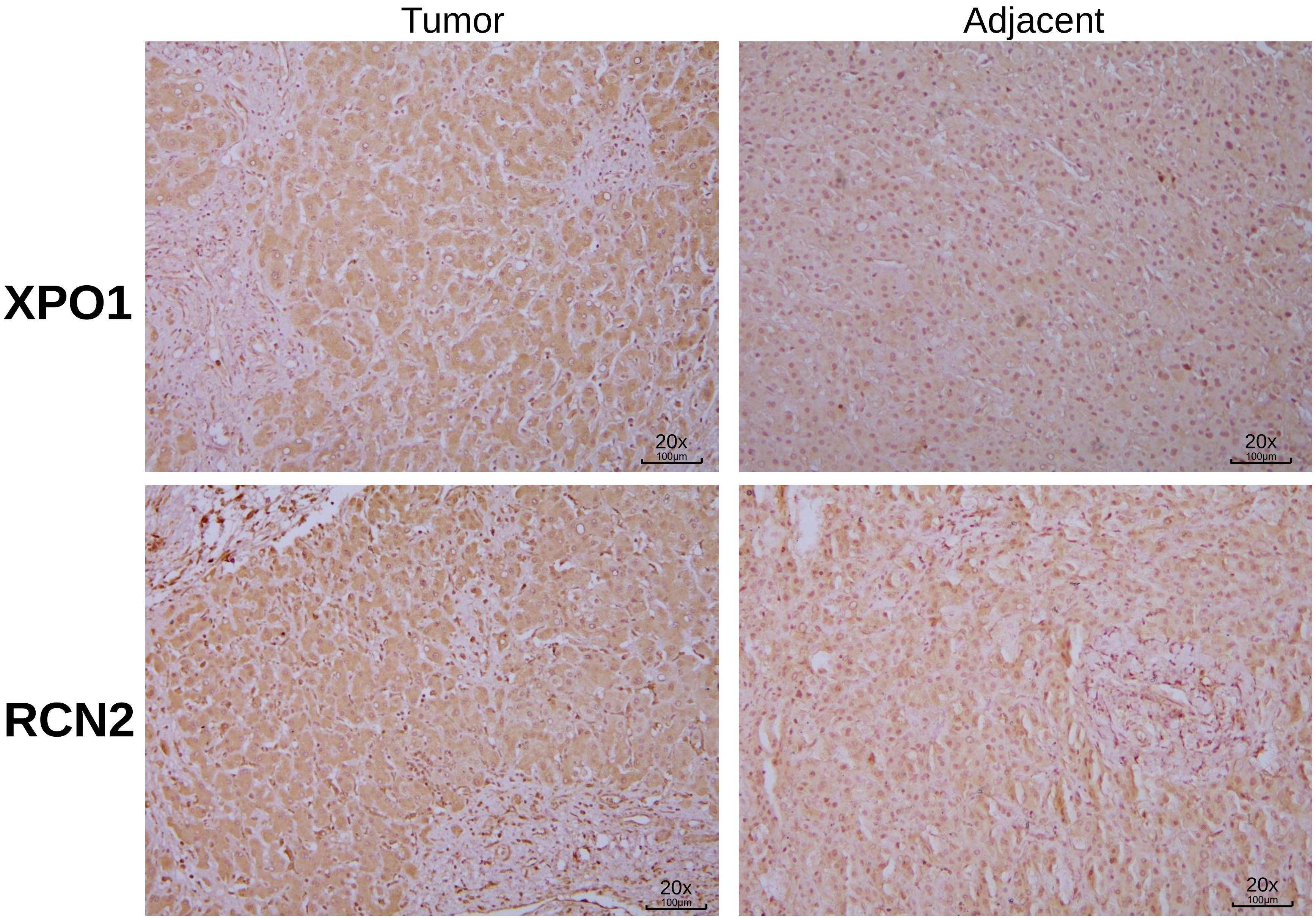

Low Ki67 expression

Patient 14

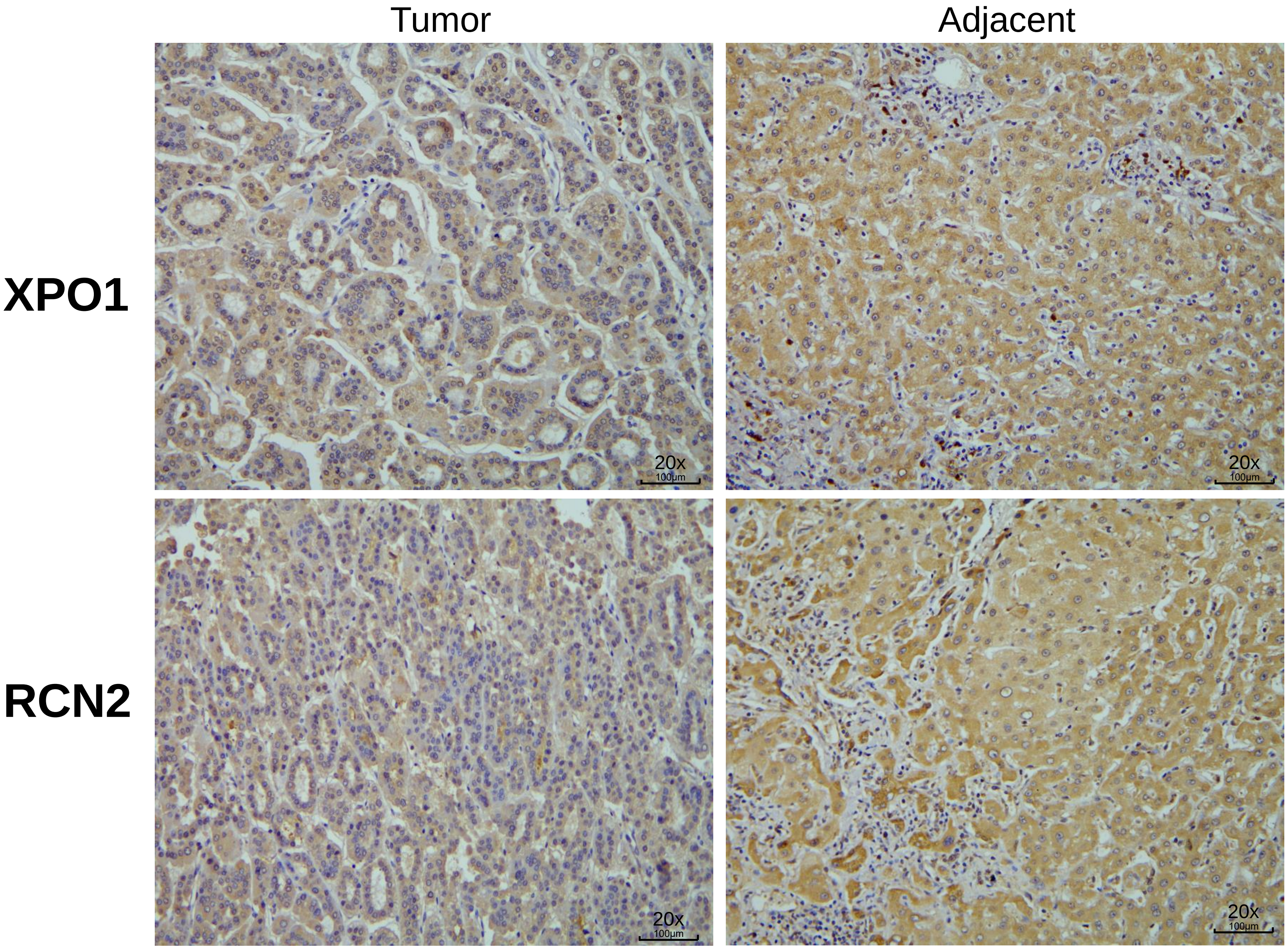

Low Ki67 expression

Patient 15

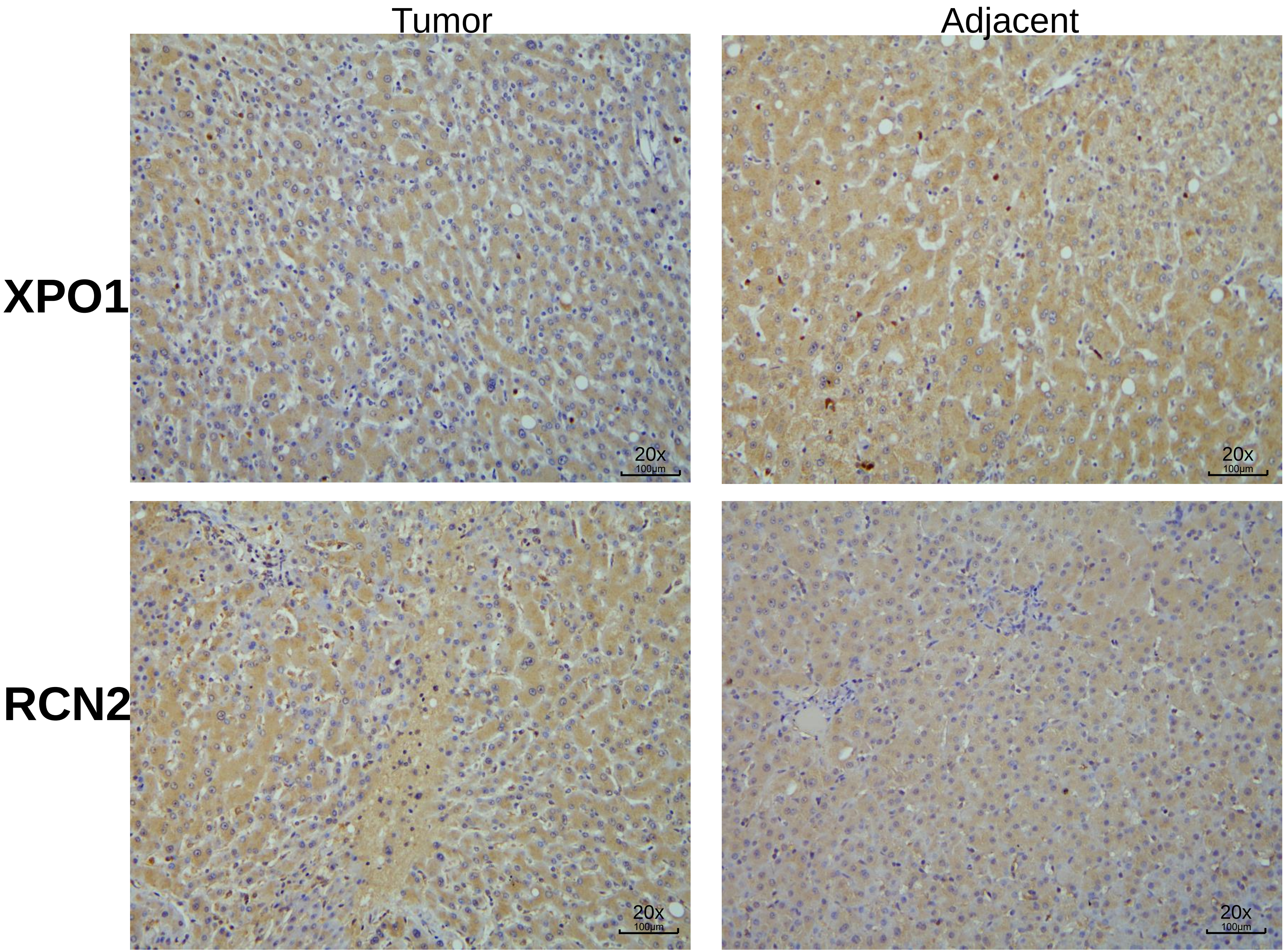

Low Ki67 expression

Patient 16

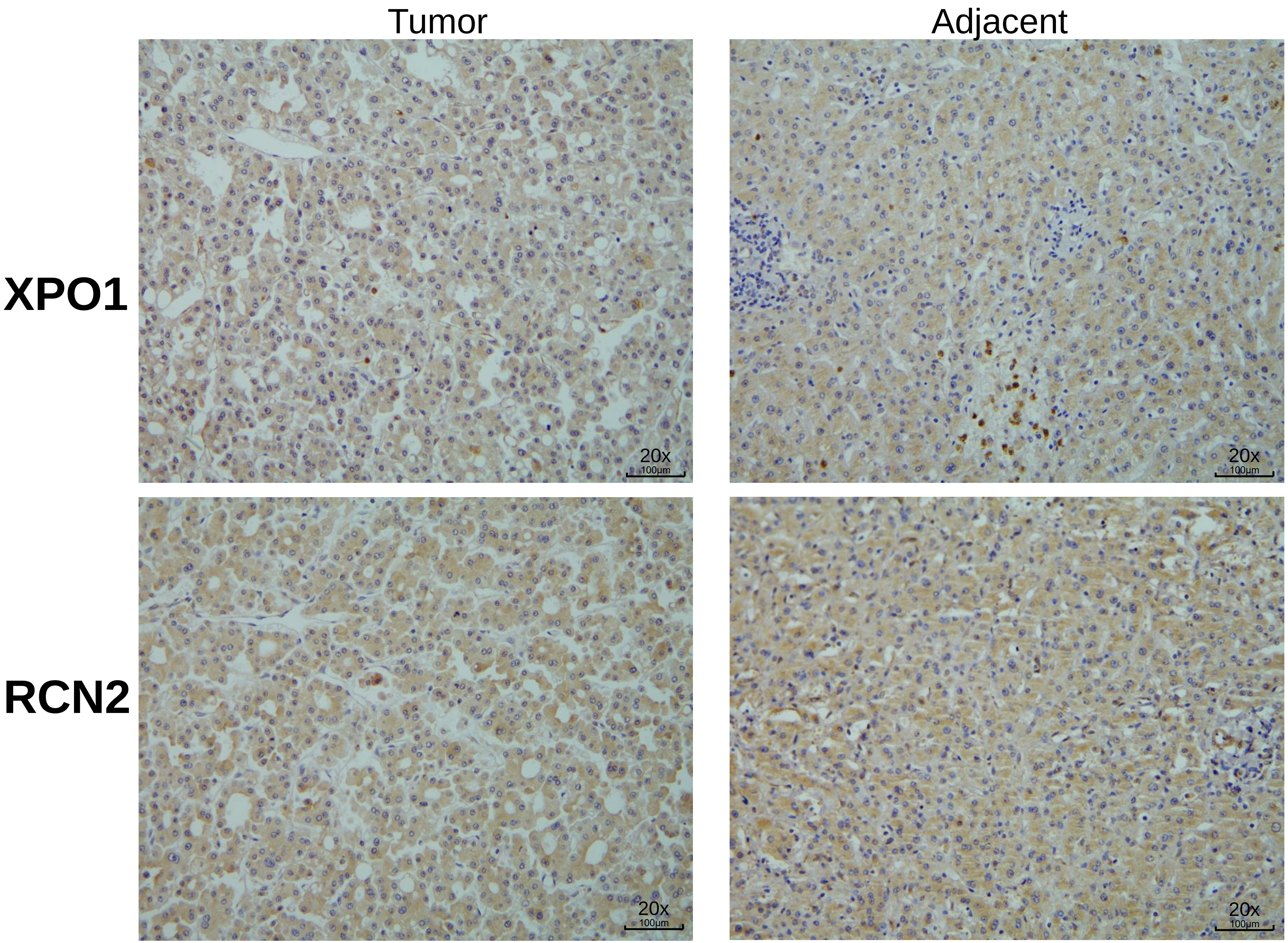

Low Ki67 expression

Patient 17

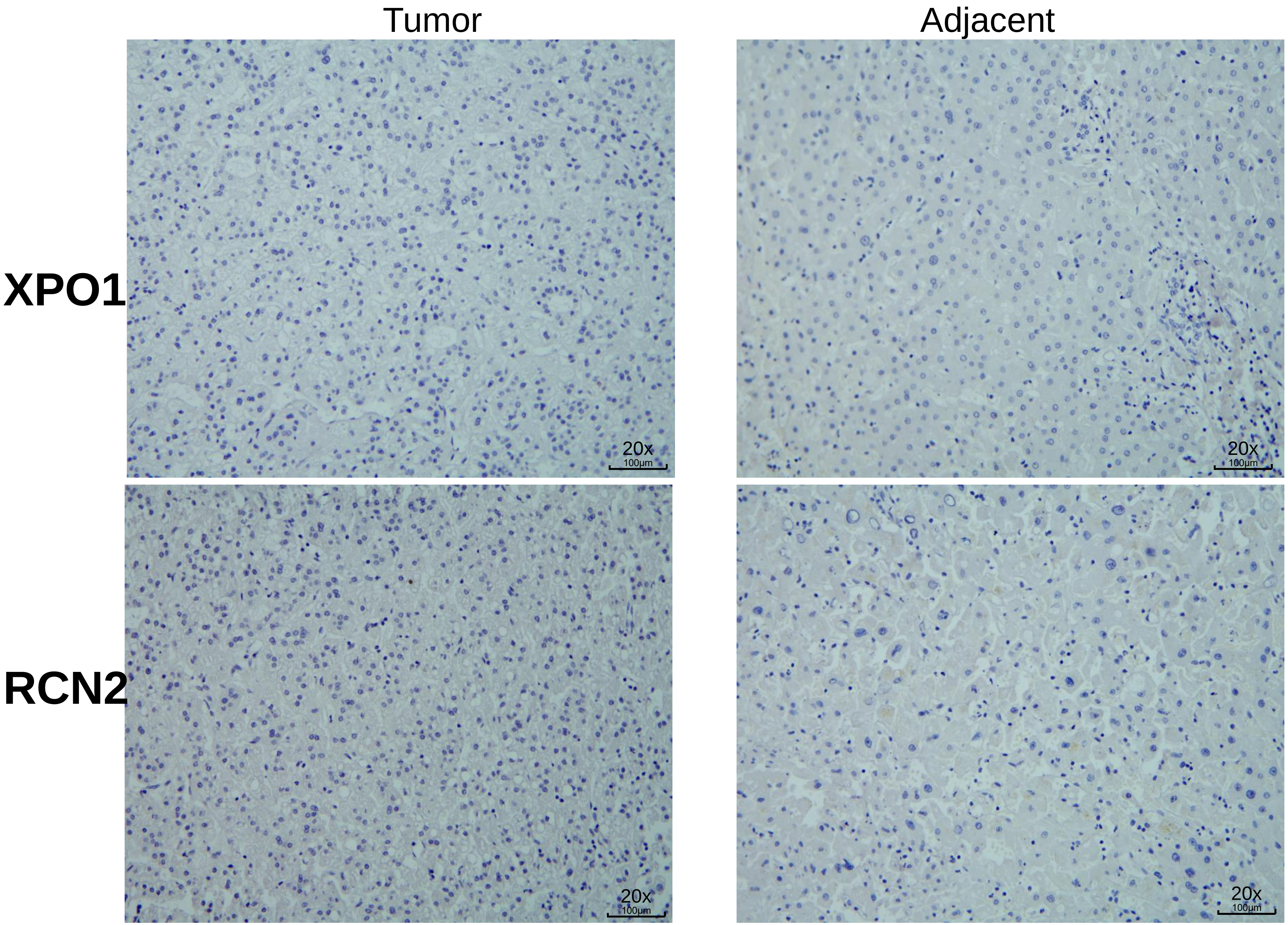

Supplement: Supplementary file 7 [file Supplementaryfile1.pdf]
